# NMR and DFT Studies on Solvation Phenomena in Bioorganic Molecules, Natural Products and Model Compounds: Current and Future Perspectives for Atomic-Level Structures and Mechanistic Catalytic Reactions

**DOI:** 10.3390/molecules31040703

**Published:** 2026-02-18

**Authors:** Michael G. Siskos, Ioannis P. Gerothanassis

**Affiliations:** Section of Organic Chemistry & Biochemistry, Department of Chemistry, University of Ioannina, GR-45110 Ioannina, Greece

**Keywords:** solvation, implicit solvation, explicit solvation, chemical shifts, DFT, bioorganic molecules, natural products

## Abstract

The structural role of solvation phenomena in bioorganic compounds has been documented sporadically over the last two decades, although they are of fundamental importance in a variety of chemical, physical, and biological processes. NMR chemical shifts depend on the electron densities around the nuclei, which can be influenced by the surrounding environment. Solvent-dependent chemical shift variations, therefore, can provide important structural information on solute–solvent interactions, especially nuclei, which belong to polar groups, such as OH, NH, CONH, COOH, etc. Recent developments in quantum chemical methods for calculating NMR chemical shifts, especially those incorporating explicit solvent effects, and the exponential advances in computer power can provide an excellent methodology for the accurate calculation of chemical shifts in solution. Furthermore, comparison of density functional theory (DFT) calculated activation free energies with NMR experimentally determined values can provide a reliable method for investigating the role of solvents in various atomistic reaction mechanisms. It has been demonstrated that the combined use of NMR and DFT calculations represents the new frontier of our understanding of the role of solvents, at the atomic level, in molecular structures and in catalytic reactions of bioorganic molecules, natural products and model compounds.

## 1. Introduction

The structure and dynamics of solvation phenomena are among the most attractive subjects in chemistry and in related scientific disciplines. Thus, numerous studies have been published since the 1930s, including a fairly large number of monographs summarizing the results of various scattering and spectroscopic methods, quantum chemical calculations, conformational analysis and structure–activity relationships [1,2,3,4,5,6,7]. The solvation/hydration structure of inorganic anions has been described in terms of the number of nearest neighboring solvation (H_2_O) molecules around the ion and their ion–solvent (H_2_O) distances. Although widely spread solvation (hydration) numbers have been reported for a given ion under similar conditions using a variety of experimental and computational methods, there is no question of the validity of the concept of the primary solvation (hydration) structure of inorganic ions [8,9,10].

Structural models of solvation phenomena of bioorganic compounds, on the contrary, are less conclusive although they are of fundamental importance in a variety of chemical, physical, and biological processes [1,4,11,12,13,14,15]. Single-crystal X-ray and neutron diffraction crystallography can provide high-resolution structural data on organic molecules that frequently co-crystalize in polymorphs with one or more solvent molecules (solvates), especially H_2_O (hydrates), which is a phenomenon of fundamental and practical importance. The solvent molecules fold together in the crystal lattice to compensate for a donor/acceptor imbalance and/or to fill the holes in a closed-packed crystal structure. Water is incorporated into organic crystals much more frequently than other common solvents, although it is not a particularly good solvent for organic compounds [16]. Several prediction rules of hydration in organic crystals have been reported and summarized in various articles [16,17,18,19]. How does the arrangement of solvent molecules, specifically H_2_O molecules, around any particular organic molecule progress our understanding of solvation processes in solution? The static structures obtained and the limited number of solvent molecules from crystalline samples do not provide sufficiently accurate descriptions of solvent binding and dynamics. These structures, therefore, allow us to examine the “freezing of solvent molecules and especially H_2_O molecules in thousands of molecular dimensions” [18].

Several spectroscopic and quantum chemical methods have been published in the last few decades to investigate the role of solvents in structural and conformational analysis, in diastereo- and enantio-selective reactions, and in the improvement of stereoselectivity, which could be of importance in structure–activity relationships and in bioorganic catalysis [1,2,3,4,11,12,13,14,15,20,21]. Several of the above advancements are summarized in the classical book by Reichart and Welton [4]. At a molecular level, solvents consist of individual solvent molecules, which are characterized by a variety of molecular and electronic properties, such as hydrogen bond donor (HBD) and hydrogen bond acceptor (HBA) properties, electron pair donor (EPD) and electron pair acceptor (EPA) properties, the dipole and quadrupole moment, electronic polarizability, etc. In order to take into account the complex intermolecular solute/solvent and solvent/solvent interactions, numerous multiparameter correlation equations and empirical solvent polarity scales for a variety of organic solvents have been developed [1,2,3,4,13]. Computational chemistry has also been used to explore experimental solvent basicity, acidity, and polarity/polarizability parameters [21]. While valuable for capturing bulk trends, these parameters are not sufficient to account for localized three-dimensional structural models of solvation phenomena [20]. As pointed out in a recent perspective by Reichardt, “The common classification of intermolecular and interionic forces is an artificial construct done by physical chemists in order to better understand and predict the rather complex and variable mutual interactions between solute and solvents particles in solution” [13].

Apart from X-ray and neutron diffraction experiments, scanning tunneling microscopy [22] and femtosecond X-ray liquidography [23], nuclear magnetic resonance (NMR) spectroscopy using NOEs, chemical shifts, and J-couplings can provide a rich source of information that can be leveraged as a molecular fingerprint for solvation phenomena [24]. Homonuclear 2D NOESY/2D ROESY and their 3D versions have been utilized to investigate long-lived internal individual molecules in H_2_O bound tightly to proteins, in the spine of hydration in DNA duplexes, and in the intermolecular interface of protein–DNA complexes [25,26]. The solvation of organic molecules can be investigated by intermolecular ^1^H NOESY experiments. Intermolecular NOEs, however, between organic solute molecules and solvent molecules, have received less attention since deuterated solvents are usually used, which results in very weak or unobservable NOE effects [27]. Nevertheless, homonuclear and heteronuclear Overhauser enhancement spectroscopy (HOESY), with specific pulse sequences to eliminate the unwanted residual H_2_O signal [28], has been utilized to detect space solute–solvent cross-peaks and, thus, the preferential solvation of a solute [27,28,29,30].

**Figure 1:**
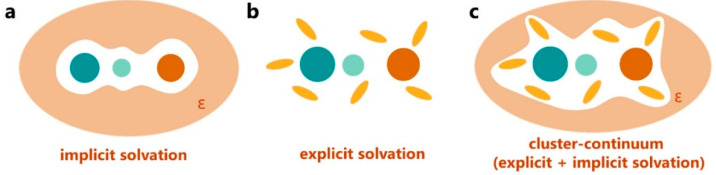
Schematic representation of models used to take into account solvation effects: (**a**) continuum solvation, (**b**) explicit solvation, and (**c**) cluster–continuum solvation. Reprinted with permission from [51]. Copyright 2025, The American Chemical Society, Washington, DC, USA.

NMR chemical shifts depend on the electron densities around the nuclei, which can be influenced by the surrounding environment. Solvent-dependent chemical shift variations in solution can provide important structural information on solute–solvent interactions, especially for nuclei that belong to polar groups, such as OH, NH, CONH, COOH, etc. Developments in quantum chemical methods for calculating NMR chemical shifts and the exponential advances in computer power have led to an increasing number of studies that combine calculations with experimental chemical shift data [31,32,33,34,35,36,37,38,39]. However, in order to employ quantum chemical methods, the error between experimental and computational chemical shifts needs to be significantly less than 0.5 ppm for protons and 2–3 ppm for heteroatoms, such as ^13^C, ^15^N, ^17^O, ^31^P, ^19^F, etc. Numerous publications have been performed to assess the accuracy of quantum chemical methods using a broad range of DFT functionals and basis sets vs. computational efficiency [34,40,41,42,43,44,45,46]. The accurate calculation of chemical shifts in solution, however, places severe demands on how individual solvent molecules interact with solute molecules. In principle, there are three methods for incorporating solvation effects in calculations of NMR chemical shifts (Figure 1):

(i) Implicit solvation models, which treat the solvent as a continuous dielectric medium surrounding the solute molecule. Typical examples include the polarizable continuum model (PCM) [47], the integral equation formalism PCM (IEF = PCM) [48] model, the conductor-like polarizable continuum model (CPCM) [49], the conductor-like screening model (COSMO), and the conductor-like screening model for real solvents (COSMO-RS) [50]. The solvent is treated as a conductor (ε = ∞), and a scaling factor is used for a given solvent.
(ii) Solvation models, which include explicit solvent molecules in the calculation. This can be done using molecular dynamics simulations or manually placing solvent molecules in the vicinity of potential polar or charged sites of the solute.
(iii) Hybrid models, which combine implicit and explicit solvation. This can be done by including a limited number of explicit solvent molecules around, e.g., polar or charged groups, and by using an implicit model for the bulk solvent (Figure 1).

Despite the considerable progress in incorporating solvation models in quantum chemical calculations and the significant growth of experimental NMR chemical shifts in various solvents, the structural role of solvation phenomena in bioorganic molecules, natural products, and model compounds has been documented sporadically over the last two decades. In several publications, emphasis has been given to improving the agreement between computational and experimental data rather than evaluating the results in terms of complex solvatomer structures. The objective of this perspective is to increase awareness on the importance of DFT calculations of solvent effects on chemical shifts in various classes of organic molecules, to serve as a practical guide for physical organic chemists and NMR experimentalists who wish to pursue NMR calculations of solvation phenomena, and to understand the complex solvatomer structures in terms of noncovalent interactions (hydrogen bonding, donor–acceptor and van der Waals interactions). We will not attempt to provide a discussion on the theoretical foundations of computational NMR parameters, since numerous comprehensive review articles have been published [31,32,33,34,35,36,37,38,39]. Emphasis will be given to:

(i) A systematic investigation of the factors that determine the accuracy of the chemical shift computations of ^1^H NMR of labile hydrogens, ^14,15^N, ^17^O, and ^31^P NMR.
(ii) The role of explicit inclusion of solvent molecules in the calculation of chemical shifts, including the effects of increasing the number of explicit solvent molecules and the cooperative hydrogen bonding effect, using reliable and fast methods.
(iii) The effects of the choice of the density functional, the size of the basis set, the effect of zero-point vibrational correction and the importance of geometry optimization in DFT calculations.
(iv) The dynamic nature of molecules in solutions results in chemical shifts that are weighted averages of all accessible low-energy solvation structures, which can significantly add to the computational cost.
(v) A systematic investigation of the role of solvents in various atomistic reaction mechanisms with the combined use of calculated and experimentally determined activation free energies.

Most publications quoted in this paper were published in the last 15 years; however, some earlier important monographs and articles are also discussed.

## 2. ^1^H NMR and DFT Studies of Solvation Phenomena in Labile Hydrogens

**Figure 2:**
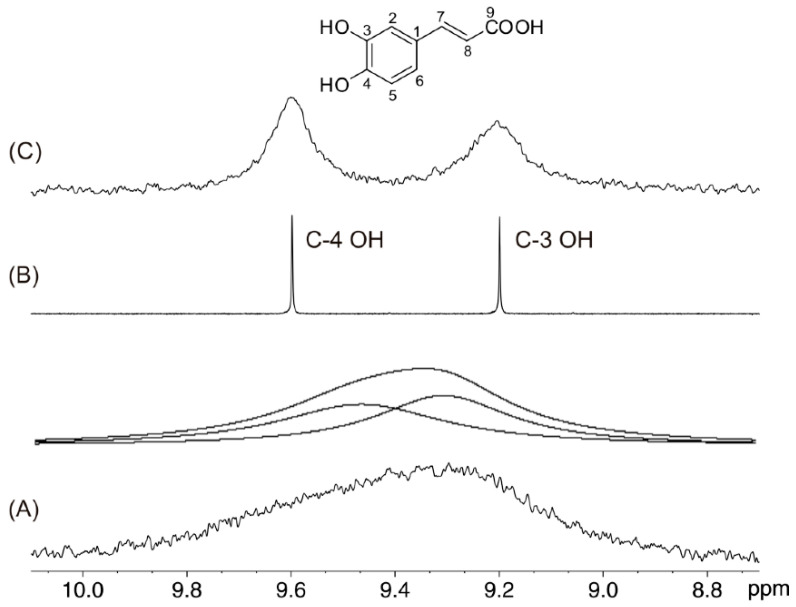
A 500 MHz ^1^H NMR spectra of the hydroxyl group region of caffeic acid (C = 5.7 × 10^−3^ M, T = 292 K, number of scans = 32), in (**A**) DMSO-d_6_; upper trace: simulated line-shapes with two Lorentzian peaks for C-4 OH and C-3 OH, having linewidths of 203 Hz and 164 Hz, respectively, using the Bruker peak-fitting routine; (**B**,**C**) with a molar ratio [picric acid]/[caffeic acid] of 12 × 10^−3^ and 219 × 10^−3^. Reproduced with permission from [53]. Copyright 2011, the American Chemical Society, Washington, DC, USA.

Hydrogens bonded to oxygen (e.g., phenolic OH, alcoholic OH, carboxylic COOH, and hydroperoxide O-O-H), nitrogen (e.g., amide CONH and amine NH), sulfur (e.g., SH and SOOH), and phosphorus (PH), undergo chemical exchange with hydrogens in protic solvents (e.g., H_2_O and MeOH), or with trace amounts of H_2_O present in aprotic solvents. Exchangeable protons [52,53,54,55,56,57,58] usually appear as broad signals with a variety of chemical shifts that can cover several ppm [59,60,61,62,63,64]. Several developments, however, in the optimization of experimental parameters, such as pH, temperature, and nature of the solvent, can be utilized to obtain sharp ^1^H NMR resonances of labile hydrogens. Figure 2 shows the ^1^H NMR spectrum of the phenolic OH region in caffeic acid. The C-4 OH and C-3 OH groups appear as very broad and strongly overlapped resonances with Δν_1/2_ > 160 Hz. The addition of the appropriate amount of picric acid (pK_a_ = 0.42) results in a very effective linewidth sharpening (Δν_1/2_ < 2 Hz), which allows for the application of ^1^H-^13^C HMBC experiments for resonance assignment and accurate determination of chemical shifts. Due to contemporary safety concerns about picric acid, it has been suggested to use of trifluoroacetic acid (TFA), pK_a_ = 0.45, which is a common reagent in organic synthesis and has shown to be very effective in reducing exchange broadening effects [57,65].

Excellent ^1^H NMR sensitivity, when sharp resonances of the labile hydrogens can be obtained, allows for very low solute concentrations (<1 mM) to be used. Solvent-dependent chemical shifts, therefore, can be attributed to solvation phenomena rather than to concentration-dependent aggregation processes. The above advantages, coupled with the large chemical shift deshieldings, up to 21 ppm for ionic compounds, can provide an excellent means to investigate intra- and inter-molecular hydrogen bonding and solvation phenomena at the atomic level [55,59,60,61,62,63,64].

### 2.1. Phenols

**Figure 3:**
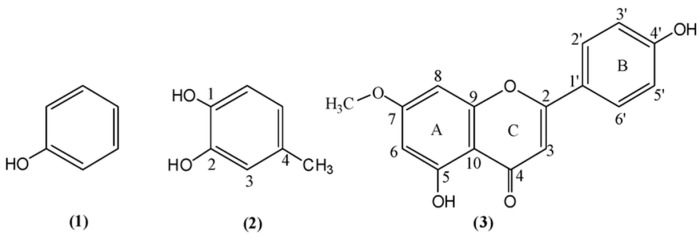
Chemical formulas of phenol (**1**), 4-methylcatechol, (**2**) and genkwanin (**3**). Reproduced with permission from [59]. Copyright 2013, The Royal Society of Chemistry, London, UK.

**Figure 4:**
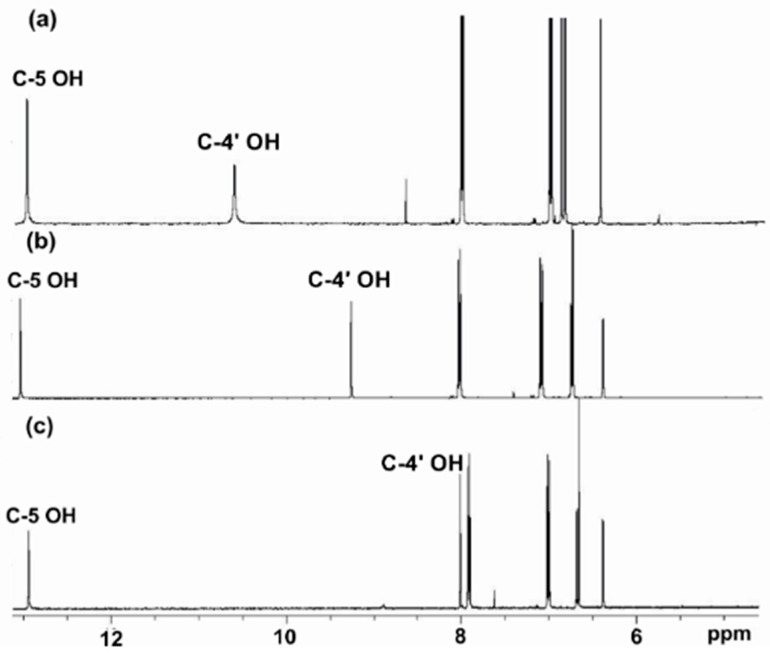
^1^H NMR spectra (400 MHz, T = 298 K, number of scans = 8 to 128, total experimental time = 1 to 15 min) for genkwanin (**3**) in (**a**) DMSO-d_6_ (C = 5 mM), with the addition of picric acid (4 μL of 10 mM), (**b**) acetone-d_6_ (C = 5 mM), with the addition of picric acid (2 μL of 8 mM), and (**c**) CD_3_ CN (C = 5 mM), with the addition of picric acid (4 μL of 8 mM). Reproduced with permission from [59]. Copyright 2013, The Royal Society of Chemistry, London, UK.

It has been demonstrated that high-resolution ^1^H NMR resonances (Δν_1/2_ < 3 Hz) of the phenol OH groups can be obtained with the use of dry non-protic solvents, such as DMSO-d_6_, the progressive addition of trifluoroacetic acid (TFA) [57,58], and decreasing the temperature of the sample. Siskos et al. [59] reported experimental and computational chemical shifts in phenol, **1**, 4-methylcatechol, **2**, and the natural product genkwanin, **3** (Figure 3) in various solvents. The experimental chemical shifts in phenolic OH groups strongly depend on the hydrogen bond ability of the solvent: δ(DMSO-d_6_) = 9.36 ppm, δ(acetone-d_6_) = 8.29 ppm, δ(CD_3_CN) = 6.90 ppm, and δ(CDCl_3_) = 4.65 ppm (Table 1). The chemical shifts in the 5-OH group of genkwanin are essentially independent of the hydrogen bond ability of the solvent (Figure 4) due to a strong intramolecular 5-OH···OC-4 hydrogen bond interaction. On the contrary, the C-4′ OH chemical shifts strongly depend on the hydrogen bond ability of the solvent; thus, experimental chemical shift differences Δδ (δ_DMSO-d6_–δ_CDCl3_) up to 5.20 ppm have been observed (Table 1). This clearly demonstrates that ^1^H NMR of phenolic OH groups can provide an excellent method to investigate solvent effects at the atomic level.

**Figure 5:**
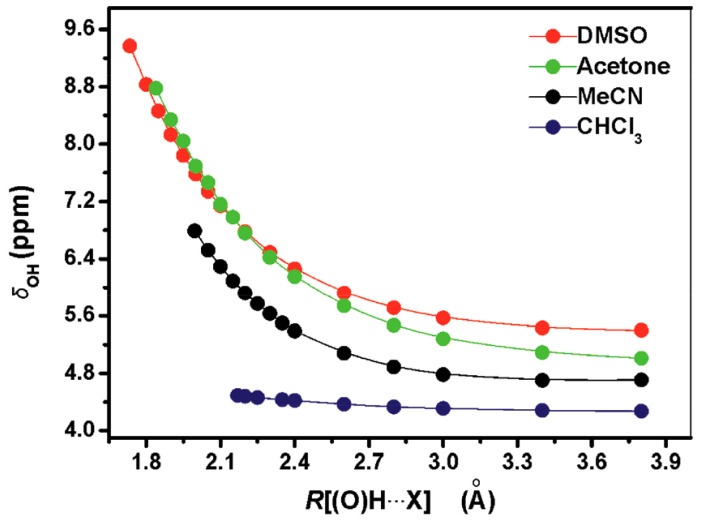
The dependence of the OH proton chemical shifts, δ_OH_ (ppm), in the 1:1 phenol (**1**)+solvent complexes vs. the distance R[(O)H···X] (X= O for DMSO (red) and acetone (green), X= N for MeCN (black), and X= C for CHCl_3_ (blue), without changing any other structural parameter. The calculations were carried out at the B3LYP/6-311++G(d,p) level. Reproduced with permission from [59]. Copyright 2013, The Royal Society of Chemistry, London, UK.

**Figure 6:**
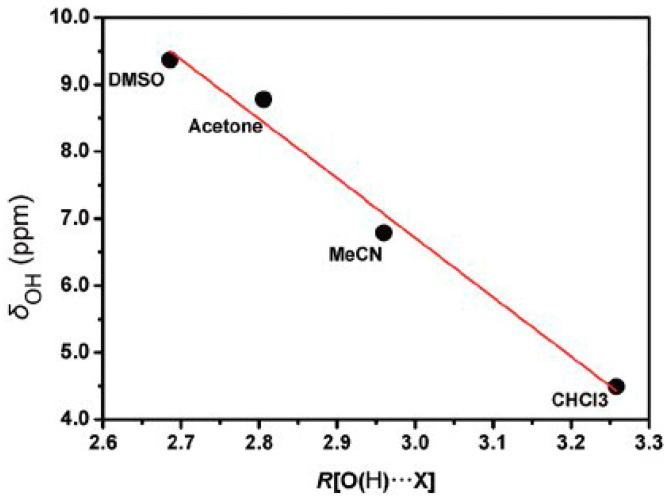
The dependence of the OH proton chemical shift, δ_OH_ (ppm), in the minimum energy conformer, optimized at the B3LYP/6-311++G(d,p) level of theory, in 1:1 phenol (**1**) + solvent complexes vs. R[O(H)…X] distances (X = O for DMSO and acetone, X = N for MeCN, and X = C for CHCl_3_). Reproduced with permission from [59]. Copyright 2013, The Royal Society of Chemistry, London, UK.

DFT calculations (at the B3LYP/6-311++G(d,p) level of theory) of 1:1 PhOH + solvent (DMSO, acetone, acetonitrile, and CHCl_3_) complexes showed a very significant effect on the increasing of the (O)H···X hydrogen bond distance, with the exception of the 1:1 PhOH + CHCl_3_ complex (Figure 5). At distances ≤ 2.1 Å, the plots could be reproduced by a linear equation. A strong linear dependence of −8.9 ppm Å^−1^ (R^2^ = 0.977) (Figure 6) was also observed for the minimum energy conformers of 1:1 phenol + solvent complexes vs. R [(O)H···X] distances (X=O for DMSO and acetone, X=N for MeCN, and X=C for CHCl_3_).

The DFT method with various basis sets resulted in excellent agreement between the calculated OH chemical shifts and the experimental data when using discrete 1:1 PhOH + solvent complexes (Table 2). It was concluded that larger basis sets are not necessary to accurately reproduce experimental chemical shift data. Thus, although a significant error may exist for a given method in the calculation of chemical shifts, the use of the same method for the reference compound results in noticeable error cancellation [34]. On the contrary, the calculated chemical shifts using the polarizable continuum model (PCM) in solvents capable of hydrogen bond interactions (δ_OH_(DMSO) = 4.44 ppm, δ_OH_(acetone) = 4.42 ppm, and δ_OH_(MeCN) = 4.44 ppm) strongly deviated from the experimental data (δ_OH_(DMSO-d_6_) = 9.36 ppm, δ_OH_(acetone-d_6_) = 8.29 ppm, and δ_OH_(MeCN) = 6.90 ppm) (Table 2). This reflects the more complex polar and hydrogen bond interactions in the above solvents, which are not adequately captured by the PCM model. Adding extra polarization and dispersion functions does not increase the prediction accuracy.

**Figure 7:**
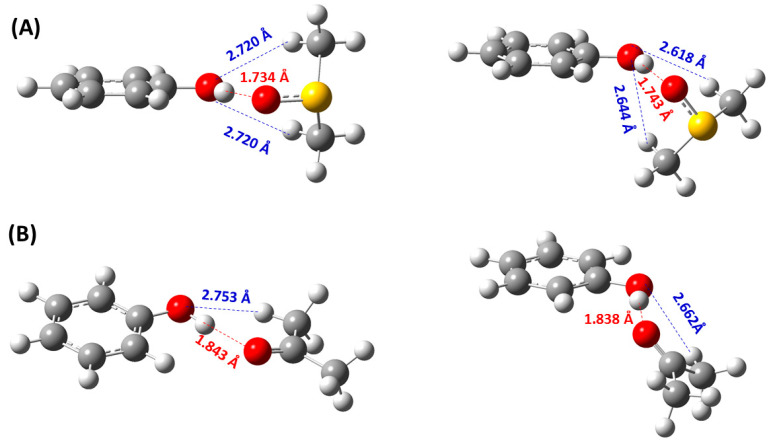
(**A**) The PhOH+DMSO 1:1 complex optimized at the B3LYP/6-311++G(d,p) (**left**) and B3LYP/6-31G(d) levels of theory (**right**). (**B**) The PhOH + acetone 1:1 complex optimized at the B3LYP/6-311++G(d,p) (**left**) and at the B3LYP/cc-p-VTZ levels of theory (**right**). Reproduced with permission from [59]. Copyright 2013, The Royal Society of Chemistry, London, UK.

In the phenol (**1**) + DMSO complex (Figure 7A), the OH and S=O groups and the aromatic ring were found to be in the same plane, at the B3LYP/6-311++G(d,p) level. The O-H···O=S bond distance of 1.734 Å indicates a strong hydrogen bonding interaction. The two CH_3_–groups of DMSO, which are perpendicular and symmetrical to this plane, form secondary interactions with the phenolic oxygen (O···H–C distance of 2.720 Å). Using the low computational cost basis set 6-31G(d) without diffuse functions, the O=S bond of the DMSO molecule deviated strongly from the phenol plane (~58°) (Figure 7A). In the phenol (**1**) + acetone complex, the plane of the acetone molecule deviated from the plane of the aromatic ring, with a dihedral angle of C1-O-H···O, which strongly depends on the basis set used for energy minimization (166.4° and 129.3° at the 6-311++G(d,p) and cc-pVTZ basis sets, respectively) (Figure 7B).

**Figure 8:**
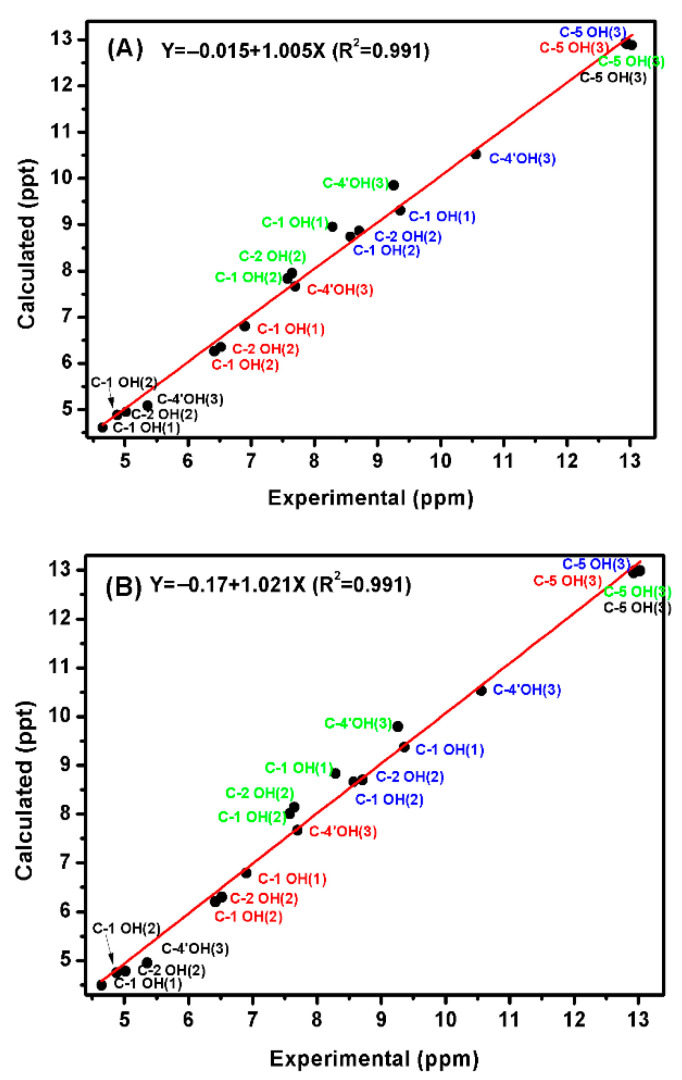
Calculated (at the B3LYP/6-311++G(2d,p) level of theory) vs. the experimental values of the chemical shifts in the –OH protons in phenol (**1**), 4-methylcatehol (**2**), and genkwanin (**3**) (Figure 3) in the different solvents, with minimization of the solvation complexes at the B3LYP/6-31+G(d) (**A**) and B3LYP/6-311++G(d,p) (**B**) levels of theory. Adopted with permission from [59]. Reproduced 2013, The Royal Society of Chemistry, London, UK.

Comparison between the calculated and experimental chemical shifts in phenol, **1**, 4-methylcatechol, **2**, and genkwanin, **3** (Figure 3) in DMSO, acetone, acetonitrile, and chloroform, shows excellent correlation with R^2^ = 0.991 at the B3LYP/6-31+G(d) and B3LYP/6-311++G(d) levels of theory (Figure 8). It was concluded that larger basis sets for energy minimization are not necessary for accurate ^1^H NMR chemical shifts [59].

Cooperativity in hydrogen bonding is a phenomenon of great importance in donor–acceptor complexes, since the formation of a hydrogen bond strengthens or weakens nearby hydrogen bonds [66,67,68,69]. Lomas and Rosenberg [70] investigated cooperativity and intramolecular hydrogen bonding in donor–acceptor complexes of phenol, catechol, pyrogallol, and 1,2,3,4-tetrahydroxybenzene with pyridine, trimethylamine, and trimethylphosphine oxide. A positive cooperative effect of a topological intramolecular hydrogen bond in catechol relative to phenol was shown. On the contrary, insignificant or small negative effects were shown for the additional phenol OH groups. Furthermore, cooperativity and topological hydrogen bonding in complexes of three aromatic diols, catechol, naphthalene-1,8-diol, and fluorene-4,5-diol, with a long series of hydrogen bond acceptors that have oxygen, nitrogen, and sulfur acceptor atoms were investigated using DFT at the B3LYP/6-311+G(d,p) level [71]. Cooperativity was found to be weak for catechol and strong for the other two diols, and increased with the hydrogen bond basicity of the acceptor.

**Figure 9:**
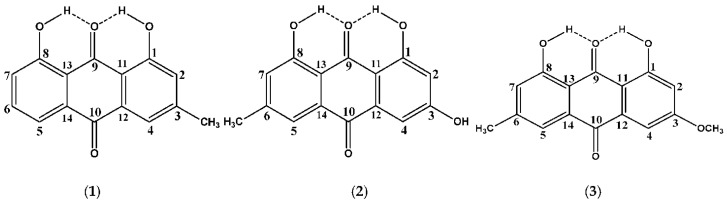
Chemical structures of chrysophanol (**1**), emodin (**2**), and physcion (**3**) [72].

**Figure 10:**
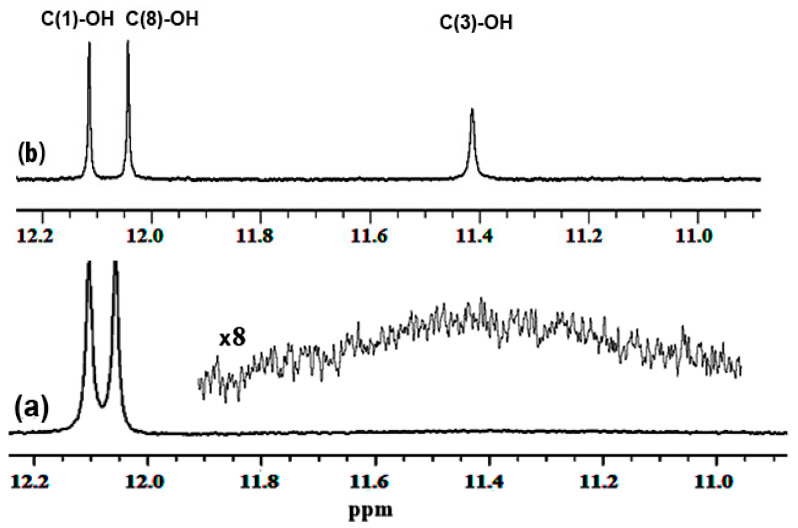
The OH ^1^H NMR spectral region (400 MHz) of emodin (**2**) in DMSO-d_6_ before the addition (**a**) and after the addition of 2 μL of TFA (**b**) [72].

Mari et al. [72] investigated solvent-dependent structures of the natural products chrysophanol, **1**, emodin, **2**, and physcion, **3** (Figure 9), which belong to the anthraquinone family with a variety of biological properties [73,74,75]. The ^1^H NMR spectra of the three compounds showed very deshielded resonances at ~ 12 ppm (Table 3), which can be attributed to the C(1)-OH and C(8)-OH protons, which participate in a strong intramolecular hydrogen bond with the O=C(9) group. Contrary to the case of C(1)-OH and C(8)-OH groups, which show very sharp resonances with chemical shifts that are not affected by the solvent, the C(3)-OH resonance of emodin (2) was found to be extremely broad (Figure 10). The addition of a minor amount of trifluoroacetic acid (TFA) resulted in very sharp resonances and the accurate determination of the chemical shifts in DMSO-d_6_ (δ = 11.41 ppm), acetone-d_6_ (δ = 10.21 ppm), and CDCl_3_ (δ = 6.18 ppm).

**Figure 11:**
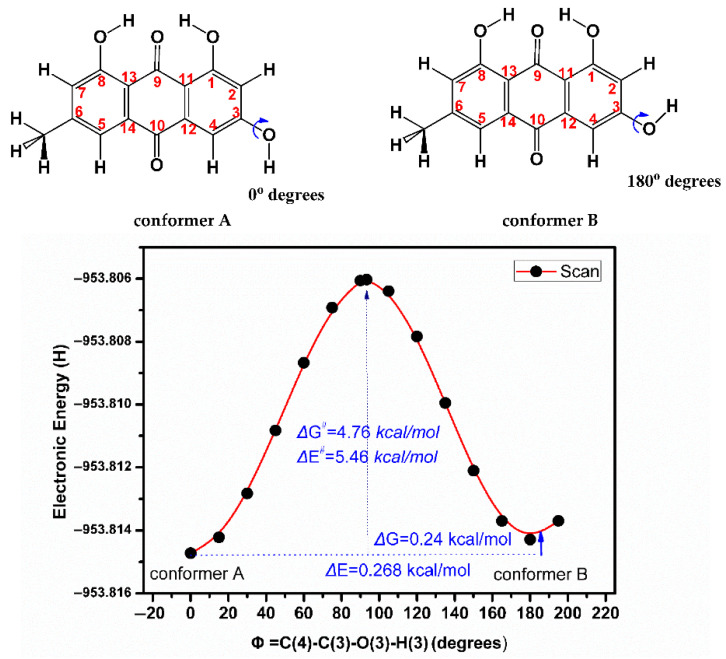
The electronic energy (Hartree unit) of emodin (**2**), as a function of the torsion angle φ: C(4)–C(3)–O(3)–H(3). All calculations were carried out in steps of 15° at the B3LYP/6-31 + G(d) level (gas phase), and the NMR calculations with GIAO at the B3LYP/6-311G + (2d,p) level with continuum (CPCM-CHCl_3_) [72].

**Figure 12:**
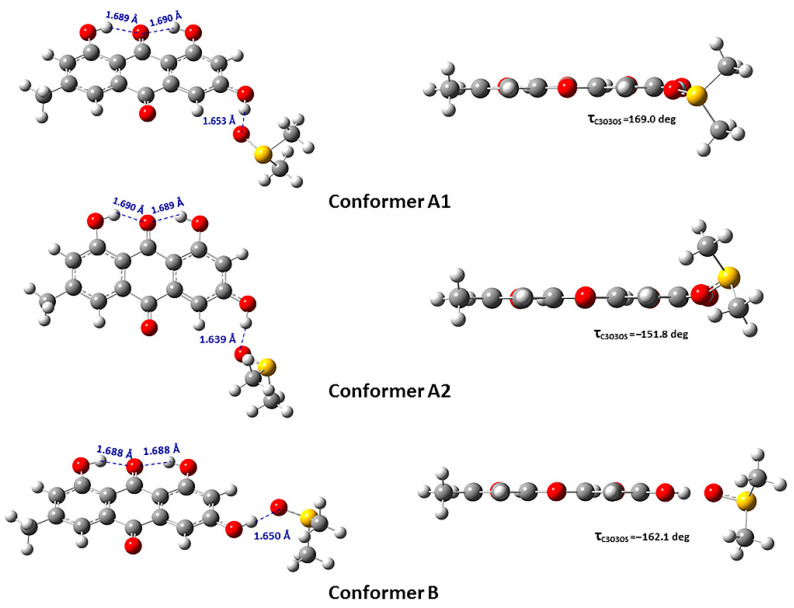
Different perspectives of the B3LYP/6-31 + G(d) (CPCM-DMSO) optimized structures of the three conformers A_1_, A_2_, and B of emodin (**2**) with a discrete molecule of DMSO [72].

The electronic energy (Hartree units) of emodin (**2**) as a function of the torsion angle φ = C(4)-C(3)-O(3)-H(3) showed a maximum at φ = 90° and two minima at φ = 0 and φ = 180° (Figure 11). Their Gibbs free energy difference was found to be very small with ΔG = 0.24 to 0.11 kcal/mol for various functionals and basis sets [72]. Three low-energy conformers in the emodin (**2**) + DMSO complex of CPCM-DMSO were obtained (Figure 12) with strong intermolecular hydrogen bonds O-H···O(S) of 1.639 Å (conformer A_1_), 1.653 Å (conformer A_2_), and 1.650 Å (conformer B). In conformers A_1_ and B, the two CH_3_- groups of DMSO were found to be perpendicular and symmetrical to the planar aromatic system. The torsion angles O-H···O(S) were found to be 125.01°, 169.37°, and 178.14° for conformers A_1_, A_2_, and B, respectively.

**Figure 13:**
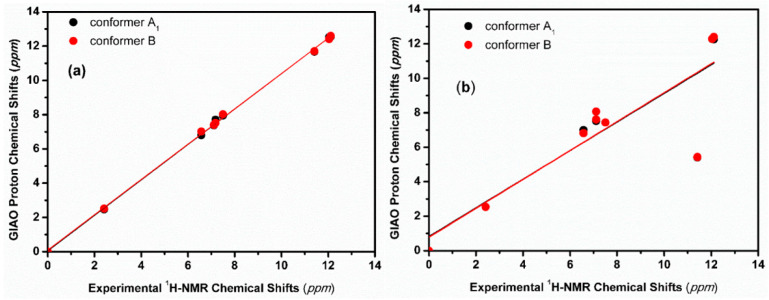
Calculated at the B3LYP/6-311+G(2d,p) level vs. experimental ^1^H NMR chemical shifts in conformers A_1_ and B of emodin (**2**): (**a**) with a discrete molecule of DMSO at the C(3)–OH group in continuum (CPCM-DMSO) and (**b**) in continuum (CPCM-DMSO) [72].

**Figure 14:**
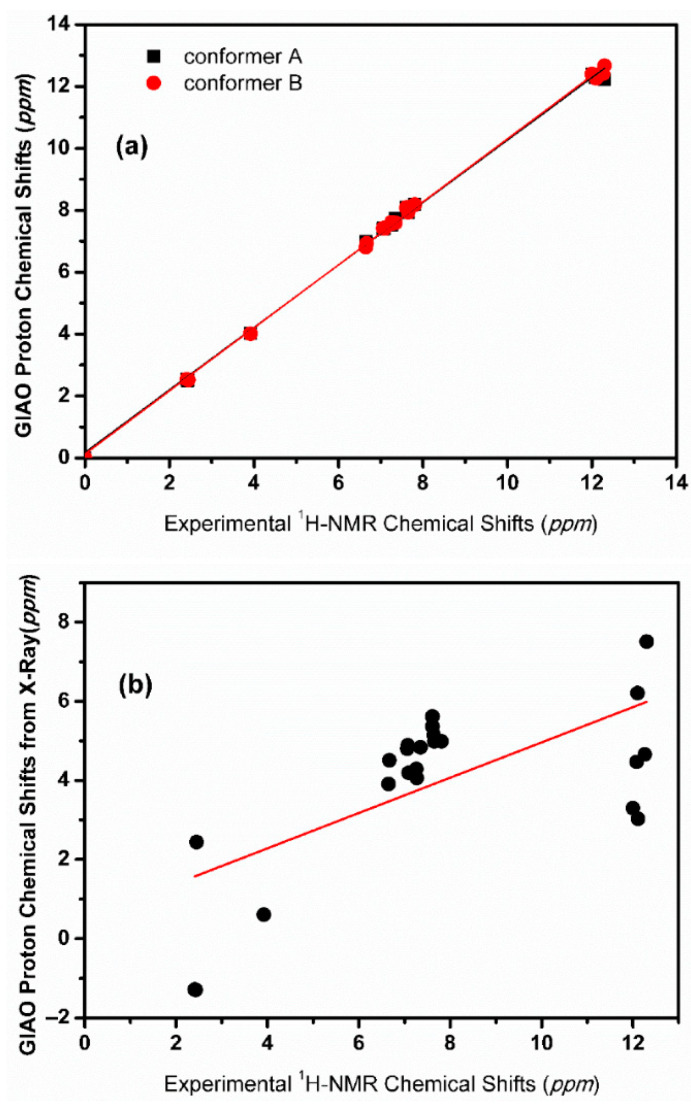
Calculated (δ_calc,_ ppm) at the B3LYP/6-311+G(2d,p) level vs. experimental (δ_exp,_ ppm) values of ^1^H NMR chemical shifts in chrysophanol (**1**), emodin (**2**), and physcion (**3**) (Figure 9): (**a**) with optimized structures having a discrete molecule of CHCl_3_ (CPCM-CHCl_3_) at the B3LYP/6-31+G(d) level and (**b**) by using X-ray structures [76,77,78] as input geometries (δ_calc, x-ray_) at the same level of theory (CPCM-CHCl_3_) [72].

The inclusion of a single molecule of DMSO and acetone in the C(3)-OH group of emodin (**2**) results in the excellent agreement of δ_exp_ and δ_calc_ ^1^H NMR chemical shifts with R^2^ = 0.9991 and 0.9992, slopes of 1.0347 and 1.0305, and mean square errors of 0.0909 and 0.0882 ppm for conformers A_1_ and B, respectively (Figure 13) [72]. On the contrary, the use only of the continuum CPCM-DMSO results in poor linear regression analysis data of R^2^ = 0.7181 and 0.7822, slopes of 0.8309 and 0.8384, and mean square errors of 1.5026 and 1.5057 for A_1_ and B conformers, respectively, due to significant differences in the C(3)-OH chemical shifts (Figure 13b). When the single crystal X-ray structures of chrysophanol (1) [76], emodin (2) [77], and physion (3) [78] were used as input geometries, the DFT calculated ^1^H NMR chemical shifts strongly deviated from the experimental values (Figure 14b). It was concluded that δ_calc_ of ^1^H NMR chemical shifts could provide more reliable results for hydrogen bond and C-H bond lengths than those obtained from single crystal X-ray data [62,63,72,79,80].

**Figure 15:**
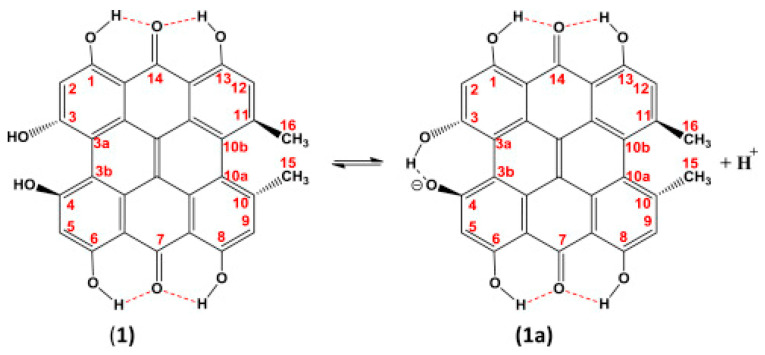
The solvent-dependent equilibrium of the 7,14-dioxo tautomer of hypericin HyH, Q7, 14 (**1**), and its anionic form Hy^−^ (**1a**). Reproduced with permission from [61]. Copyright 2016, Elsevier, Inc., Amsterdam, The Netherlands.

**Figure 16:**
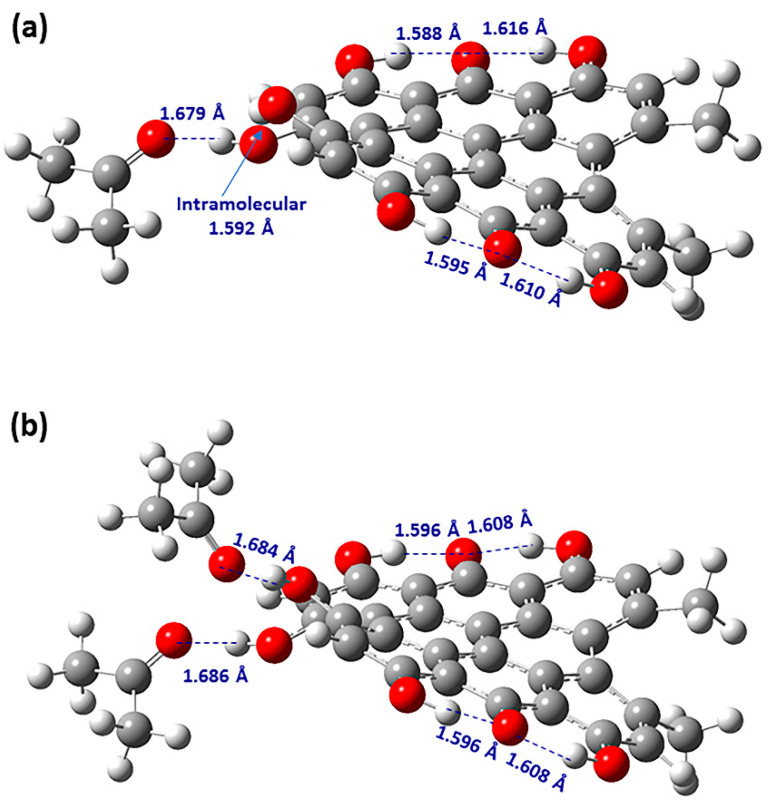
(**a**) The structure of the 7,14-dioxo tautomer of hypericin HyH, Q7, 14, + acetone 1:1 solvation complex, optimized at the TPSSh/TZVP CPCM level of theory. (**b**) The structure of the 7,14-dioxo tautomer of hypericin HyH, Q7, 14, + acetone 1:2 solvation complex, optimized at the TPSSh/TZVP CPCM level of theory. Reproduced with permission from [61]. Copyright 2016, Elsevier, Inc., Amsterdam, The Netherlands.

Siskos et al. [61] investigated chemical shift assignment and effects of solvents on the structure, conformation and ionic state of hypericin, which is one of the major biologically active constituents of *Hypericum perforatum* (Saint John’s wort) [81,82]. The 7,14 tautomer with the carbonyl groups in positions C-7 and C-14 (Figure 15) is the exclusive tautomer in acetone and DMSO. However, in DMSO solution, hypericin exists in the ionic form, due to the deprotonation of one of the bay C3-OH and C4-OH groups, which results in the formation of a strong intramolecular ionic hydrogen bond at δ ~ 17.5−18.5 ppm [81,83]. The energy minimized structures of hypericin + acetone (1:1) and hypericin + acetone (1:2) discrete hydrogen bond complexes showed a pronounced propeller conformation of the aromatic skeleton with dihedral angles C(3)-C(3a)-C(3b)-C(4) and C(10)-C(10a)-C(10b)-C(11) at 24.6°−26.3° and 33.2°–33.5°, respectively, at the B3LYP/6-31+G(d), TPSSh/TZVP and CAM-B3LYP levels of theory in the gas phase or with the CPCM/IEF–PCM models (Figure 16). In the hypericin +acetone (1:1) complex, an intermolecular solute–solvent hydrogen bond of 1.679 Å and an intramolecular 3-OH···OH-4 hydrogen bond of 1.592 Å were obtained. In the hypericin +acetone (1:2) complex, two intermolecular solute–solvent hydrogen bond interactions for 1.686 Å and 1.684 Å were obtained (Figure 16).

**Figure 17:**
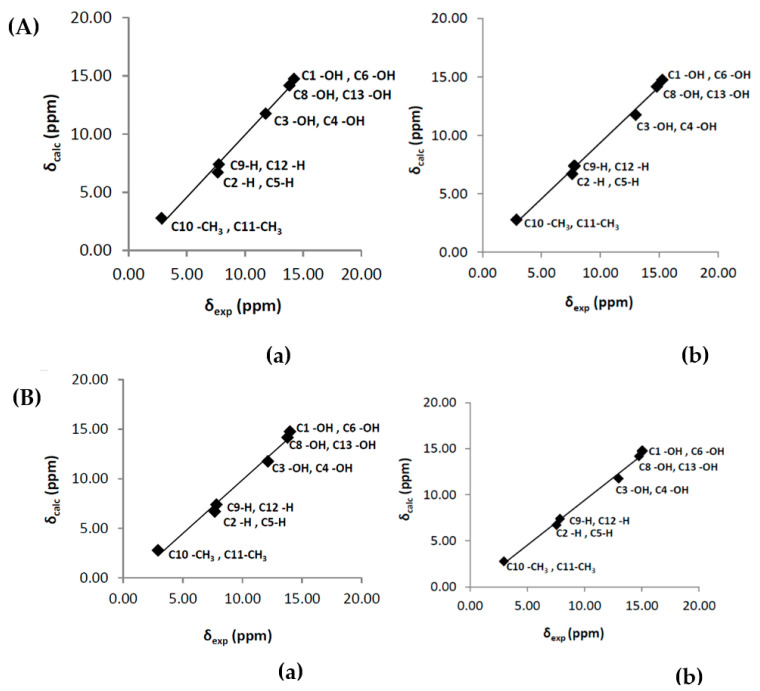
(**A**) Calculated (δ_calc_, ppm) at the B3LYP/6-311 + G(2d,p) level of theory vs. experimental values (δ_exp_, ppm) ^1^H NMR chemical shifts in neutral hypericin + 1 molecule of acetone with minimization of the structures at the B3LYP/6-311 + G(d) (CPCM) (**a**) and at the TPSSh/TZVP (CPCM) (**b**) levels of theory, respectively. (**B**) Calculated (δ_calc_, ppm) at the B3LYP/6-311 + G(2d,p) level of theory vs. experimental values (δ_exp_, ppm) ^1^H NMR chemical shifts in neutral hypericin + 2 molecules of acetone with minimization of the structures at the B3LYP/6-31 + G(d) (CPCM) (**a**) and at the TPSSh/TZVP (CPCM) (**b**) levels of theory, respectively. Reproduced with permission from [61]. Copyright 2016, Elsevier, Inc., Amsterdam, The Netherlands.

**Figure 18:**
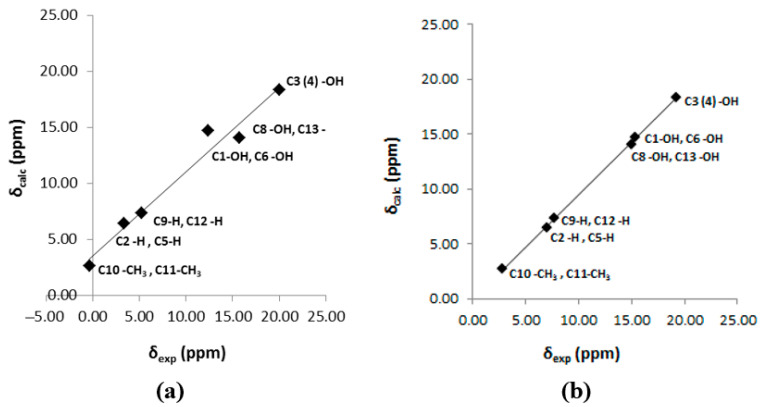
Calculated (δ_calc_) at the B3LYP/6-31 + G(2d,p) level of theory vs. experimental values (δ_exp_) of the ^1^H NMR chemical shifts in hypericinate with the use of the X-ray structure [85] (**a**) and minimization of the structure at the TPSSh/TZVP IEF-PCM level of theory (**b**). Reproduced with permission from [61]. Copyright 2016, Elsevier, Inc., Amsterdam, The Netherlands.

The calculated δ_calc_ vs. experimental δ_exp_ of the ^1^H NMR chemical shifts in the neutral hypericin + 1 molecule of acetone and hypericin + 2 molecules of acetone showed very satisfactory agreement with the experimental data, especially in the former case, when using δ = 11.6–11.8 ppm for the OH-3,4 protons, as shown by Skalkos et al. [83] (Figure 17, Table 4). On the contrary, the value from Smirnov et al. [84], δ = 8.2 ppm, strongly deviated from linearity. When the X-ray structure was used as an input geometry [85], the agreement among the experimental data was reduced significantly (R^2^ = 0.9678, intercept = 0.7472, and mean square error = −1.44 ppm (Figure 18)). It was concluded that for accurate structural data on hydrogen bond distances, especially of those of a cooperative nature, more demanding basis sets, e.g., TPSSh/TZVP (CPCM) for energy minimization of the structures, may be required.

**Figure 19:**
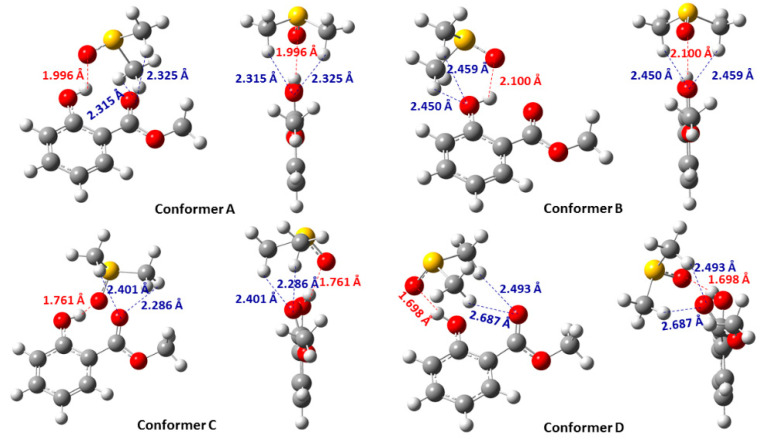
The structures of the low-energy conformers in methyl salicylate + DMSO (1:1) solvation complexes (A–D) optimized at the ωB97XD/631G+d level. Reproduced with permission from [64]. Copyright 2018, Elsevier, Inc., Amsterdam, The Netherlands.

Siskos et al. [64] performed detailed DFT structural and conformational analysis, including discrete solute–solvent interactions of methyl 2,5-dihydroxybenzoate and methyl salicylate, which is an organic ester produced by several plant species (Figure 19). Excellent linear correlation between δ_comp_(^1^H) and δ_exp_(^1^H) was obtained using moderate basis sets for energy minimization. Surprisingly, more advanced dispersion correlated functionals, such as APFD/6-31+G(d) and APFD/6-311+G(2d,p), performed less well than the B3LYP functional. It was concluded, in agreement with previous studies [60,61,62,63], that the great sensitivity of the phenolic OH ^1^H chemical shifts to torsion angle effects, hydrogen bonding, and solute–solvent interactions can provide an excellent method for obtaining high-resolution structures in solution which are more precise than those obtained by the crystalline sponge [86,87] and single-crystal X-ray methods.

Figure 19 shows the DFT results of methyl salicylate, using a discrete solvent molecule of DMSO, with energy minimization at the ωB97XD/6-31+G(d) level. In the lower Gibbs energy conformers A and B, the planar intramolecular hydrogen bond of the phenol OH group and the C=O group of the ester persists, despite the high solvation and hydrogen bond ability of DMSO. The OH and S=O groups are in plane with the aromatic ring, while the two methyl groups of DMSO are asymmetrical to this plane. The computed ^1^H NMR chemical shift in the phenolic OH group of the most stable conformer A (δ = 10.35 ppm) shows an excellent agreement with the experimental value, δ = 10.49 ppm (Table 5). In the conformer C, the OH and S=O groups are not in plane with the aromatic ring. In the conformer D, the intramolecular hydrogen bond is broken, and an intermolecular hydrogen bond is formed between the phenolic OH and the S=O bond of DMSO. The calculated ^1^H NMR chemical shifts in the various low-energy conformers (A–D), weighted by the respective Boltzmann populations, resulted in excellent agreement with the experimental values (δ_calc_(OH) = 10.35 ppm, δ_exp_(OH) = 10.49 ppm).

### 2.2. Alcohols

Lomas [88] investigated experimental and DFT calculated chemical shifts and vicinal coupling constants of butane-1.4-diol, **1**, pentane-1,4-diol, **2**, (S,S)-hexane-2,5-diol, **3**, 2,5-dimethylhexane-2,5-diol, **4**, and cyclohexane-1,4-diols (cis/trans), **5**,**6**, in benzene, chloroform, and DMSO (Table 6). Very significant chemical shift differences in the OH groups in DMSO-d_6_ and C_6_D_6_ were observed in the range of 3.83 to 3.28 ppm. DFT computational Gibbs free energies of the various conformers were calculated using the IEFPCM model, at the PBE0/6-311+G(d,p) level, and NMR chemical shifts with the GIAO method at the PBE0/6-311+G(d,p) geometry, using the cc-PVTZ basis set. The computed chemical shifts in the CH protons correlate with the experimental values with a gradient of 1.07 ± 0.01; the OH protons, however, correlate less satisfactorily. The inclusion of discrete solvent molecules was considered to be a very difficult task due to the large number of conformational states involved.

**Figure 20:**
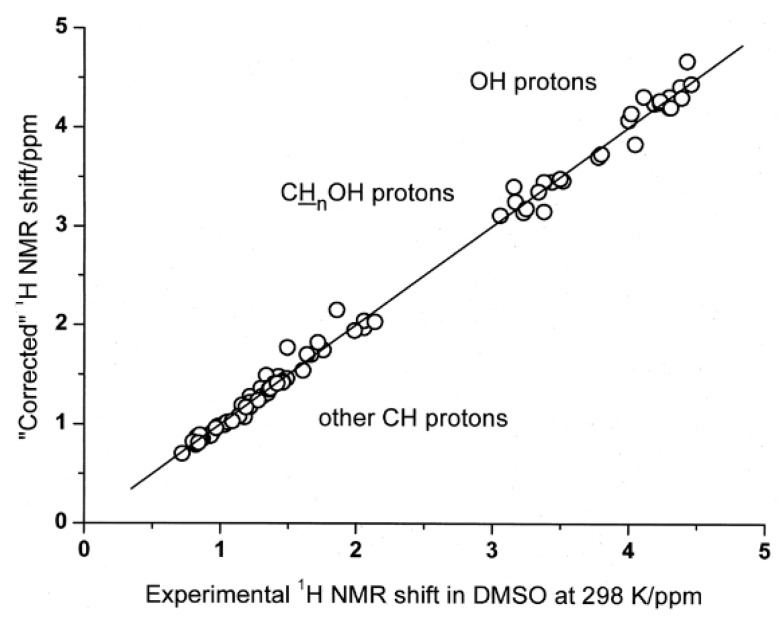
Correlation of ‘corrected’ ^1^H NMR shifts against experimental values in DMSO at 298 K (gradient = 1.00 ± 0.01, intercept = 0.00 ± 0.02 ppm, and R^2^ = 0.09 ppm). Reproduced with permission from [89]. Copyright 2016, Wiley, Chichester, West Sussex, UK.

Lomas [89] investigated experimental (including data from the literature) and DFT calculated ^1^H NMR chemical shifts in a variety of saturated alcohols in benzene, chloroform, DMSO and pyridine. The OH chemical shifts and hydrogen bond distances of methanol or ethanol in complexation with pyridine strongly depend on the functional used and very little on the basis set. The CH proton chemical shifts are insensitive to both the function and basis set used and do not reflect the nature of the solvent either in the complex or in the continuum. The chemical shifts in OH, CHOH, and other CH groups are systematically overestimated by 0.42, 0.21, and 0.06 ppm, respectively, for the corresponding complexes in the gas phase. When the computed chemical shifts were corrected accordingly, a very good correlation was obtained (Figure 20). For acetone and acetonitrile, which are weaker hydrogen bond acceptors than DMSO, the computational chemical shift in the OH groups deviated significantly from the experimental data (Table 7). This was attributed to the presence of a fraction of free alcohol molecules, even in dilute solution, in acetone and acetonitrile. The situation, therefore, of alcohols appears to be more complex than in phenols, which are significantly more acidic and, thus, stronger hydrogen bond acceptors [89].

DFT studies of ^1^H NMR chemical shifts and atom–atom interaction energies in alkane-1,2- and 1,3-polyols and their pyridine complexes were reported [90]. No evidence of cooperativity was found for O–H···OH hydrogen bonding. DFT studies of ^1^H NMR chemical shifts, interatomic distances, atom–atom interaction energies, and atomic charges of intramolecular O–H···O and C–H···O hydrogen bond cooperativity were reported for D-glucopyranose and D-galactopyranose [91]. Complexation with pyridine resulted in an increase in the interaction energy of the N···H1–O hydrogen bond due to the presence of successive OH groups.

**Figure 21:**
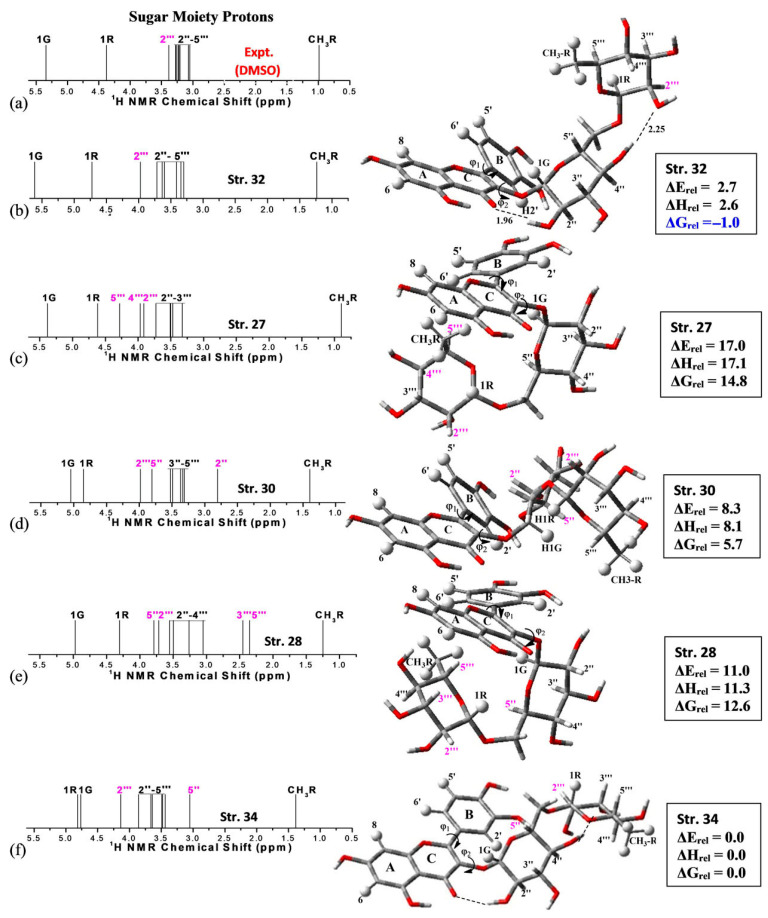
B3LYP/6-31G(d,p) optimized structures (φ_1_ and φ_2_ rotated) for the preferred sugar moieties conformations of rutin in DMSO solution, along with B3LYP/6-31G(d,p) relative energy values (in kcal mol^−1^). Experimental (in DMSO-d_6_) from [94] (**a**) and B3LYP/6-31G(d,p)-PCM-DMSO (**b**–**f**) ^1^H NMR schematic spectra are also shown. The hydrogen atoms showing larger deviations are highlighted in pink [93].

DFT calculations of ^1^H NMR chemical shifts in L-quebrachitol isomers were performed using the B3LYP functional with 6-31G(d,p) and 6-311+(2d,p) basis sets and the polarizable continuum model [92]. A good agreement between computational and experimental data was reported in D_2_O solution. De Souza et al. [93] performed ^1^H NMR chemical shift DFT calculations of the flavonoid rutin in DMSO-d_6_ using the polarizable continuum model (PCM), with emphasis on sugar moieties. Among 34 energy-minimized structures, structure **32** with the B-ring deviated 30° from a planar configuration showed the best agreement with the experimental literature ^1^H NMR chemical shifts (Figure 21, Table 8). It was concluded that, although the B3LYP and M062X functionals are adequate for ^1^H NMR chemical shift calculations, more extensive analysis using various functionals may be required.

**Figure 22:**
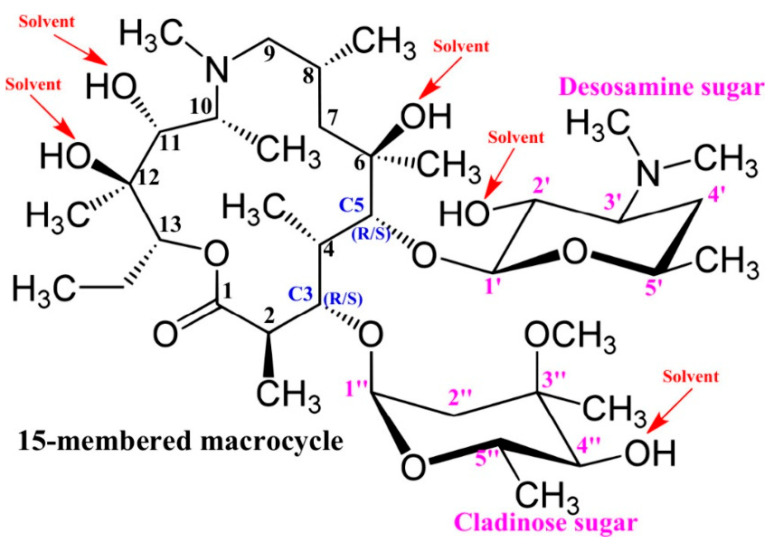
Structural formula of azithromycin. The potential OH groups for interaction with explicit solvent molecules are shown. Reproduced with permission from [95]. Copyright 2025, The American Chemical Society, Washington, DC, USA.

Of particular interest is the recent DFT work of Da Silva et al. [95], who investigated solvent effects on relative energies and NMR chemical shifts in the antibiotic azithromycin (AZM) (Figure 22). Geometry optimization, using four structures previously reported for AZM [96] and the X-ray structure [97], was performed at the ωB97XD/6-31+G(d,p) level using the PCM model, with the explicit solvent molecules PCM-*n*CHCl_3_, PCM-*n*H_2_O, and PCM-*n*DMSO (*n* = 0, 5, 15, and 25). Two molecules of AZM with 50 explicit solvent molecules were also investigated. The use of continuum models, including five solvent molecules that are placed in potential OH groups by chemical intuition [98], was suggested to be the optimum computational strategy. The use of five solvent molecules of CHCl_3_ resulted in similar relative energies and RMSD NMR chemical shifts as in the PCM-*n*CHCl_3_ (*n* = 50) solvation model. In the case of H_2_O and DMSO, increasing the number of explicit solvent molecules (*n* > 5) results in moderate changes in RMSD of computational vs. experimental [99,100,101] chemical shifts; however, the energy profiles may be significantly affected by the solvation model used. This can be attributed to solvent–solvent interactions, which can result in large changes in the total energies of distinct conformers. In addition, it was concluded that the PCM model, without explicit molecules of solvents, is adequate for the calculation of ^13^C NMR chemical shifts, in agreement with previous studies [34,102,103].

### 2.3. Amides and Amines

**Figure 23:**
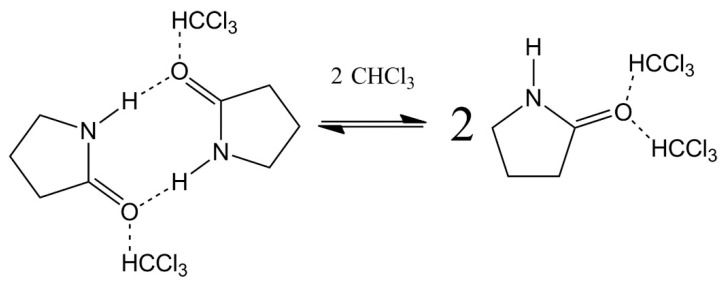
Self-association and solvation of N-methyl-2-pyrrolidinone in chloroform solution. Reproduced with permission from [107]. Copyright 2017, The American Chemical Society, Washington, DC, USA.

Proton chemical shifts in amides, peptides and proteins have been extensively investigated as sensitive probes of structural parameters of hydrogen bonds, and their magnitude has been related to the strength of the hydrogen bond. The chemical shifts in hydrogen bonding NH protons are also sensitive to secondary and tertiary structural effects, and temperature coefficients have been widely used to predict hydrogen bond donors in peptides and proteins [104,105,106]. Amides are the simplest models of peptides and proteins, which can be exploited as models of solute–solvent interactions. Molchanov and Gryff-Keller [107] investigated experimental and computational ^1^H and ^13^C chemical shifts in N-methyl-2-purrolidinone, N,N-dimethylformamide (DMF), 2-pyrrolidinone, *E/Z* N-methyl-formamide, and formamide in chloroform and DMSO (Table 9 and Table 10). DFT calculations of primary and secondary amides, at the B3LYP and PBE0 6-311++G(2d,p) PCM in CHCl_3_, were analyzed in terms of an equilibrium between self-associated and solvated species, with two molecules of CHCl_3_ at the amide oxygen (Figure 23). Self-association of the amides in DMSO is of minor importance, and the NH group is strongly hydrogen-bonded with a single molecule of DMSO.

**Figure 24:**
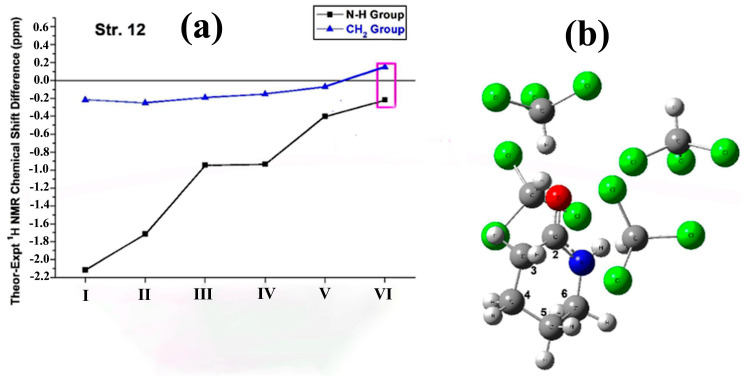
(**a**) ^1^H NMR chemical shift difference (in ppm) between theoretical (B3LYP**-**PCM-CHCl_3_) and experimental (in CDCl_3_) data for valerolactam using a specific number of explicit CHCl_3_ solvent molecules: **I**, in vacuum; **II**, PCM only; **III**, PCM + CHCl_3_; **IV**, PCM + 2CHCl_3_; **V**, PCM + 3CHCl_3_; and **VI**, PCM + 4CHCl_3_. The best fit of the NH and CH_2_ groups is indicated in the pink box. (**b**) Optimum solvated structure [108].

Da Silva and De Almeida [108] performed DFT and MP2 ^1^H NMR chemical shift calculations for a series of amine and amide compounds. The use of PCM does not predict the N-H chemical shifts in solution successfully. The use of a limited number of explicit solvent molecules in DFT-PCM-CHCl_3_ geometry optimizations resulted in good agreement with the experimental NH chemical shift data. Figure 24 shows the computational data of valerolactam, which is a cyclic six-membered amide. The chemical shift difference (ppm) between computational (in vacuum) and experimental data is very significant (~−2.2 ppm, Figure 24a), and it is only slightly improved with the use of the PCM model (~−1.8 ppm). Inclusion of explicit solvation molecules of CHCl_3_ significantly improves the agreement with the experimental data, particularly for the PCM+3CHCl_3_ model. It was emphasized that improving the agreement between computational and experimental data for the N-H proton, there is a decrease in the agreement for the CH_n_ chemical shifts.

### 2.4. Pyrimidine Basis

**Figure 25:**
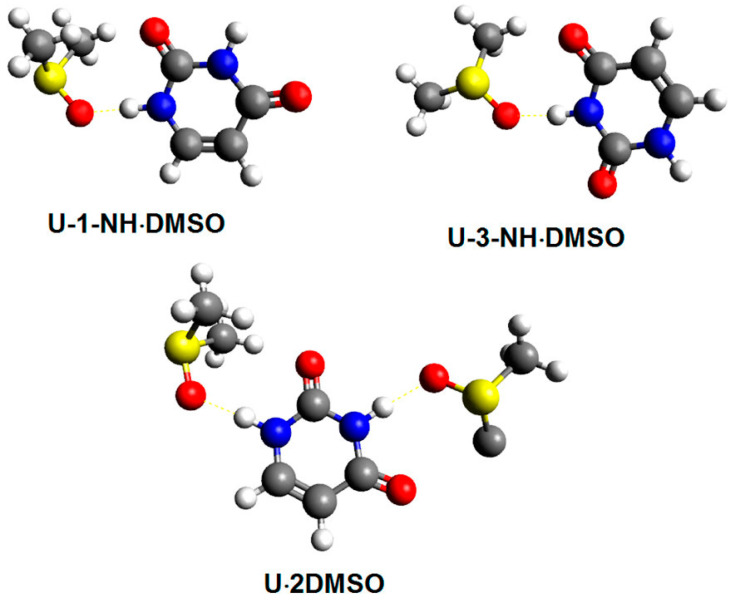
Probable structures of uracil solvates in DMSO solution. Reproduced with permission from [109]. Copyright 2017, The American Chemical Society, Washington, DC, USA.

**Figure 26:**
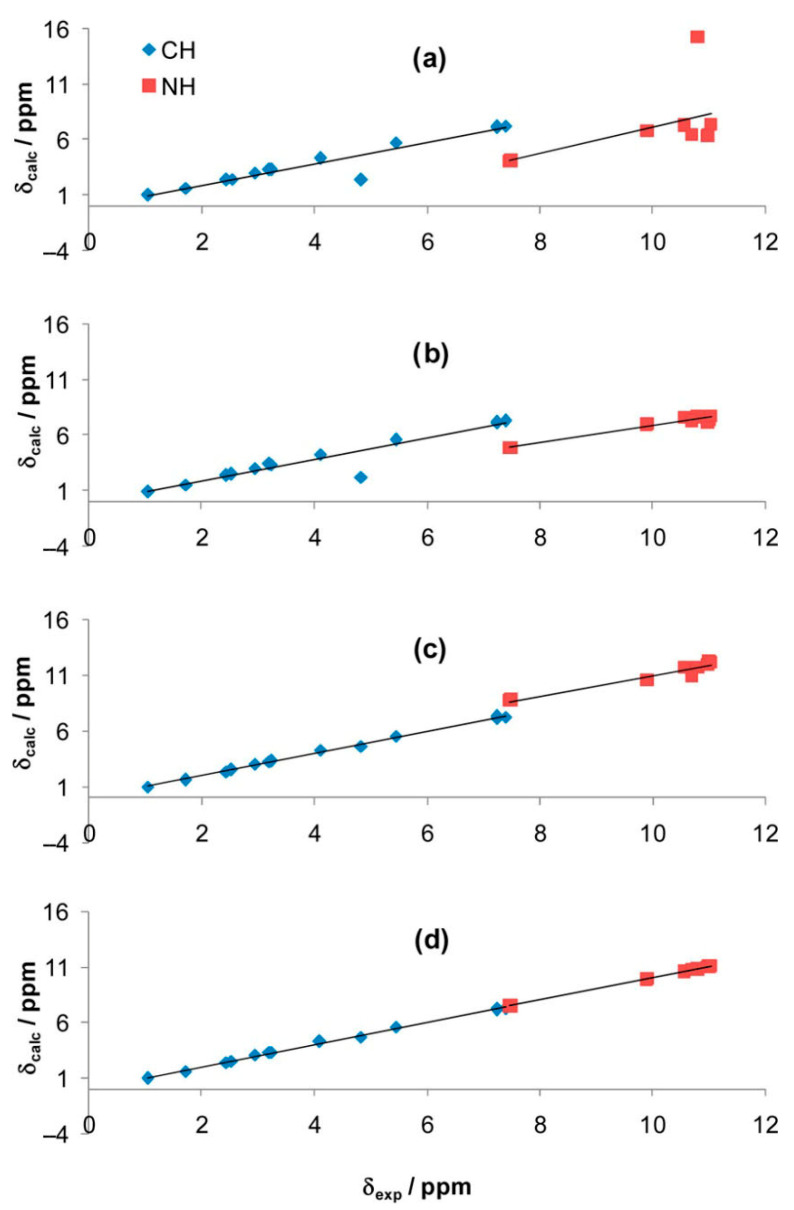
Comparison of ^1^H NMR chemical shifts in uracil (U), thymine (T), 5-hydroxymethyluracil (HMU), 5,6-dihydrouracil (DHU) and 5,6-dihydrothymine (DHT), calculated using the B3LYP/6-311++G(2d,p) method, assuming various solvation models, with the experimental data measured for DMSO solutions: (**a**) gas phase, (**b**) PCM(DMSO) approach, (**c**) PCM(DMSO) applied to disolvates 1,3-NH···DMSO, and (**d**) the PCM(DMSO) approach with incomplete specific solvation of 1-NH and 3-NH hydrogens. In the case of HMU, all solvated species were additionally solvated by a DMSO molecule at the OH group. Reproduced with permission from [109]. Copyright 2017, The American Chemical Society, Washington, DC, USA.

Kubica et al. [109] investigated the solvation of uracil (U), thymine (T), 5-hydroxymethyluracil (HMU), 5,6-dihydrouracil (DHU), and 5,6-dihydrothymine (DHT) in DMSO using experimental and DFT calculated data on ^1^H and ^13^C NMR chemical shifts. In all cases, the solvent molecules of DMSO were bound to the solute with the formation of a single hydrogen bond (Table 11, Figure 25), which is approximately colinear with the N–H bonds (φ(NH···O) > 172°) and the (φ(H···O(S)) ~ 120°. The inclusion of the nonspecific solvation model (PCM) resulted in better agreement with the experimental results, relative to those in the gas phase; remarkably better, however, results were obtained when specific solvation of all the NH groups (and the OH in HMU) was taken into account (Figure 26). Further analysis was also performed with the hypothesis that the specific solvation of N-H groups is incomplete. The analysis included four species: free molecules, and solvates formed by 1-NH, 3-NH, or both NH groups (Table 12). The least-squares analysis of the ^1^H NMR chemical shifts reproduced the experimental results quite satisfactorily (Figure 26).

### 2.5. Carboxylic Groups

**Figure 27:**
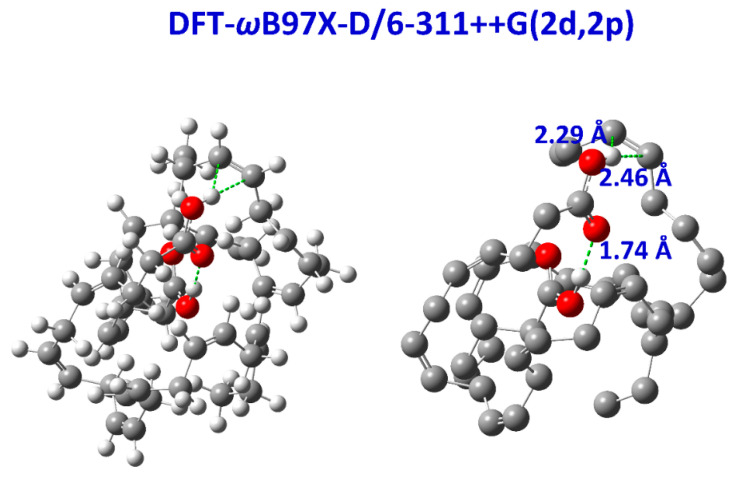
The optimized structure of dimeric DHA with all hydrogens present (**left**) and without hydrogens, except for those participating in hydrogen bonding (**right**). The optimized structure supports the flip–flop hydrogen bonding phenomenon. Reproduced with permission from [111]. Copyright 2021, Elsevier, Inc., Amsterdam, The Netherlands.

Venianakis et al. [110,111] investigated with the use of ^1^H NMR and DFT calculations, low-energy conformers of caproleic acid (CA, 10:1 cis-9), oleic acid (OA, 18:1, cis-9), α-linolenic acid (ALA 18:3 cis-9, 12, 15 and ω-3), eicosapentanoic acid (EPA, 20:5 cis-5, 8, 11, 14, 17 ω-3), and decosahexaenoic acid (DHA, 22:6 cis-4, 7, 10, 13, 16, 12 and ω-3) in the liquid state. The major species are those of strong intermolecular centro-symmetric cyclic hydrogen bond interactions of the carboxylic groups in an equilibrium of parallel and antiparallel interdigitated structures. For DHA, the strong shielding of δ(COOH) ~ 8.5 ppm was interpreted in terms of a flip–flop process [112] between a classical intermolecular centro-symmetric dimeric structure and a novel intermolecular hydrogen bond between the carboxylic group of one molecule and the terminal double bond of a second molecule of DHA (Figure 27).

**Figure 28:**
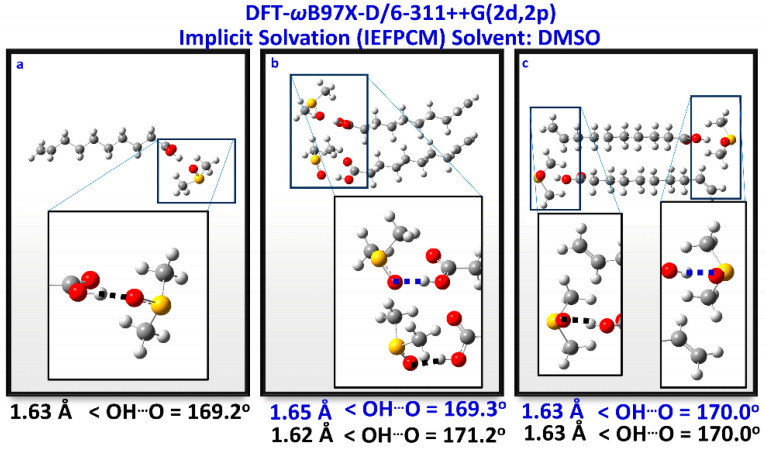
The optimized structures of caproleic acid (CA), with a discrete solvation molecule of DMSO on the carboxylic group: single molecule of CA (**a**), dimeric structures of CA in parallel (**b**), and antiparallel configuration (**c**). Reproduced with permission from [113]. Copyright 2023, Elsevier, Inc., Amsterdam, The Netherlands.

**Figure 29:**
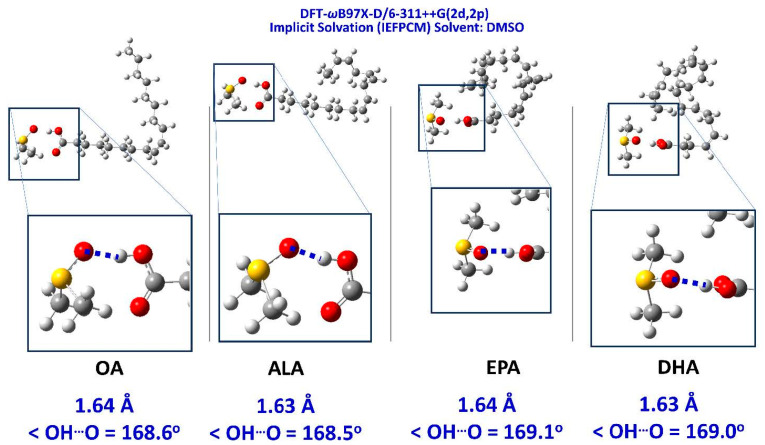
The optimized structures of oleic acid (OA), α-linolenic acid (ALA), EPA, and DHA with a discrete solvation molecule of DMSO in the carboxylic group. Reproduced with permission from [113]. Copyright 2023, Elsevier, Inc., Amsterdam, The Netherlands.

Venianakis et al. [113] reported structural studies of monounsaturated and ω-3 polyunsaturated free fatty acids in CHCl_3_ and DMSO solutions, with the combined use of ^1^H NMR and DFT calculations. Low molecular weight aggregates of dimerized fatty acids through intermolecular hydrogen bond interactions with the carboxylic groups, with parallel and antiparallel interdigitated structures, even at low concentration (20 mM) in CDCl_3_, are the major aggregation species. For the dimeric DHA, a structural model similar to that in the liquid state (Figure 27) was shown to play a role. In DMSO solution, all free fatty acids show a strong hydrogen bond interaction of the carboxylic proton with the oxygen of a single discrete solvation molecule of DMSO (Figure 28 and Figure 29) with OH···O distances of 1.59 to 1.63 Å and bond angles of 169.2 to 171.2°. These hydrogen bonding distances are shorter than those observed in hydrogen bond dimers through the carboxylic groups, with OH···O distances of 1.66 Å. The presence of parallel and antiparallel interdigitated conformers of low molecular weight was also shown in DMSO (C = 20 mM) (Figure 28). The computational shifts in DMSO are higher by ~1.0 to 1.6 ppm than the experimental values (Table 13). This can be attributed to the fact that the ^1^H NMR chemical shifts in carboxylic groups show a strong dependence upon bonding distance and the relative orientation of the DMSO molecule.

**Figure 30:**
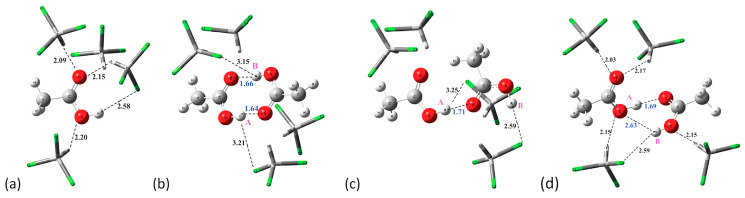
ωB97x-D/6-31G(d,p)-PCM-4CHCl_3_ optimized structures of AA monomer and dimers. The OH groups in the dimer are named A and B for reasons of clarity. (**a**) AA-monomer PCM-4CHCl_3_, (**b**) cyclic dimer PCM-4CHCl_3_, (**c**) open dimer PCM-4CHCl_3_, and (**d**) quasicyclic dimer PCM- 4CHCl_3_. Solute–solute and solute–solvent intermolecular distances (Å) are indicated by a dotted line. Reproduced with permission from [114]. Copyright 2025, Wiley, Chichester, West Sussex, UK.

**Figure 31:**
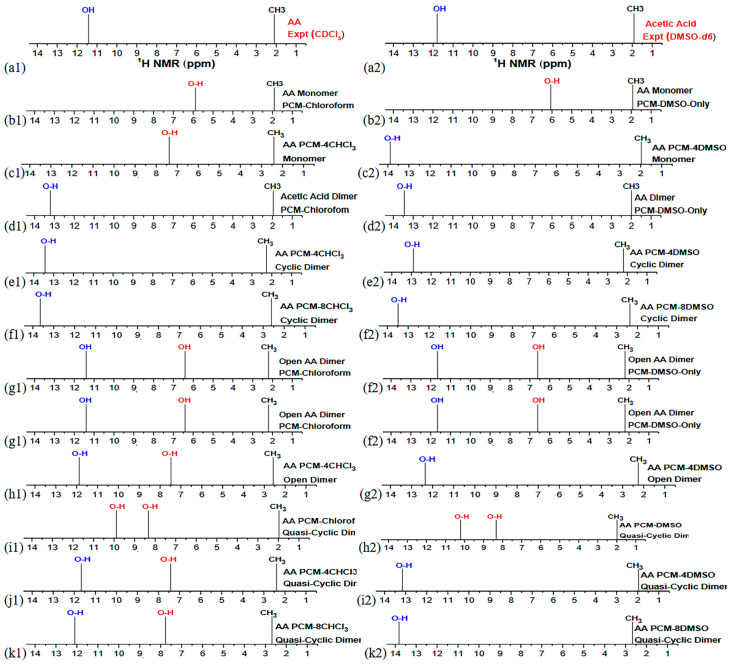
Schematic experimental and computational ^1^H NMR spectra for acetic acid (AA) in CDCl_3_ (**a1**–**k1**) and DMSO-d_6_ (**a2**–**k2**) at the B3LYP/6-31G(d,p) (PCM-*n*CHCl_3_ and PCM-*n*DMSO, *n* = 0, 2, 4, 8) level of theory for AA monomer and dimers (cyclic, open, and quasicyclic). Reproduced with permission from [114]. Copyright 2025, Wiley, Chichester, West Sussex, UK.

Da Silva et al. [114] reported a DFT-PCM study on acetic acid (AA) and 3-indolacetic acid (3-IAA) dimer formation in chloroform and DMSO solution. Several hydrogen bonding solvation species were investigated, including explicit molecules PCM-*n* CHCl_3_ and PCM-*n*DMSO (*n* = 0, 2, 8, 14, 25 and 25). Figure 30 shows optimized structures of acetic acid (AA) monomer and dimers in PCM with four (4) explicit solvation molecules of CHCl_3_: (**a**) monomer, (**b**) cyclic, (**c**) open dimer, and (**d**) quasi cyclic dimer. Figure 31 shows schematic experimental and computational ^1^H NMR spectra in chloroform (Figure 31, a1–k1) and DMSO (a2–k2). The computational chemical shifts in the monomeric acetic acid in PCM strongly deviate from the experimental value. Incorporation of four (4) explicit CHCl_3_ does not significantly improve agreement with the experimental value. The acetic acid dimer, through a classical intermolecular centro-symmetric hydrogen bonding [115,116] in PCM–chloroform, provides significantly better agreement with the experimental data. Incorporation of explicit molecules of chloroform (*n* = 2, 4, 8) increases deviation from the experimental data (Figure 31e1,f1). Very probably, the acetic acid dimer is in equilibrium with a relatively minor population of linear dimers and cyclic and linear trimers [115,116]. The computational chemical shift in acetic acid monomer in PCM-DMSO deviates strongly from the experimental value (Figure 31a2). The chemical shift in acetic acid monomer with four (4) explicit molecules of DMSO deviates by ~ 2 ppm from the experimental value. The authors, furthermore, considered several structural motifs, such as cyclic dimer, open dimer, and quasicyclic dimer (Figure 31d2–k2).

### 2.6. Enol–Enol Tautomerism

**Figure 32:**
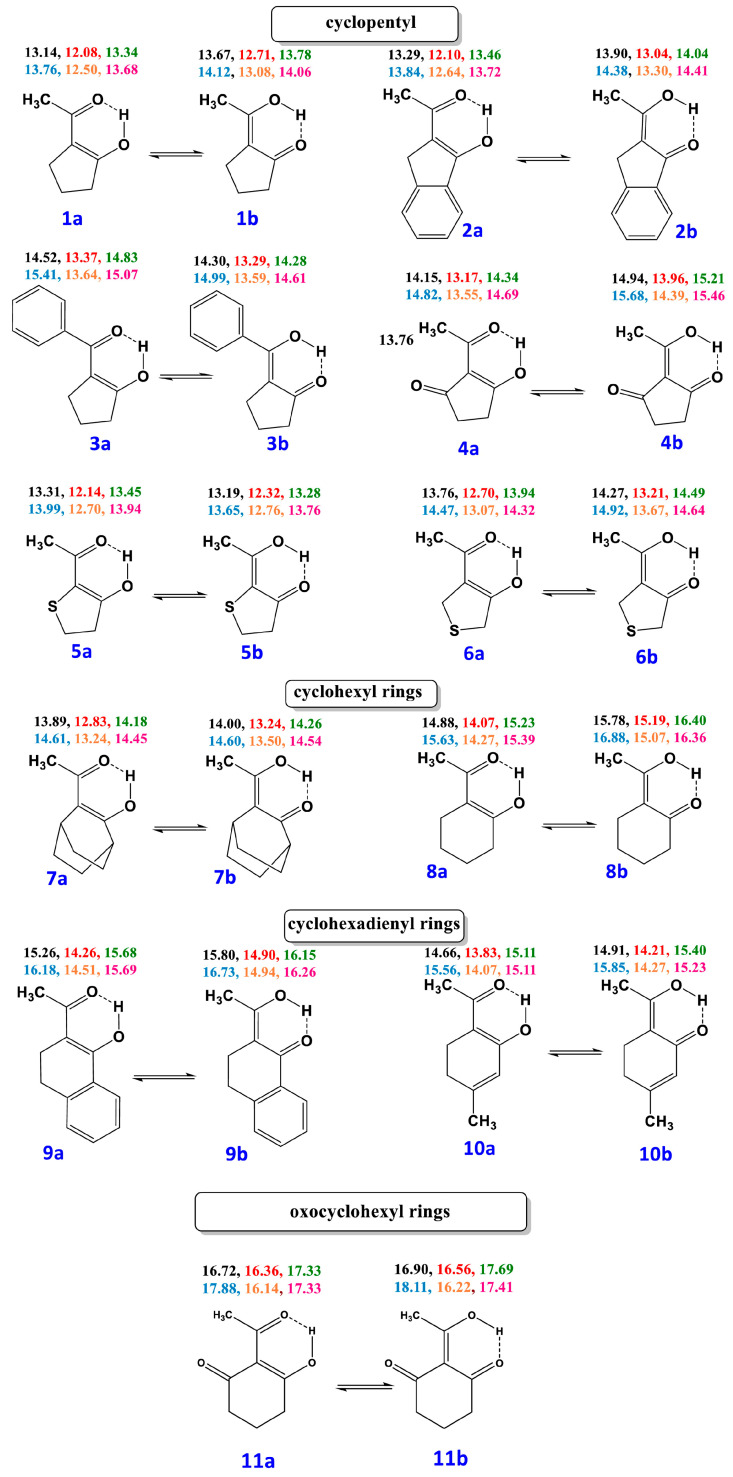
Chemical structures and computed ^1^H OH NMR chemical shifts, ppm, of enol–enol tautomers of the compounds **1**–**11** with energy minimization using the B3LYP/6-31+G(d) (black), M06-2X/6-31+G(d) (red), B3LYP/6-311G(d,p) (green), B3LYP/ def2TZVP (blue), *ω*B97XD/6-31+G(d) (orange) and APFD/6-31+G(d) (purple) methods. Reproduced with permission from [120]. Copyright 2020, Elsevier, Inc., Amsterdam, The Netherlands.

**Figure 33:**
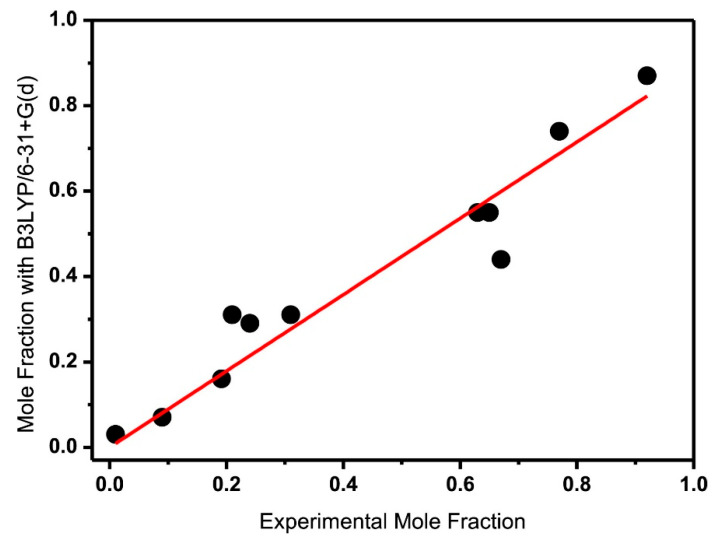
Correlation between the experimental literature mole fractions of enol–enol tautomers, and computational mole fractions at the B3LYP/6-31 + G(d) level. Reproduced with permission from [120]. Copyright 2020, Elsevier, Inc., Amsterdam, The Netherlands.

Enol–enol tautomerism is a classical subject in physical organic chemistry, which, due to the fact that it is mainly intramolecular in origin, has very low activation energy. The interconversion rate, therefore, of various enol tautomers is very rapid on the NMR time scale [117,118,119]. Gibbs energies, ΔG, of equilibrium constants have been estimated indirectly by experimental interpolation methods. Siskos et al. [120] reported a new approach for investigating the impacts of intramolecular hydrogen bonding vs. stereoelectronic factors on enol–enol tautomerism. DFT calculations of O–H···O ^1^H NMR chemical shifts in enol pairs (Figure 32) weighted by their populations, as predicted by the computed ΔG energies, agree well with the experimental chemical shifts and the literature experimentally determined mole fractions (Figure 33). Energy minimization was performed using a variety of functionals and basis sets with the GIAO method at the B3LYP/6-311+G(2d,p) level of theory with the CPCM (CHCl_3_). It would be of interest to investigate, using the above method, the impact of explicit molecules on enol–enol tautomerism.

### 2.7. Mixed Solvents

Mixed H_2_O/organic solvents have been widely utilized to reduce the exchange rate of OH protons at low temperatures and, thus, to obtain accurate chemical shift and J-coupling data [121]. For H_2_O/DMSO mixtures with mole fraction *χ*(DMSO) ~ 0.33, a very low freezing point has been observed (−140 °C), which has been widely used to protect several types of cells from damage during freezing and thawing. Nevertheless, the molecular basis of this phenomenon is not sufficiently understood [122].

**Figure 34:**
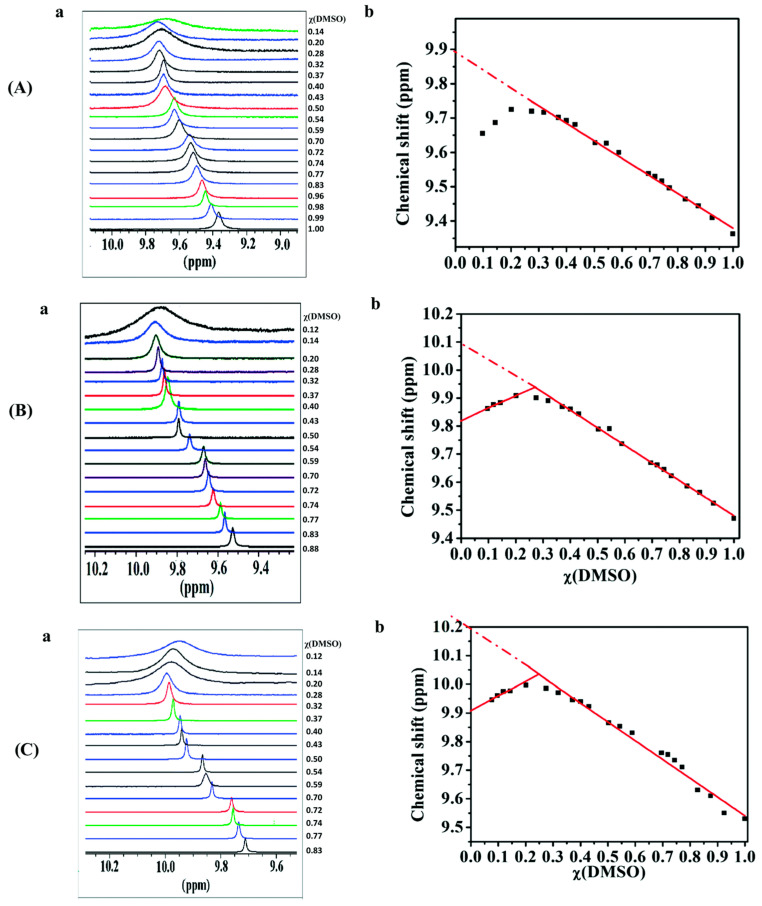
The selected ^1^H NMR spectra of the OH resonance (**a**), and δ(OH) (**b**) of phenol, as a function of the mole fraction, *χ*(DMSO) of DMSO/H_2_O mixtures at 300 K (**A**), 280 K (**B**), and 270 K (**C**). The chemical shift intercepts, δ_interc_, of the linear correlations for *χ*(DMSO) ≥ 0.35 and the chemical shift differences Δδ = δ_interc_ − δ(*χ*(DMSO) = 1) are the following: T = 300 K, δ_interc_ = 9.89 ppm, Δδ = 0.52 ppm; T = 280 K, δ_interc_ = 10.08 ppm, Δδ = 0.63 ppm; and T = 270 K, δ_interc_ = 10.18 ppm, Δδ = 0.65 ppm. For *χ*(DMSO) > 0.88 in (**B**) (**b**) and *χ*(DMSO) > 0.83 in (**C**) (**b**), the solutions were frozen, therefore, OH chemical shifts were obtained through extrapolation of the linear dependence of δ(^1^H) vs. T in the temperature range of 292–318 K. Reproduced with permission from [123]. Copyright 2021, The Royal Society of Chemistry, London, UK.

**Figure 35:**
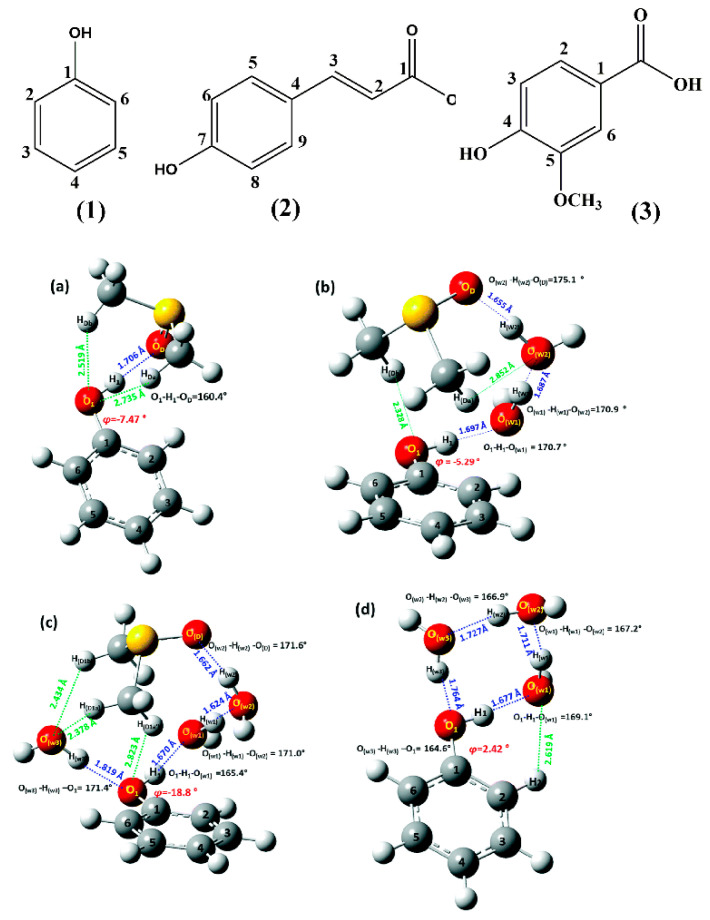
The structural similarities of the DMSO + 3D_2_O complex motif and the neutron powder diffraction data (**a**) [125] with the minimum energy structures of phenol, Ph(**1**), + 2H_2_O + DMSO (**b**), paracoumaric acid, PCA(**2**), + 2H_2_O + DMSO (conformer D) (**c**) and vanillic acid, VA(**3**), + 2H_2_O + DMSO (**d**), with energy minimization using the B3LYP/6-31+G(d) method in the gas phase. Reproduced with permission from [123]. Copyright 2021, The Royal Society of Chemistry, London, UK.

Fatima et al. [123] investigated, with the use of ^1^H NMR and DFT calculations, the molecular basis of H_2_O/DMSO eutectic mixtures using phenol, paracoumaric acid, and vanillic acid as molecular sensors. The experimental chemical shifts in the phenol OH groups, δ_exp_(OH), showed a progressive deshielding upon increasing the concentration of H_2_O. A linear correlation of δ_exp_(OH) vs. *χ*(DMSO) was observed for *χ*(DMSO) ≥ 0.35 (Figure 34). In the range of mole fractions 0.2 ≤ *χ*(DMSO) ≤ 0.33, a broad maximum of δ_exp_(OH) and, thus, in the strength of hydrogen bonding was observed. For *χ*(DMSO) < 0.2, a progressive shielding of δ_exp_(OH) was observed, which was attributed to a decrease in the strength of hydrogen bonding, despite the increase in the number of available acceptor and donor hydrogen bonding sites. DFT calculations were performed using two functionals, B3LYP and APFD, with the G-31+G(d) basis set. The optimized solvation structures were obtained starting with the sensor phenol compound and adding first a specific number of H_2_O molecules, followed by DMSO in various possible sites according to chemical intuition. The ^1^H chemical shifts were calculated with the GIAO and CSGT methods [124] at the B3LYP/6-311+G(2d,p) level in the gas phase (the CPCM method was not used since there is no appropriate continuum model for mixed solvents). The DFT calculated δ_calc_(OH) of minimum energy clusters were shown to be in good agreement with the experimental δ_exp_(OH) data. The structural details of the phenol compounds + 2H_2_O + DMSO complexes were found to be in excellent agreement with a simultaneous publication of a low-temperature neutron diffraction experiment using the DMSO + 3D_2_O complex [125] (Figure 35).

### 2.8. Ionic Compounds

**Figure 36:**
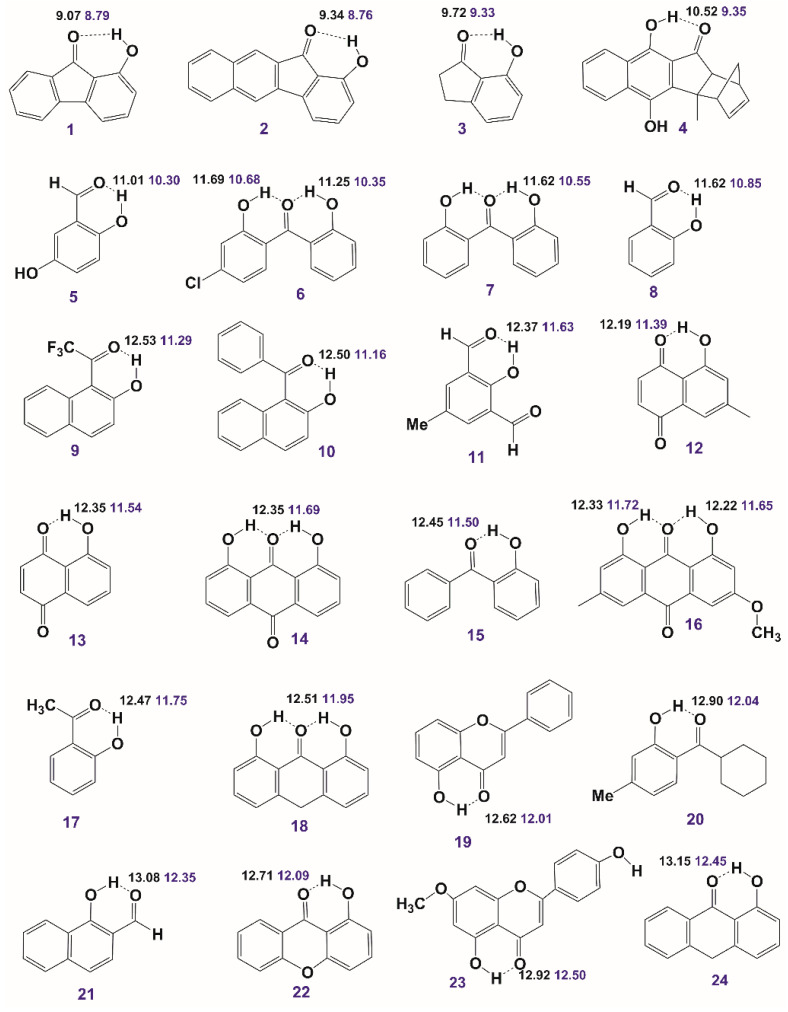

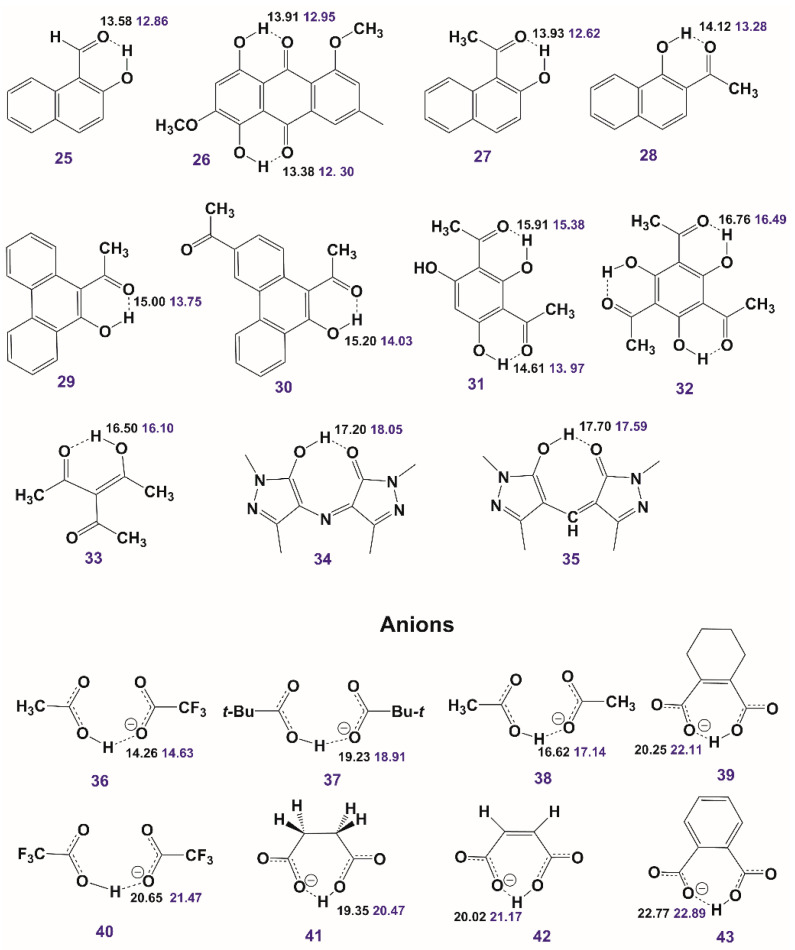
Chemical formulas of the phenol compounds exhibiting intramolecular O–H---O hydrogen bonds and ionic complexes with intramolecular and intermolecular O–H---^-^O hydrogen bonds. The data in black and blue are the computed ^1^H chemical shifts, ppm, with minimization of the structures at the B3LYP/6-31+G(d) and M06-2X/6-31+G(d) levels of theory, respectively. Reproduced with permission from [60]. Copyright 2015, The Royal Society of Chemistry, London, UK.

**Figure 37:**
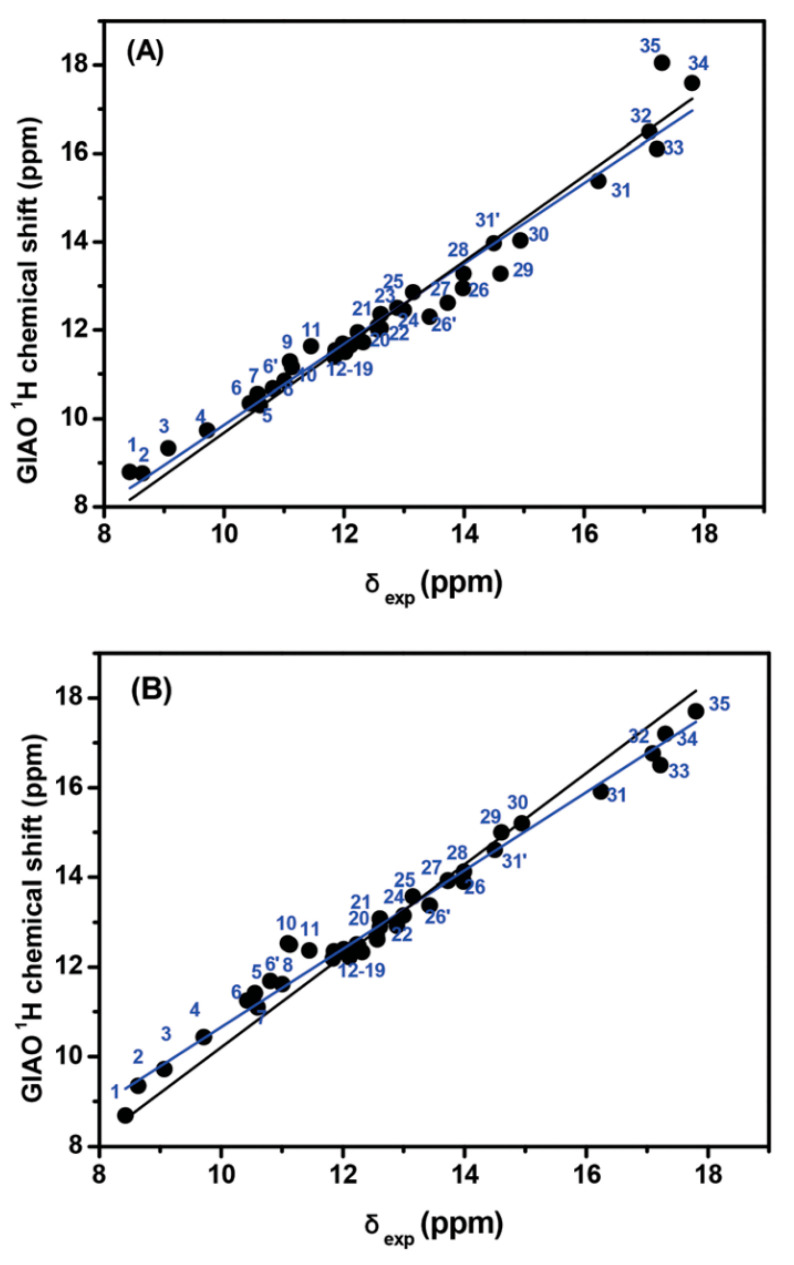
Calculated (at the GIAO/B3LYP/6-311G+(2d,p) level of theory with PCM in CHCl_3_) vs. experimental chemical shifts in the OH protons of the compounds **1–35** in Figure 36, with minimization of the structures at the M06-2X/6-31+G(d) (**A**) and B3LYP/6-31+G(d) (**B**) levels of theory. The blue line corresponds to the linear fit, and the black line to the linear fit through zero. Reproduced with permission from [60]. Copyright 2015, The Royal Society of Chemistry, London, UK.

**Figure 38:**
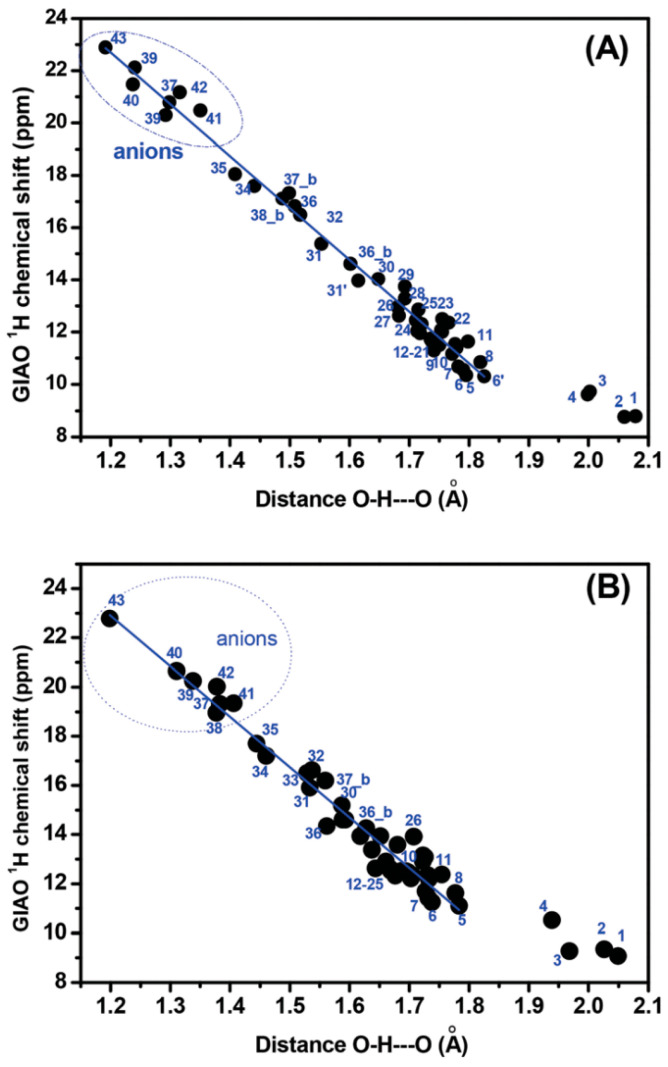
Calculated (at the GIAO/B3LYP/6-311+G(2d,p) level of theory with PCM in CHCl_3_) OH proton chemical shifts vs. calculated (O)–H…O distances, Å, of the compounds of Figure 36 with the minimization of the structures at the M06-2X/6-31+G(d) (**A**) and B3LYP/6-31+G(d) (**B**) levels of theory. Reproduced with permission from [60]. Copyright 2025, The Royal Society of Chemistry, London, UK.

Ionic inter- and intra-molecular hydrogen bonding has been extensively investigated, including ^1^H NMR spectroscopy [126,127,128,129,130,131,132]. DFT calculations usually fail in describing a wide range of negatively charged molecules due to the approximate form of the exchange–correlation functional [133,134]. Nevertheless, Siskos et al. [60,62] reported that the combination of DFT and CPCM in CHCl_3_, without incorporating discrete solvent molecules, can successfully predict ^1^H NMR chemical shifts, including O–H···^−^O ionic hydrogen bonding. Calculations were performed at the B3LYP/6-31+G(d) and M06-2X/6-31+G(d) levels of theory (Figure 36 and Figure 37) and, for selected examples, with the computationally more demanding MP2/6-31+G(d) level, which includes electron correlation effects. The calculated ^1^H NMR chemical shifts were not corrected for quantum zero-point vibrational effects (QZPVE), which have been shown to have a minor effect on GIAO DFT calculation of ^1^H NMR chemical shifts in water clusters (H_2_O)*_n_*, *n* = 2 to 16, with minimization at the MP2/6-311++G** level [131]. Figure 38 shows very good linear correlations between computed δ(OH) vs. computed hydrogen bond distance, with R^2^ of 0.966 and 0.960, and with energy minimization at the M06-2X/6-31+G(d) and B3LYP/6-31+G(d) levels, respectively [60].

Lacerda et al. [135] performed a detailed prediction of ^1^H and ^13^C NMR chemical shifts in protonated alkylpyrroles. The B3LYP functional was found not to be sufficient for all ^13^C chemical shifts, whereas the MP2 method resulted in excellent agreement with the experimental data. The simple PCM model was found to improve the agreement, with the exception of the NH proton, which requires the inclusion of an explicit solvent molecule in the calculation. The inclusion of a counter-ion deteriorates the agreement with the experimental data. No vibrational correction was applied since the combination of solvent and vibrational effects has not been settled.

Recently, Kaplanskiy et al. [51] reported a state-of-the-art investigation of solvation, dynamics and nuclear delocalization effects of strong ionic hydrogen bonds, using a combination of static quantum chemical calculations and ab initio MD calculations. Three systems were selected: the bifluoride anion (FHF)^−^, the Zundel cation (H_5_O_2_)^+,^ and the pyridine–pyridinum cation (PyHPy)^+^. Four solvation models were examined: vacuum, implicit solvent, explicit solvent, and cluster–continuum (Table 14). Continuum solvation models can provide only a first-order approximation to solvent effects. The effect of explicit solvation depends on the system, with maximum effect in the case of (PyHPy)^+^ due to more extended interaction with the solvent. Nuclear dynamics and delocalization are the primary factors determining δ_comp_(^1^H) in complexes with short and strong hydrogen bonding. Relativistic effects are not negligible in molecular systems that involve heavy atoms or strong electronic factors, as in the case of (FHF)^−^.

### 2.9. Hydroperoxides (R–O–O–H)

**Figure 39:**
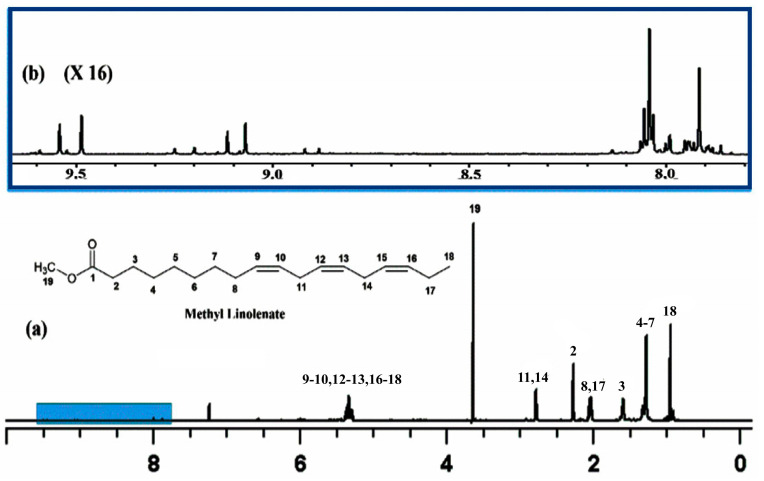
(**A**) 800 MHz ^1^H NMR spectrum of 20 mg methyl linolenate in CDCl_3_ subjected to heating at 40 °C for 48 h. Number of scans = 64, acquisition time = 1.02 s, experimental time = 6.5 min, and relaxation delay = 5 s, T = 298 K; (**B**) the selected region of the C-O-O-H resonances of its primary oxidation products [141].

**Figure 40:**
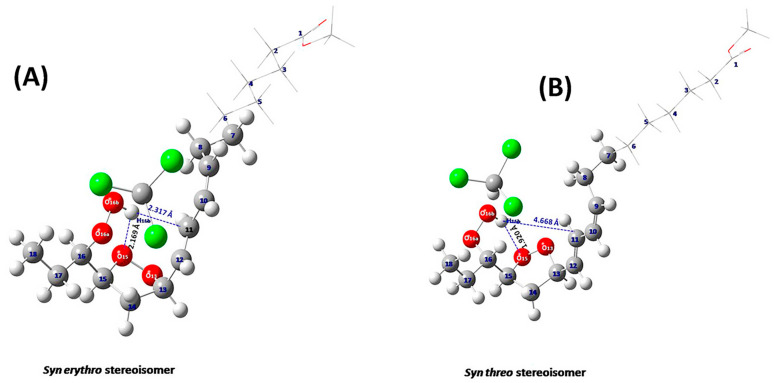
The structural comparison of the full-length syn erythro (**A**) and syn threo (**B**) endo–hydroperoxides, with a discrete solvation molecule of CHCl_3_ (energy minimization using the APFD/6-31+G(d):PM6 method) [141].

Hydroperoxides are a wide range of compounds in the form ROOH [136] which are of great importance in several natural products, such as polyunsaturated fatty acids, steroids and terpenes. Their homo- and heterolyctic decomposition is related to several inflammatory diseases [137,138,139,140]. Recently, Ahmed et al. [141] investigated methyl linolenate, which was allowed to oxidize in atmospheric oxygen for 48 h at 40 °C. The ^1^H NMR spectrum showed a significant number of sharp resonances (Δν_1/2_ ≤ 2.0 Hz) of the hydroperoxide protons in the deshielded region of 7.7 to 9.6 ppm (Figure 39). DFT and ONION [142] ^1^H NMR chemical shift computational studies, using the B3LYP functional and the G-311+G(2d,p) basis set with a discrete solvation molecule of CHCl_3_, were found to be in very good agreement with a stronger intramolecular hydrogen bond interaction between the hydroperoxide proton and the O15 oxygen of the five-member endo–peroxide ring of the major 15,16-threo than in the 15,16-erythro endo hydroperoxide molecule (Figure 40).

## 3. Nitrogen NMR and DFT Studies on Solvation Phenomena

**Figure 41:**
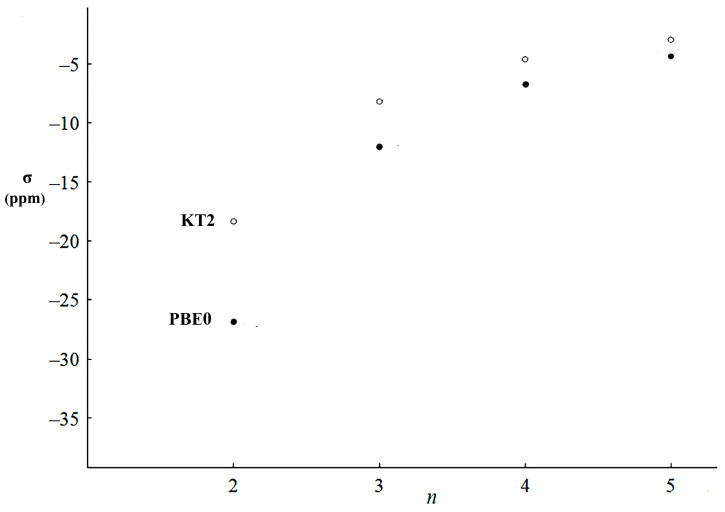
A plot of the derivative of the isotropic ^15^N magnetic shielding constant of pyridine in chloroform, calculated with KT2 and PBE0 DFT functionals, versus splitting order of Dunning’s cc-pVXZ basis set (X = D, *n* = 2; X = T, *n* = 3; X = Q, *n* = 4; X = 5, *n* = 5) [144].

Krivdin [143] comprehensively reviewed ^15^N NMR computational protocols with emphasis on the level of theory, the selection of density functionals and basis sets, solvent effects, rovibrational corrections, and relativistic effects. The correlation–exchange generalized gradient approximation Keal–Tozer’s functional KT2 and KT3, in combination with Jensen’s pcS-2 and pcS-3 basis sets for nitrogen atoms and pc-2 for the rest of the molecules, were recommended [144] (Figure 41). For non-specific solute–solvent interactions, the IEF-PCM level was suggested. In the presence of specific hydrogen bonding interactions, the use of explicit solvent molecules is necessary [144,145]. Rovibrational and relativistic effects are of minor importance in most practical cases.

### 3.1. Nitrogen Heterocycles

**Figure 42:**
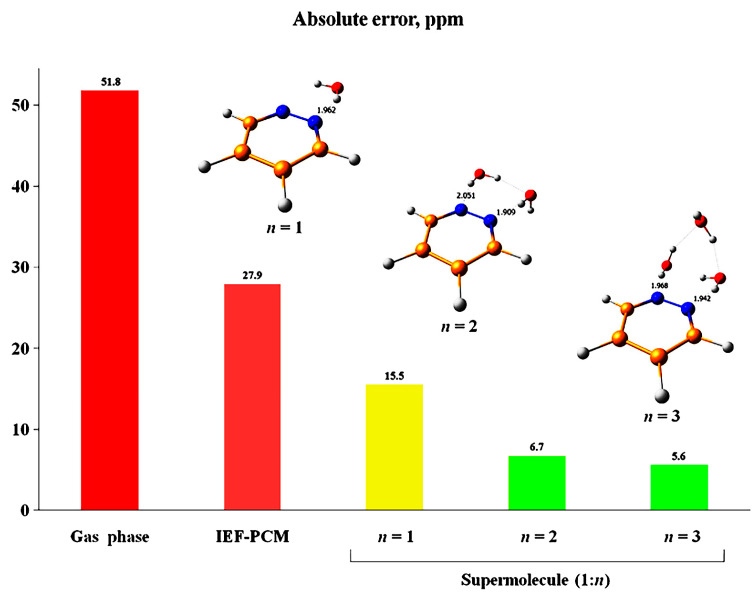
Absolute errors of ^15^N NMR chemical shift in pyridazine in water, calculated at the GIAO- KT3/pcS-3//pc-2 level using different solvation models to account for solvent effects. The most characteristic interatomic distances in the solvate complexes 1:1 (*n* = 1), 1:2 (*n* = 2) and 1:3 (*n* = 3) are given in Å. Reproduced with permission from [146]. Copyright 2014, Wiley, Chichester, West Sussex, UK.

A number of computational articles were published on pyrrole, pyridine, five- and six-member nitrogen-containing heterocycles, with emphasis on solvent effects [144,145,146]. Table 15 shows the effects of solvents on computational and experimental ^15^N NMR chemical shifts in pyridine and pyridazine. The gas phase calculations strongly deviate from the experimental data, especially with solvents that are capable of hydrogen bonding interactions (CH_3_OH, C_2_H_5_OH, and H_2_O). The inclusion of the IEF-PCM model improves the agreement with the experimental data; however, in protic solvents, deviations in the range of 11.8 to 14.6 ppm were obtained. This is due to the considerable contribution of specific hydrogen bonding with explicit solvent molecules, which is not sufficiently described by the IEF-PCM model. The use of a supermolecule (1:1) cluster results in a significant improvement in the experimental data for pyridine. For the pyridazine + H_2_O (1/1) cluster, the calculated chemical shift, although improved, strongly deviates from the experimental value (~15.5 ppm, Figure 42). The supermolecule clusters 1 pyridazine: *n* = 1, 2, … *n* H_2_O, placed into the IEF-PCM cavity, result in excellent agreement with the experimental data for *n* = 3, with a deviation of 5.6 ppm relative to 51.8 ppm obtained in the gas phase.

### 3.2. Amides and Amines

**Figure 43:**
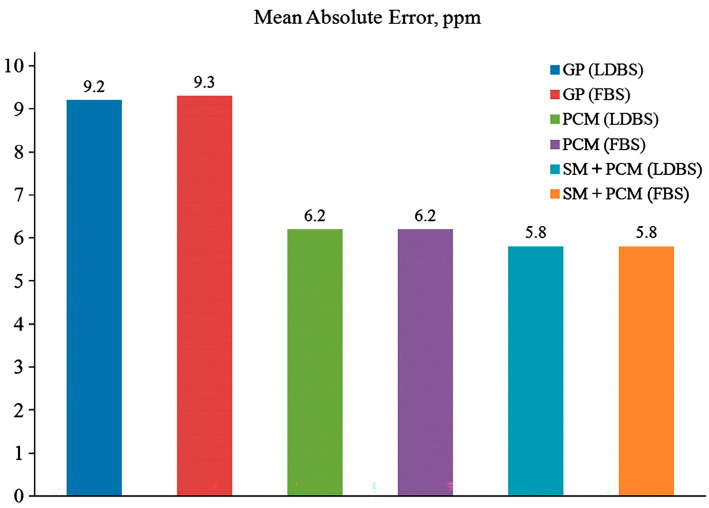
The mean absolute errors of the calculated ^15^N NMR chemical shifts in the series of 16 amides in the gas phase (GP) and in solutions within the polarizable continuum model (PCM) scheme and supermolecular model in PCM medium (supermolecular (SM) + PCM), using locally dense basis set (LDBS) and full basis set (FBS) schemes. Reproduced with permission from [147]. Copyright 2017, Wiley, Chichester, West Sussex, UK.

The GIAO ^15^N NMR chemical shifts in a series of 16 amides have been investigated, taking into account solvent effects with the PCM model and the supermolecule model (SM) in the PCM medium [147]. Using either the PCM or SM+PCM levels, agreement with the experimental data is somewhat improved; however, the deviation is still substantial (5.8 ppm) (Figure 43). Since the amide oxygen is a potential site of solvation, especially in H_2_O and methanol, further increase in the number of explicit solvent molecules may be required.

A series of open-chain aliphatic amines, five- and six-member saturated rings, and aromatic amines (aniline derivatives) were investigated in the framework of GIAO–DFT theory [148]. The influence of solvent on the accuracy of ^15^N chemical shifts was investigated in the gas phase, using the IEF-PCM model and the supermolecule approach with a single explicit molecule. The mean absolute errors of the calculated chemical shifts relative to the experimental values, Δδ(^15^N), were found to be ~13 ppm in the gas phase, ~9 ppm using the IEF-PCM model, and ~4.5 ppm using the supermolecule approach. The moderate performance of the supermolecule approximation could be attributed to the relatively small experimental solvent effect of 8–10 ppm, which is within the limits of the computational methods.

### 3.3. Effects of Protonation

**Figure 44:**
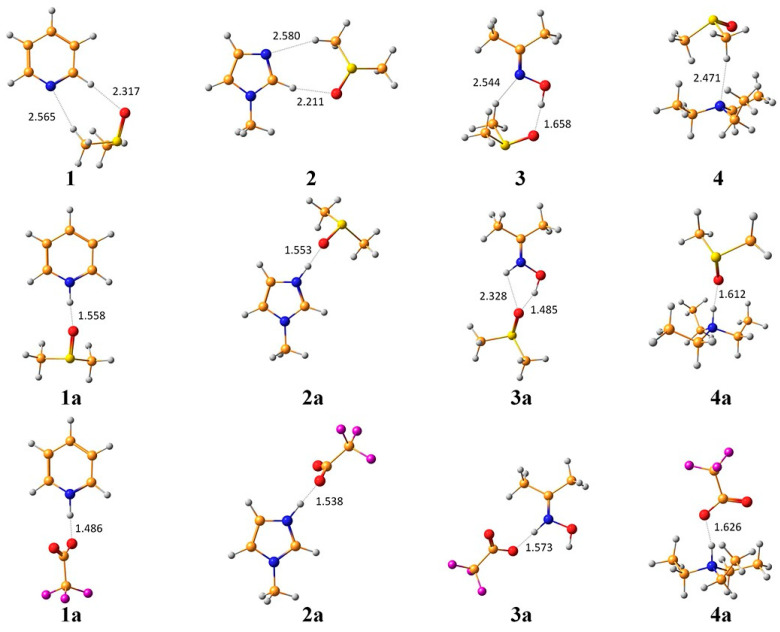
“Supermolecules” of compounds **1–4** and their protonated forms **1a–4a** with one solvate molecule (DMSO) and/or counter ion (CF_3_COO^−^) optimized at the MP2/6-311++G(d,p) level, taking into account bulk solvent effect within the IEF-PCM scheme. The interatomic distances are given in Å. Reproduced with permission from [151]. Copyright 2015, Wiley, Chichester, West Sussex, UK.

^15^N chemical shifts strongly depend upon the protonation state of the nitrogen atom, which is of considerable practical interest in the determination of the protonation state of a variety of nitrogen-containing organic and biological compounds. For sp^2^-hybridized nitrogens, such as in pyridine and Schiff bases, a shielding of about 100 ppm has been observed, while for amine sp^3^-type nitrogens, protonation results in deshielding of ~25 ppm [149,150]. Semenov et al. [151] performed a detailed ^15^N NMR computational and experimental investigation of protonation shifts in pyridine, **1**, N-methylimidazole, **2**, acetone oxime, **3**, and triethylamine, **4**. Figure 44 shows the structures of the supermolecules of the compounds **1**–**4**, and their protonation forms **1a**–**4a**, with the solvate molecule (DMSO) and/or counter ion (CF_3_COO^-^). Table 16 shows the protonation ^15^N shifts in the compounds **1**–**4** using different solvation models. The incorporation of a supermolecule in polarizable continuum resulted in a mean absolute error (MAE) of ~7 ppm, which can be compared with the MAE of ~20 ppm in the gas phase and ~13 ppm with the PCM and counter ion in PCM.

## 4. Oxygen–17 NMR and DFT Studies on Solvation Phenomena

Several review articles on ^17^O NMR have been published that cover a wide range of applications [152,153,154,155,156,157,158] or more specialized subjects [156,157,158]. ^17^O NMR chemical shifts in several functional groups are very sensitive probes of local electronic environment, including hydrogen bonding and non-bonding interactions; however, due to the extremely low sensitivity of ^17^O and the large linewidths, due to quadrupolar relaxation, selective enrichment is usually required, with the exception of small molecular weight molecules. Thus, several theoretical models and computational methods have been discussed in relation to solvation phenomena of simple prototype organic molecules.

### 4.1. H_2_O and Alcohols

**Figure 45:**
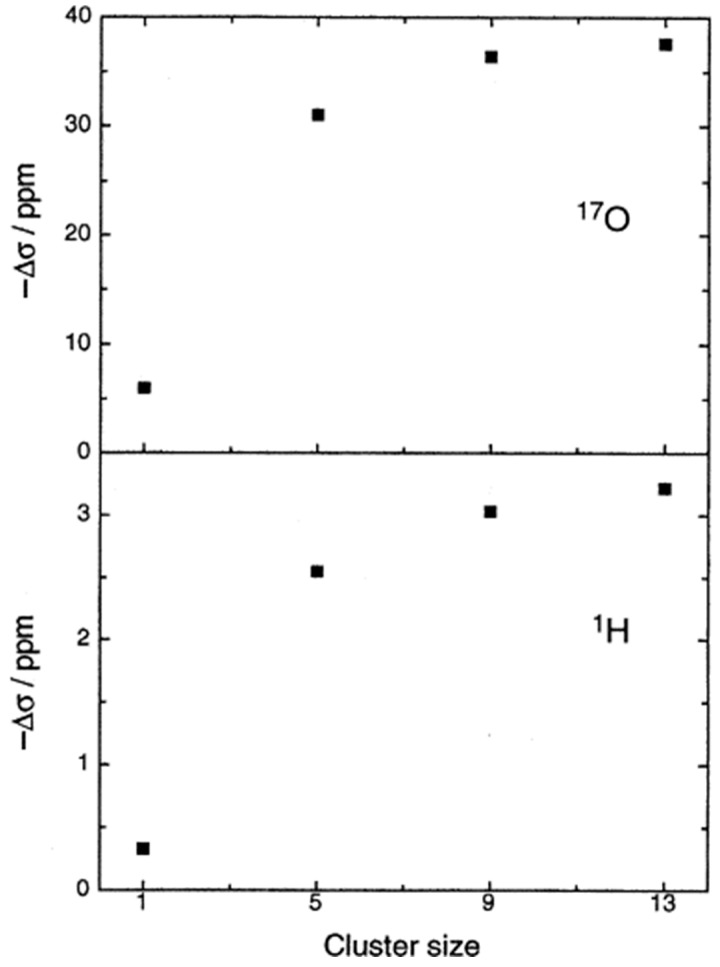
^17^O (top) and ^1^H (bottom) calculated shifts Δσ vs. cluster size for “liquid” water, showing the convergence of the shifts at clusters of size 13. Reproduced, with permission, from [159]. Copyright 1996, Wiley-VCH, Verlag GmbH & Co., Weinheim, Germany.

The ^17^O NMR chemical shift in H_2_O in the gas phase shows a strong shielding of −36.1 ppm relative to liquid H_2_O; thus, it can provide an excellent prototype molecule for investigating the accuracy of various computational methods in solvation phenomena. An early calculation based on a combination of DFT and molecular dynamics (MD) simulations [159] showed a convergence of the shifts with clusters of size 13 (Figure 45). The best agreement with the experimental shielding was obtained with a cluster of nine molecules of H_2_O with the LC ab initio potential. Pfrommer et al. [160] performed DFT ab initio Car-Parrinello MD simulation [161], which models liquid and solid H_2_O not as finite clusters but as infinite periodic systems. Gas-to-liquid chemical shifts of −5.8 ± 0.1 ppm and −36.6 ± 0.5 ppm for ^1^H and ^17^O, respectively, were calculated. Pennanen et al. [162] performed ab initio Car–Parrinello MD simulations to produce clusters with different numbers of H_2_O molecules. Subsequent HF and DFT studies, the latter using the hybrid B3LYP functional, were employed to obtain the shielding for the central molecule of each cluster. The ^17^O gas-to-liquid shift of −41.2 ± 1.4 ppm was obtained, which differs from the experimental value by ~5 ppm. Karadanov [163] investigated the importance of electron correlation and the basis set in the case of hydrogen bonding ^17^O nuclei in water clusters (H_2_O)*_n_* (*n* = 2–5), using HF and MP2 approaches with GIAO/6-31+G(d,p), 6-31++G and 6-311++G(2d,2p) basis sets. The inclusion of correlation effects and diffuse functions is essential for hydrogen bonding ^17^O nuclei. Bilalbegovic [164] investigated ^17^O NMR chemical shifts in several isomers of water hexamer clusters, using DFT and the gauge, including the projector augmented wave (GIPAW) pseudopotential method. The ^17^O chemical shifts show a substantial variation up to 5.8 ppm for the six more stable hexamers. Klein et al. [165] performed a detailed analysis of the limits of the PCM theory in ^17^O NMR chemical shifts in H_2_O in the condensed liquid and solid state due to solute–solvent cooperative charge transfer. DFT studies on water clusters in which cooperative hydrogen bonding was taken into account resulted in ^17^O chemical shifts in agreement with the change from monomeric H_2_O to that in high-pressure ice. More recently, de Dios [166] performed ^17^O NMR chemical shift calculations for H_2_O in dimer, trimer, tetramer, and pentamer supermolecules at the MP2 level, using an aug-CC-pVTZ basis set. As expected, the ^17^O chemical shifts in H_2_O are sensitive to hydrogen bond distance and the OH covalent bond.

**Figure 46:**
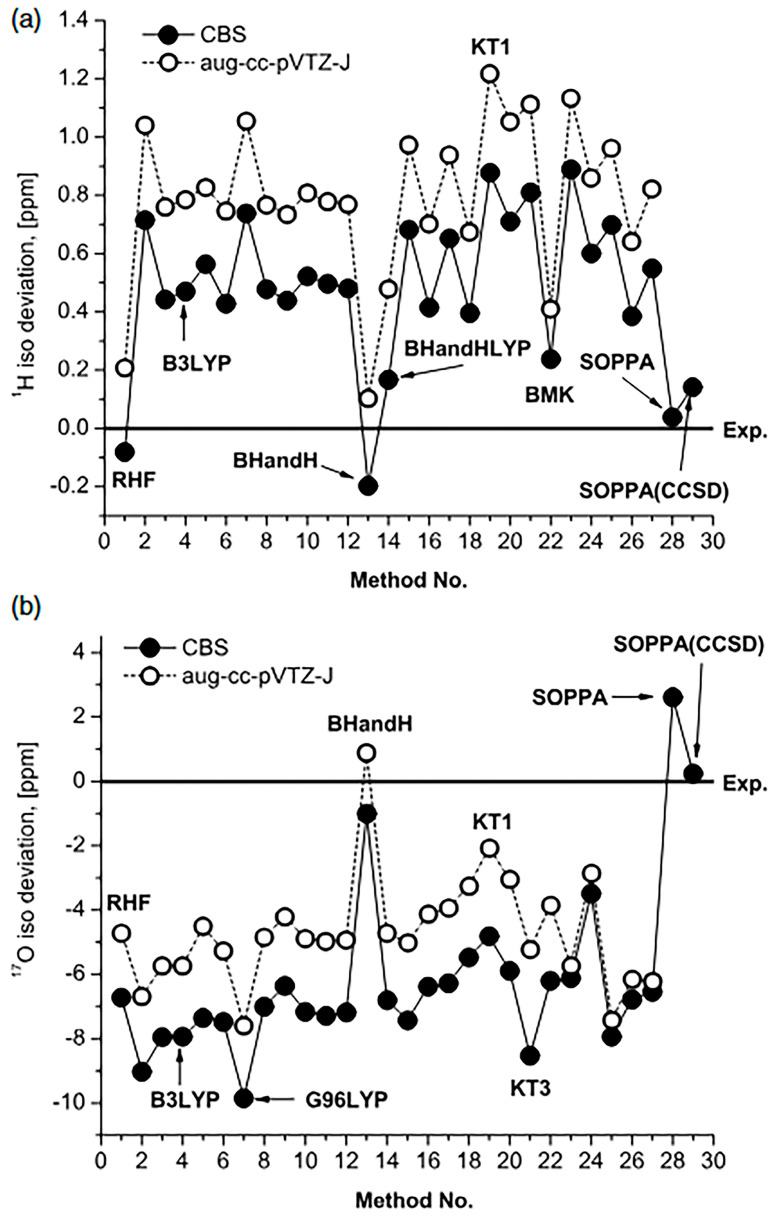
(**a**) Deviations in complete basis set (CBS)-calculated ^1^H and (**b**) ^17^O isotropic shieldings of monomeric H_2_O from experimental values, using the pcJ-n basis sets and the smaller aug-cc-pVTZ-J basis set for different methods of calculation. For the method, see Ref. [166]. Reproduced with permission from [166]. Copyright 2009, Wiley, Chichester, West Sussex, UK.

Isotropic chemical shifts in monomeric H_2_O were reported [167] using correlation-consistent and polarization-consistent basis sets in the Kohn–Sham set limit. The performance of BHandH was shown to be the optimum one, with deviations from experimental values of −0.2 ppm and −1.0 ppm for ^1^H and ^17^O, respectively (Figure 46). Similarly, high-level computations were performed for isolated methanol [168,169] with very good agreement with the experimental value [170]. It would be of interest to extend the above methodologies from isolated molecules to solvated supermolecule species.

### 4.2. Carbonyls

Cossi and Crescenzi [171] reported an analysis of different models for the calculation of solvation effects in aqueous solution for molecules with sp^3^ and sp^2^ oxygens. Both cluster + PCM and MD + PCM calculations showed that the use of PCM has a notable effect on the final result. When the MD was used to sample different solvent conformations, the convergence with respect to the number of solvent molecules was much faster when the PCM bulk effects were included in the calculation. The solvent shielding shift of acetone with the use of two explicit molecules of H_2_O without PCM (−54.2 ppm at the 6-311+G(d,p) level, Table 17) underestimates the experimental shielding of −75.5 ppm of gas-to-aqueous solution [171,172]. The solvent shielding shift of acetone with the use of two explicit molecules of H_2_O with PCM (−94.2 ppm at the 6-311+G(d,p) level), overestimates the experimental shielding strongly.

Pavone et al. [173] developed and validated a modified AMBER force field for an integrated MD/DFT/PCM approach for investigating solvent effects on the UV and ^17^O NMR spectra of acetone. Calculations were carried out on acetone-(H_2_O)*_n_* clusters, using PCM to account for bulk solvent effects. For ^17^O calculations, the PBE0 hybrid functional with the G-311+G(d,p) basis set was used. Two explicit molecules of H_2_O, in combination with PCM, resulted in shielding of the gas-to-aqueous solution of acetone of −80.0 ppm relative to the experimental value of −75.5 ppm. No significant difference was noted when the number of explicit water molecules was increased to five.

### 4.3. Amides

**Figure 47:**
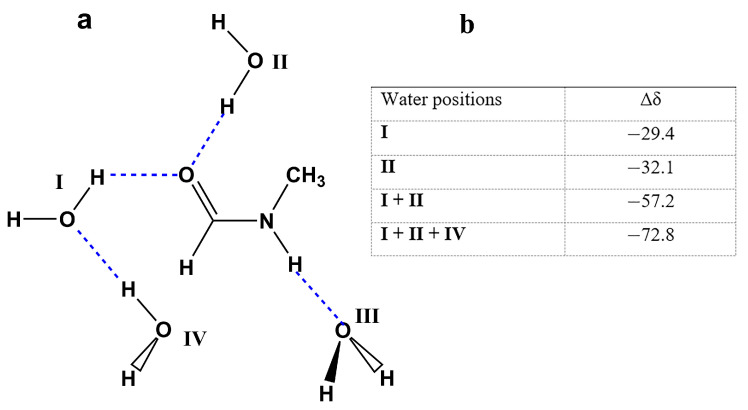
(**a**) A drawing of the NMF + (H_2_O)_4_ system with four in-plane water molecules, and (**b**) hydration shifts, Δδ (ppm), for the amide oxygen in NMF, calculated at the GIAO level [178].

^17^O NMR chemical shifts in amides and peptides have been shown to be very sensitive to hydrogen bonding interactions and solvation phenomena, and several empirical correlations have been reported [174,175,176,177,178]. The presence of the carbonyl and the amine groups suggests the possibility of the formation of the two types of association with water. One in which the H_2_O molecules act as a proton acceptor in hydrogen bonding to the N–H group, and the other in which the H_2_O acts as a proton donor in hydrogen bonding to the carbonyl oxygen. Computational studies on amide solvation up to 1993 were summarized in a review article [174]. Ab initio calculations of the ^17^O hydration chemical shifts in NMF + (H_2_O)_4_ with four in-plane molecules of H_2_O (Figure 47) were performed at the GIAO level, using the 6-311+G** basis set [178]. The hydration shifts in NMF were compared with the classical dipolarity/polarizability, hydrogen bond donor (HBD) acidity, and hydrogen bond acceptor (HBA) terms [179]. The computational data reproduced the experimental trends satisfactorily (Figure 47).

Cossi and Crescenzi [180] investigated the solvent effects of ^17^O shieldings on N-methylformamide in polar and apolar media, using the GIAO/PBE0 or PBE1PBE functionals with the 6-311+G(d,p) basis set. In non-protic solvents, e.g., CHCl_3_, the experimental data relative to CCl_4_ are reproduced satisfactorily by the PCM model: Δδ_calc_(CHCl_3_) −22.5 ppm, Δδ_exp_ (CHCl_3_) −23.9 ppm. In protic solvents, and especially in water, the computational PCM data (−39.9 ppm) deviated strongly from the experimental data (−71.5 ppm). The computational chemical shift differences in H_2_O relative to CCl_4_ for clusters with one or two explicit molecules of H_2_O hydrogen bonding on the CO group, with and without PCM, were also investigated. The cluster with two explicit molecules of H_2_O in PCM (−62.0 ppm) showed a reasonable agreement with the experimental data (−71.3 ppm).

**Figure 48:**
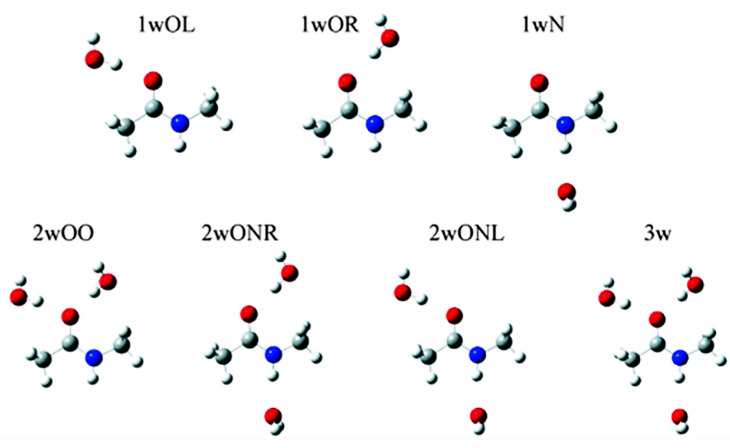
A schematic presentation of the structures of the NMA-*n*w clusters, with indication of the corresponding labels. Reproduced with permission from [181]. Copyright 2005, The American Chemical Society, Washington, DC, USA.

**Figure 49:**
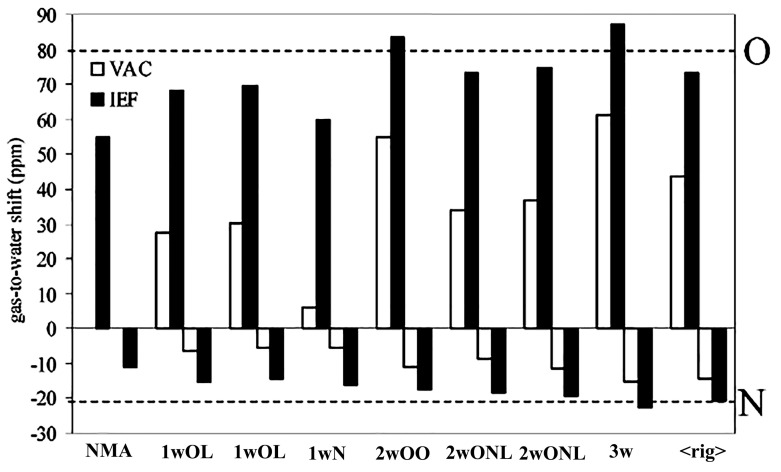
Gas-to-water shifts for oxygen (O) and nitrogen (N) using various descriptions:  a continuum only model (NMA), QM hydrogen-bonded clusters (*n*wXY, with X,Y = O,N and *n* = 1, 2, 3) and MD–clusters (<rig>). Each set of clusters has been studied in the gas phase (VAC) or with an external continuum (IEF). Experimental estimates are reported as dotted lines. Reproduced with permission from [181]. Copyright 2005, The American Chemical Society, Washington, DC, USA.

Mennucci and Martinez [181] performed a ^17^O and ^15^N quantum mechanical and molecular dynamics study on N-methylacetamide in water and acetone, using three different solvation models: the IEF-PCM model, the hydrogen bonding supermolecule approach, and the combined supermolecule/continuum approach. The selected structures of NMA-*n*w (w denotes H_2_O) are shown in Figure 48, and the resulting gas-to-water chemical shift differences for ^17^O and ^15^N are presented schematically in Figure 49. In the gas phase, all the supermolecule clusters with QM or MD strongly deviate from the experimental data for both ^17^O and ^15^N. In the 3w cluster, with all the available sites hydrogen bonded to H_2_O molecules, the gas-to-water chemical shift for ^17^O also deviates from the experimental value. It was concluded that the hydration of NMA is more sufficiently presented in terms of two types of molecules of H_2_O: a more static (rigid) water, which is explicitly described, and more mobile water, whose effects are described through IEF-PCM.

### 4.4. Nucleobases

**Figure 50:**
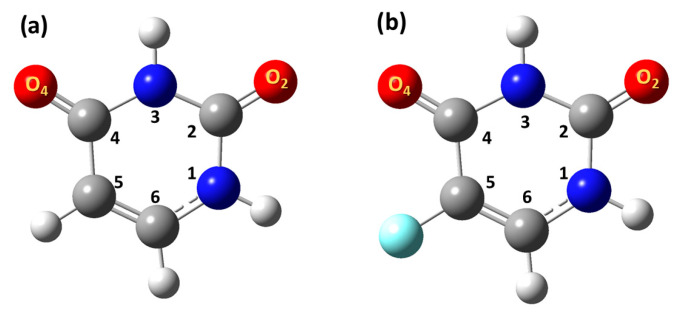
Structures of uracil (**a**), and 5-fluorouracil (**b**). The numbering of the oxygen atoms is also used in Table 18.

Bednarek et al. [182] reported experimental and computational (at the HF and MP2 GIAO level) ^1^H, ^13^C, ^15^N and ^17^O chemical shifts in 5-halogen uracils. The solvent effects were investigated at the HF/3-21G** level for the uracil + 7 H_2_O supermolecule. Gester et al. [183] used different solvation models to investigate ^17^O shielding of uracil and 5-fluorouracil (Figure 50) in H_2_O at 95 °C [184], using a sequential quantum mechanics/molecular mechanics methodology (S-QM/MM) [185]. Optimum results were obtained using the mPW1PW91 density functional with the aug-pcS-2 basis set (Table 18). The use of the PCM to include the polarization effects in combination with the average solvent electrostatic configuration (ASEC) model resulted in good agreement with the experimental data, with sufficient computational performance [183].

### 4.5. Organic Acids and Peracids

^17^O NMR chemical shift computations of aqueous acetic, paracetic, lactic and perlactic acids were used to facilitate the resonance assignment of experimental spectra [186]. Further computational studies [187] included a comparison of several solvation models of water, hydrogen peroxide, acetic acid, lactic acid and paracetic acid. The PBE0 functional with the 6-311+G(d,p) basis set was shown to be a very good compromise between accuracy in shielding calculations and computational cost. The PCM approach is not appropriate for molecular systems with hydrogen bonding interactions, in agreement with previous studies. Nevertheless, the PCM approach is useful to reduce the number of explicit solvent molecules in the calculation. The MD approach was found to be an improvement, while the mixed molecular dynamics simulation, followed by a 3-step ab initio optimization of the extracted clusters, can result in average computational chemical shifts within 10 ppm of the experimental data [188]. This difference can further be minimized by adopting more accurate QM/MM models [187]. An alternative approach would be to place a limited number of H_2_O molecules in the vicinity of the COOH group, based on chemical intuition, which has been efficaciously applied in solvation studies on monounsaturated and ω-3 polyunsaturated free fatty acids in CHCl_3_ and DMSO solution [113] (Table 13).

### 4.6. Amino Acids

**Figure 51:**
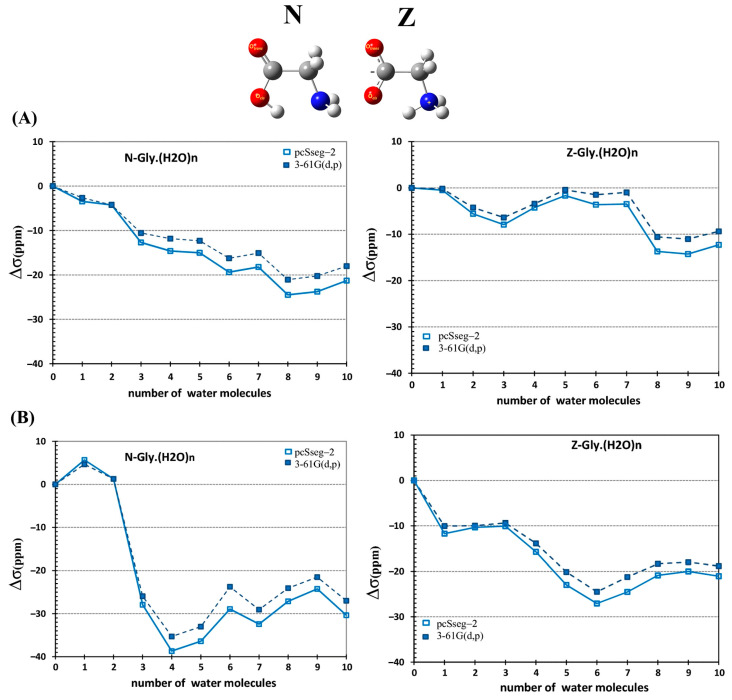
The change in the magnetic shieldings (in ppm) of: (**A**) cis oxygen for the neutral N- (**left**) and zwitterionic Z- (**right**) glycine in water solution, and (**B**) trans oxygen for N- (**left**) and Z- (**right**) glycine in water solution, using various numbers of explicit water molecules in the calculation: the pcSseg-2 (solid line) and the 3-61G(d,p) (dashed line) basis sets [190].

Glycine has been extensively investigated as a model of the effect of solvation on the zwitterionic-neutral equilibrium, using a variety of computational models [189]. Caputo et al. [190] investigated ^13^C, ^15^N and ^17^O shieldings of glycine, using the B3LYP functional with the 6-31G(d,p) and pcSseg-2 basis sets. The effect of the solvent was investigated in neutral (N) and zwitterionic (Z) glycine (Figure 51) with the PCM approach or PCM with explicit water molecules hydrogen bonding to the solute. The largest shielding changes upon increasing the number of explicit molecules of water were calculated for the oxygen nucleus. The trans O_t_ oxygen is the most sensitive to the presence of explicit molecules of H_2_O and shows the largest difference from the PCM prediction. For the Z-ionization state of glycine, which is the major species in aqueous solution, there is a decrease in shielding by −19 and −10 ppm for the trans and cis oxygen atoms, respectively (Figure 51). The shieldings tend to converge with the increase in the number of explicit solvent molecules.

## 5. Phosphorous–31 NMR and DFT Studies on Solvation Phenomena

**Figure 52:**
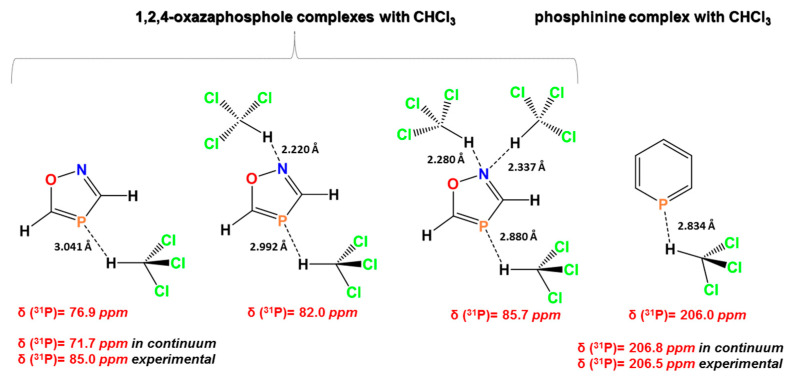
Representative solvation complexes of 1,2,4-oxazaphosphole with one, two, and three explicit molecules of chloroform and the phosphinine–CHCl_3_ complex. All interatomic distances are given in Å. Geometry optimizations were performed at the MP2/aug-cc-pVTZ level.

The prediction of ^31^P-NMR chemical shifts for phosphorous-containing molecular systems is not a trivial task; thus, Rusakova and Rusakov, in a recent review article [191], reported several factors that affect the accuracy of ^31^P NMR chemical shift calculations, e.g., basis sets, geometry factors, vibrational, and relativistic effects. The solvent effects are also important, both as implicit and implicit–explicit mixed models, as well as macro solvation using QM/MM, QM-PCM and MD/DFT protocols. Rusakov et al. [192] calculated ^31^P NMR chemical shifts in 1,2,4-oxazaphosphole by adding successively one, two, and three explicit molecules of chloroform. δ_comp_(^13^P) consecutively increased from 71.7 ppm in IEF-PCM to 76.9, 82.0, and 85.7 ppm, the latter being in very good agreement with the experiment value of 84.0 ppm (Figure 52). The geometry optimization of all complexes was carried out at the MP2/aug-cc-pVTZ level, and the ^31^P NMR chemical shifts at the DFT level, using OLYP functionals within the aug-pcS-3/aug-pcS-2 Locally Dense Basis Set (LDBS). In the case of phosphinine (in continuum) and the phosphinine–CHCl_3_ complex, the values are comparable (Figure 52).

Maryasin and Zipse [193] calculated δ(^31^P) of phosphanes and related compounds using the PCM model, as well as with the inclusion of one to three explicit solvent molecules. The addition of only one solvent molecule, at selected positions, in combination with the PCM, led to a very good agreement of the computational value (29.6) ppm, with the experimental value (29.7 ppm) of triphenylphosphine oxide in chloroform. Minimization was performed at the MPW1K/6-31G(d) level and ^31^P calculations at the GIAO-MPW1K/6-311++G(2d,2p) level. The PH_3_ was used as a reference compound. The solvent effects on ^31^P NMR parameters of Ph_3_P=O oxide and Ph_3_P in chloroform were investigated by testing different solvation models [194]: vacuum, implicit solvation with PCM, explicit micro-solvation plus PCM, and macro-solvation using both QM/MM and QM-PCM schemes. The QM/MM methods were found to be very promising for the prediction of phosphorus chemical shifts and could improve the precision of ^31^P NMR spectra simulations. Precechtelova et al. [195] and Fukal et al. [196,197] employed the classical molecular dynamics MD/DFT, which are time-consuming protocols, for the conformational averaging of phosphorus-containing compounds with explicit solvation molecules.

## 6. Computational vs. Experimental Activation Energies, ΔG^‡^, as a Tool for the Role of Solvents in Atomistic Reaction Mechanisms

**Figure 53:**
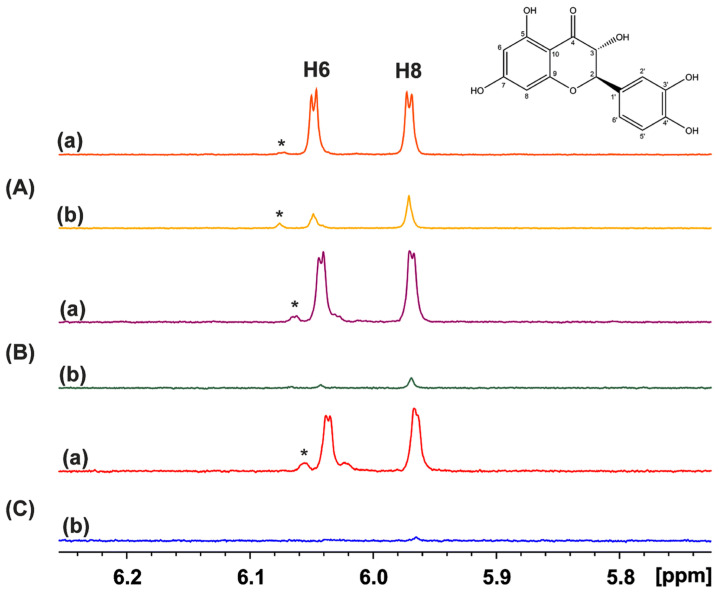
^1^H NMR (500 MHz) spectral region illustrating H-6 and H-8 H/D exchange of taxifolin (2.5 mM) in D_2_O, phosphate buffer (25 mM) and pD = 6.00. The spectra (**A**) (a) (T = 313 K), (**B**) (**a**) (T = 323 K), and (**C**) (**a**) (T = 328 K) were recorded at a time interval of 0 h. The spectra (**A**) (**b**) (T = 313 K), (**B**) (**b**) (T = 323 K), and (**C**) (**b**) (T = 328 K) were recorded at a time interval of 14 h. The asterisk denotes an impurity. Reproduced with permission from [204]. Copyright 2025, The Royal Society, London, UK.

**Figure 54:**
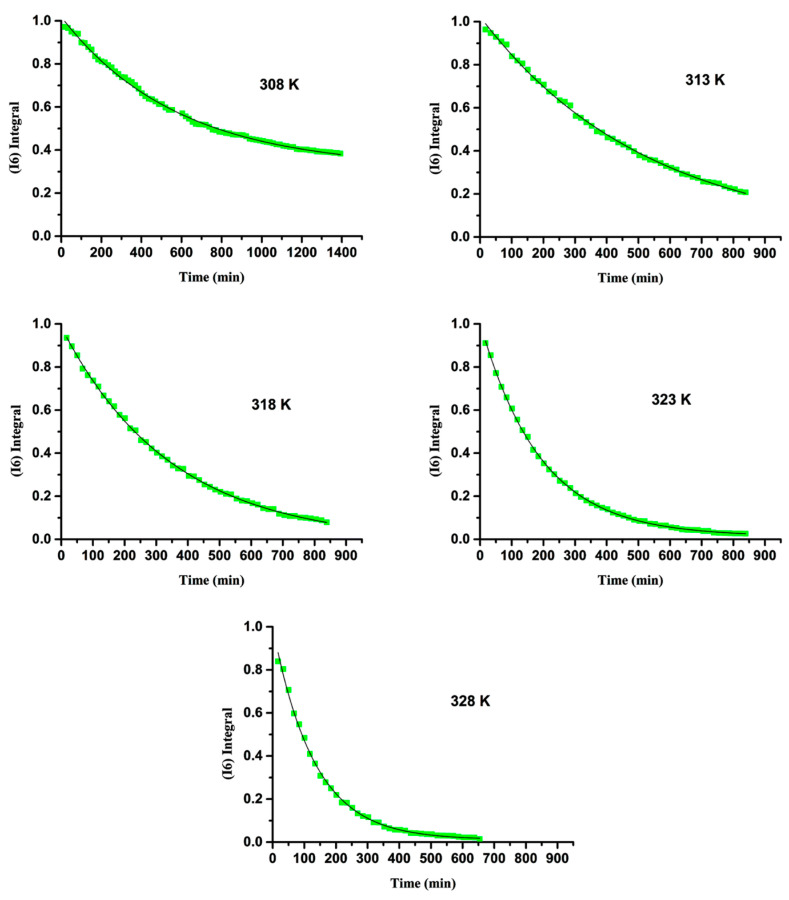
H-6 H/D exchange kinetic curves of taxifolin (2.5 mM) in D_2_O, phosphate buffer (25 mM), and pD = 6.00 at various temperatures. Reproduced with permission from [204]. Copyright 2025, The Royal Society, London, UK.

**Figure 55:**
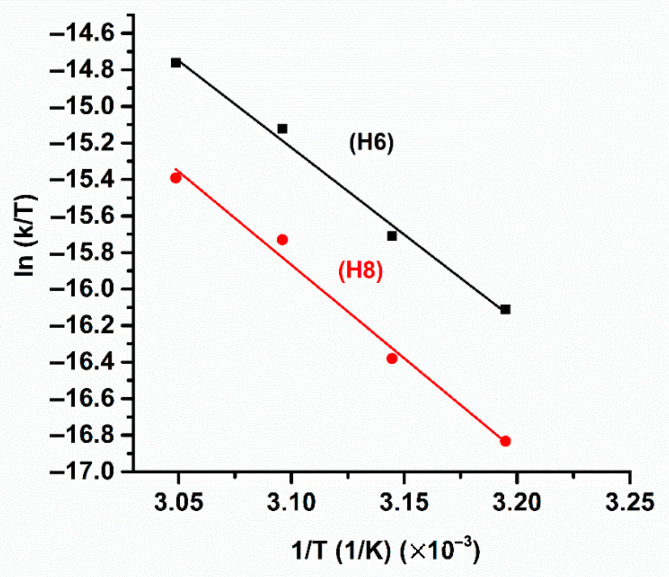
Eyring plots of the H-6 (*R*^2^ = 0.988) and H-8 (*R*^2^ = 0.982) of taxifolin (2.5 mM) in D_2_O, phosphate buffer (25 mM), and pD = 6.00. Reproduced with permission from [204]. Copyright 2025, The Royal Society, London, UK.

The important role of solvents, and especially water, in enhancing the rate and/or selectivity of numerous reaction processes has been extensively emphasized in the recent literature [198,199,200]. Acid-base-catalyzed H/D exchange in aromatic systems has been broadly utilized in the last six decades. Even so, the catalytic role of H_2_O was emphasized only recently in the deuteration of aromatic rings through keto–enamine tautomeric equilibrium [201]. Benaldo et al. [202] reported a combination of experimental ^1^H NMR kinetic studies and DFT calculations with a single solvation molecule of H_2_O in H/D exchange of flavonoids. Fayaz et al. [203] and Chalkidou et al. [204] reported detailed variable temperature and pH ^1^H NMR studies on the H-6 and H-8 protons of ring A in the natural products catechin and taxifolin (Figure 53) and phloroglucinol. The decay of the signals of H-6 and H-8 protons as a function of time and at various temperatures (Figure 54) follows first-order kinetics with an increase in the exchange rate at pD = 9.60, as compared to pD = 6.00. According to the Eyring equation:

(1) $$ln\frac{K}{T}=-\frac{\Delta H_{exp}^{‡}}{R}\frac{1}{T}+ln\frac{k_{B}}{h}+\frac{\Delta S_{exp}^{‡}}{R}$$

where k_B_ is the Boltzmann constant, T is the absolute temperature in Kelvin (K), is the rate constant in s^−1^, R is the ideal gas constant, and h is Planck’s constant. The values of $\Delta H_{exp}^{‡}$ and Δ$S_{exp}^{‡}$ can be determined from a $ln\frac{K}{T}$ vs. $\frac{1}{T}$ plot (Figure 55). The resulting $\Delta H_{exp}^{‡},$ − TΔ$S_{exp}^{‡}$ and $\Delta G_{exp}^{‡}$ are shown in Table 19.

**Figure 56:**
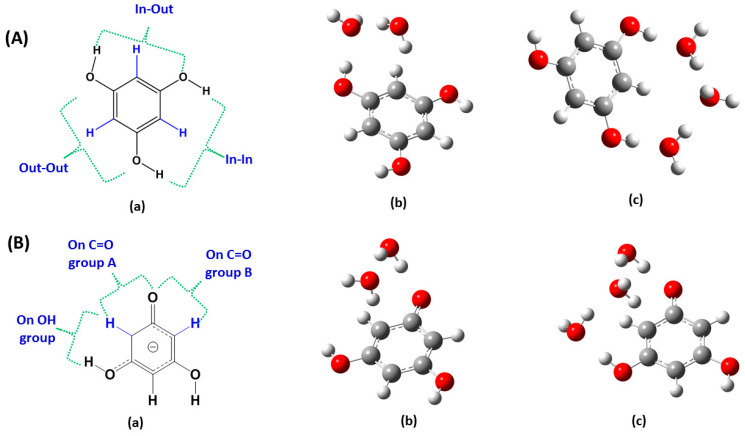
(**A**) (**a**) The definition of the “in–out”, “in–in”, and “out–out” configurations of the phenolic OH groups of neutral phloroglucinol, and (**b**) phloroglucinol + 2H_2_O, and (**c**) phloroglucinol + 3H_2_O in the “in–in” configuration. (**B**) (**a**) The definition of the “on–OH”, “on, C=O group A” and “on, C=O group B” configuration of the phloroglucinol anion; (**b**) phloroglucinol anion + 2H_2_O; and (**c**) phloroglucinol anion + 3H_2_O with the C=O group B configuration. The optimization of the structures at the B3LYP/6-31+G(d)/GD3BJ level [204].

DFT calculated ΔG^‡^ values without explicit molecules of H_2_O were found in the range of 62 to 50 kcal mol^−1^, which deviated strongly from the experimental values [203]. In the neutral form of the phloroglucinol, several reaction mechanisms were examined for the phenolic OH groups with “in–in”, “in–out”, and “out–out” configurations (Figure 56A). At the B3LYP/6-31+G(d)/GD3BJ level, the use of two molecules of H_2_O resulted in $\Delta G_{calc}^{‡}$ = 23.15 kcal mol^−1,^ which can be compared with $\Delta G_{exp}^{‡}$ = 20.96 kcal mol^−1^ (Table 20). With three molecules of H_2_O in the “in–in” configuration of the phenolic OH groups, two major arrangements of the H_2_O molecules were obtained with $\Delta G_{calc}^{‡}$ = 21.66 (46.8%) and 21.54 (38.19%) kcal mol^−1,^ which are in excellent agreement with the experimental value (Table 20).

**Figure 57:**
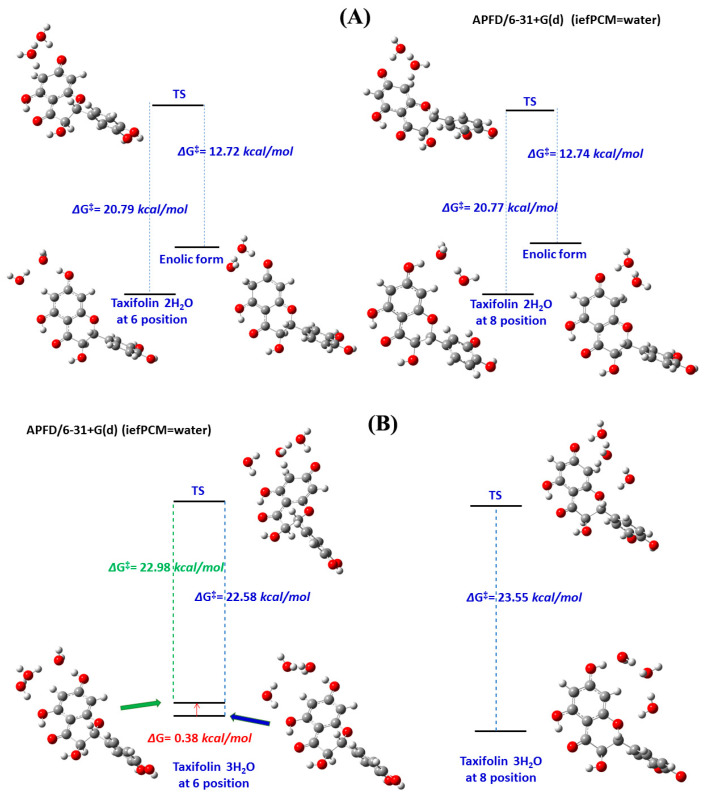
The mechanistic pathway of the aromatic C(8)-H and C(6)-H hydrogen exchange process for the complex of the neutral taxifolin with two (**A**) and three (**B**) molecules of H_2_O at the APFD/6-31+G(d) level [204].

The case of taxifolin was simpler from the mechanistic point of view than that of phloroglucinol, since the C(5)-OH group exists in a single orientation due to the formation of a strong intramolecular hydrogen bond with the C(4)=O group [203,204]. With three molecules of H_2_O, the C(6)-OH, and C(8)-OH, the $\Delta G_{calc}^{‡}$ values were in very good agreement with the experimental values at the APFD/6-31+G(d) level (Table 20, Figure 57). The enthalpic term $\Delta H_{calc}^{‡}$ was significantly larger than the entropic term − TΔ$S_{calc}^{‡}$ and was in very good agreement with the experimental data. This allowed the various mechanistic pathways to be evaluated at the atomic level and the generally accepted acid/base-catalyzed H/D exchange mechanism [205] to be brought into question. It would be of interest to apply the same methodology to investigate the role of bound molecules of H_2_O on the increase in H/D exchange of the self-association of catechins [206] and on the chiral stability of H/D exchange in acidic-containing compounds [207].

## 7. Software

The following software packages have been used in the international literature for the calculations of solvation phenomena:

Gaussian 94 [159]; Gaussian 98 [41,163,165,182,185]; Gaussian 03 [55,59,60,102,107,109,165,167,169,173,181,183,186,187]; Gaussian 09 [44,61,62,63,64,72,90,91,93,95,96,108,113,114,120,123,135,141,144,145,147,148,151,190,203,204]; Gaussian 16 [51,70,71,110,113,204]; DALTON 2.0 [44,167,168]; DALTON 2011 [144]; DALTON 2013.1 [146,147,148,151]; GAMESS (US) [146,147,148]; GAMESS (US) R.1 2013 [151]; ADF [102,145]; Turbomole 6.2.33 [133]; Spartan 02 [150]; QUANTUM ESPRESSO (QE) 2009 [164]; QUANTUM ESPRESSO (QE) 2017 [134] and Schrödinger’s Maestro 2019.1 [69].

For visualization and quantification of chemical bonds and properties from electron density data:

QTAIM Version 19.10.12 [70,71,90,91]; AIMAII Version 19.10.12 [70,71]; AIMAII Version 16.10.31 [90,91]; AIM2000 [166], a program to analyze and visualize atoms in molecules; and Morphy98 [166], a program to analyze molecular charge density.

For MD simulations:

NVT ensemble [96,159,181]; CPMD v 3.5 [160,161,162,173]; CP2K 2024.1 [51]; DL_POLY 2.13 [181]; pDynamo [187]; FINGER Code [162]; PACKMOL 2009 [51] for building initial configurations for molecular dynamics simulations; and TRAVIS 2011 [51], a free analyzer and visualizer for Monte Carlo and Molecular Dynamics Trajectories.

For Mode Carlo simulations:

DICE Version 2.9 [135,183]; DICE [185].

For Molecular Mechanics:

Amber 94 [181]; Amber 7 [173]; Amber 16 [96].

For calculations of chemical shifts:

Charge NMR program [108] and deMon [159].

## 8. Conclusions and Prospects for Future Research

From the results presented in the preceding sections, combined with our personal experience, the following conclusions can be drawn:

1. DFT calculations of solvent-dependent chemical shifts in hydrogens bonded to oxygen (e.g., phenolic OH, alcoholic OH, carboxylic COOH, and hydroperoxide O-O-H) and hydrogens bonded to nitrogen (e.g., amide CONH and amine and pyrimidine bases) can provide an excellent method to investigate complex solvatomer structures due to: (a) the large chemical shift deshielding up to 21 ppm due to hydrogen bonding interactions, and (b) the excellent sensitivity of ^1^H NMR, which allows very low solute concentrations to be used, thus avoiding the effects of concentration dependent aggregation processes [54,55,56,57,58,59,60,61,62,63,64].

2. DFT calculations of the large experimental solvent- and protonation-dependent chemical shifts in nitrogen in, e.g., heterocycles, amines and amides, oxygen in alcohols, carbonyls, amides, nucleic bases, organic acids and peracids, amino acids, and peptides can provide an excellent means to investigate complex solvatomer structures in terms of noncovalent (hydrogen bonding, donor–acceptor and van der Waals) interactions and cooperative phenomena [146,147,148,178,187,190]. Low solute concentrations, however, are needed to avoid the effects of concentration-dependent aggregation processes, which necessitate the use of ^1^H-^15^N HSQC/HMBC experiments and ^17^O-enriched compounds for enhanced sensitivity.

3. The solvent-dependent chemical shift variations in nuclei which belong to the above polar groups cannot be approximated via the polarizable continuum models, for example, PCM, CPCM, or IEF-PCM methods with UFF, UAHF or UAKS radii. Significantly improved results can be obtained either using a combination of molecular dynamics (explicit solvent model) and continuum models [51,114,173,187] or by manually placing explicit solvent molecules in the vicinity of potential polar or charged groups in combination with continuum models [55,59,60,61,62,63,64,72,95,107,109].

4. Significantly increasing the number of explicit solvent molecules through MD simulations results only in moderate improvement in the accuracy of computational chemical shifts; however, the energy profiles may be significantly affected by the solvation model used. It is recommended that only a limited number of explicit solvent molecules, around potential hydrogen bonding sites, should be taken into account [95].

5. Geometry optimization with the B3LYP hybrid DFT functional in combination with a basis set at least 6-31+G(d) would be sufficient for ^1^H NMR. For nitrogen and oxygen, the inclusion of a basis set with diffuse and polarization functions, such as 6-31+G(d,p), is recommended. The advantages of using more rigorous treatment of electron correlation, such as coupled-cluster theory (CSDCT), in the case, for example, of charged molecules, should be further investigated. It is important to highlight that although a significant error may exist for a given computational method, the use of the same method for the reference compound results in appreciable error cancellation [34].

6. The inclusion of explicit solvent molecules significantly increases the number of low-energy conformers, which necessitate extensive conformational searches. Once a number of low-energy solvatomers has been located, a Boltzmann analysis is used to determine the relative contribution of each solvatomer to the overall NMR spectrum. In practice, the situation is more complex since various species with different solvation numbers may be present. This should be handled on a case-by-case basis. For phenols, for example, in DMSO, acetone, and acetonitrile solutions, the use of 1:1 complexes was sufficient to obtain excellent agreement with the experimental ^1^H NMR chemical shift data [59,60,61,62,63,64]. On the contrary, in the case of alcohols, which are significantly less acidic than phenols, the presence of a fraction of free alcohol should be taken into account in acetone and acetonitrile solutions [89].

7. Zero-point vibrational corrections and relativistic effects on chemical shift calculations can be considered of minor importance for most practical cases, even for nitrogen and oxygen nuclei.

8. The structural characteristics of various low-energy solvatomers could be related to numerous multiparametric equations and empirical polarity scales [1,13,178]. Such studies have only sporadically appeared in the literature [176,177].

9. Computational chemical shifts in solvatomers are expected to provide important information on the nature of hydrogen bonding [60,62,208] and cooperativity.

10. DFT calculated activation energies can provide an excellent method for investigating the role of explicit solvent molecules in various atomistic reaction mechanisms [203,204]. Geometry optimization with the APFD functional in combination with a basis set at least 6-31+G(d) would be sufficient for ^1^H NMR.

The ongoing research will certainly improve the accuracy of the currently available experimental and computational methods, coupled with machine learning strategies and increasing computer power. It would be expected that this perspective will stimulate and provide some guidance for future research in the exciting field of NMR and DFT studies on atomic-level solvation phenomena in bioorganic molecules, natural products and model compounds.

## Figures and Tables

**Table 1:** Chemical shifts and Δδ/ΔΤ values of hydroxyl protons in phenol (**1**), 4-methylchatehol (**2**), and genkwanin (**3**), in DMSO-d_6_ ^a^, acetone-d_6_ ^b^, CD_3_CN ^c^, and CDCl_3_ ^d^, and their chemical shift differences at 298 K. Reproduced with permission from [59]. Copyright 2013, The Royal Society of Chemistry, London, UK.

Compound	δDMSO-d_6_	Δδ/ΔΤ ^e^	δAcetone-d_6_	Δδ/ΔΤ ^e^	Δδ(δDMSO-d_6_ -δAcetone-d_6_)	δCD_3_CN	Δδ/ΔΤ ^e^	Δδ(δDMSO-d_6_ -δCD_3_CN)	δCDCl_3_	Δδ/ΔΤ ^e^	Δδ(δDMSO-d_6_ -δCDCl_3_)
(**1**) C-1 OH	9.36	−5.4	8.29	−9.0	1.07	6.90	−6.7	2.46	4.65	−5.8	4.71
(**2**) C-1 OH	8.57	−6.9	(8.26) ^f^ 7.58	−8.9	0.99	6.42	−5.7	2.15	4.88	−5.9	3.69
9C-2 OH	8.71	−7.1	7.65	−9.2	1.06	6.52	−5.8	2.19	5.02	−5.7	3.69
(**3**) C-5 OH	12.95	−2.1	13.03	−1.4	−0.08	12.95	−0.8	0.00	12.93	^c^	0.02
C-4΄OH	10.56	−8.9	9.26	−8.9	1.30	7.70	−8.5	2.86	5.36	^c^	5.20

^a^ Concentrations C = 5 mM, ^b^ C = 5 mM, ^c^ C = 5 mM, and ^d^ C = 0.5 mM, except for genkwanin (**3**), in which the concentration could not be determined due to low solubility. ^e^ Expressed in parts per 10^9^ (ppb) per K. ^f^ The chemical shift in 90% acetone/10% acetone-d_6_.

**Table 2:** The calculated OH ^1^H NMR chemical shifts (relative to TMS, ppm) in 1:1 PhOH+solvent complexes with the gauge invariant atomic orbitals (GIAOs) method at the B3LYP/6-311++G (2d,p) level of theory in the gas phase, using the CPCM model. Reproduced with permission from [59]. Copyright 2013, The Royal Society of Chemistry, London, UK.

1:1 PhOH + Solvent Complex	B3LYP 6-31+G(d) Geometry Optimization, Gas Phase	B3LYP 6-31+G(d) Geometry Optimization, CPCM	B3LYP 6-311++G(d,p) Geometry Optimization, Gas Phase	B3LYP 6-311++G(d,p) Geometry Optimization, CPCM	Experimental Values
PhOH + CHCl_3_	3.96	4.61	3.85	4.49	4.65
PhOH + MeCN	6.43	6.80	6.42	6.79	6.90
PhOH + acetone	8.60	8.95	8.48	8.83, 8.71 ^a^	8.29
PhOH + DMSO	9.02	9.31	9.08	9.37	9.36

^a^ Calculations using the B3LYP/cc-pVTZ method.

**Table 3:** ^1^H NMR chemical shifts, δ(ppm), in chrysophanol (**1)**, emodin (**2**), and physcion (**3**), in CDCl_3_, acetone-d_6_, and DMSO-d_6,_ and their differences at 298 K [72].

Compound	Group	δ_CDCl3_	δ_acetone-d6_	Δδ (δ_acetone-d6_-δ_CDCl3_)	δ_DMSO-d6_	Δδ (δ_DMSO-d6_-δ_CDCl3_)
Chrysophanol (**1**)	C(1)–OH	12.11	12.03	−0.08	11.96	−0.15
	C(8)–OH	12.00	11.95	−0.05	11.87	−0.13
	C(4)–H	7.64	7.62	−0.02	7.56	−0.08
	C(5)–H	7.81	7.70	−0.11	7.71	−0.10
	C(3)–H	7.65	7.82	0.17	7.80	0.15
	C(7)–H	7.27	7.35	0.08	7.38	0.11
	C(2)–H	7.09	7.19	0.10	7.22	0.13
	C(6)–CH_3_	2.45	2.45	0.00	2.44	−0.01
Emodin (**2**)	C(1)–OH	12.26	12.21	−0.05	12.11	−0.15
	C(8)–OH	12.08	12.09	0.01	12.04	−0.04
	C(5)–H	7.61	7.58	−0.03	7.50	−0.11
	C(4)–H	7.26	7.27	0.01	7.18	−0.08
	C(7)–H	7.07	7.15	0.08	7.11	0.04
	C(2)–H	6.65	6.67	0.02	6.57	−0.08
	C(3)–OH	6.18	10.21	4.03	11.41	5.23
	C(6)–CH_3_	2.41	2.47	0.06	2.41	0.00
Physcion (**3**)	C(1)–OH	12.30	12.24	−0.06	12.17	−0.13
	C(8)–OH	12.10	12.05	−0.05	11.97	−0.13
	C(2)–H	6.67	6.80	0.13	6.68	0.01
	C(4)–H	7.35	7.28	−0.07	7.20	−0.15
	C(5)–H	7.61	7.59	−0.02	7.53	−0.08
	C(7)–H	7.06	7.16	0.10	7.20	0.14
	C(3)–OCH_3_	3.92	4.00	0.08	3.92	0.00
	C(6)–CH_3_	2.43	2.47	0.04	2.42	−0.01

**Table 4:** The results of the linear regression of calculated vs. experimental ^1^H NMR chemical shifts determined from various minimized geometries ^a^ and the X-ray structure. Reproduced with permission from [61]. Copyright 2016, Elsevier, Inc., Amsterdam, The Netherlands.

Hypericin Complex	Method	Correlation Coefficient (R^2^)	Mean Square Error	Slope
HyH + acetone 1:1	B3LYP/6-31+G(d) (gas phase)	0.9903 (0.9208) ^b^	0.2707 (2.1175) ^b^	1.0924 (1.0304) ^b^
	B3LYP/6-31+G(d) (CPCM)	0.9917 (0.8926) ^b^	0.2320 (2.8740) ^b^	1.070 (0.9928) ^b^
	TPSSh/TZVP (gas phase)	0.9966 (0.9167) ^b^	0.0942 (2.2277) ^b^	0.9888 (0.9276) ^b^
	TPSSh/TZVP (CPCM)	0.9946 (0.871) ^b^	0.1506 (3.4516) ^b^	0.9589 (0.8777) ^b^
	CAM-B3LYP (CPCM)	0.9901 (0.8771) ^b^	0.2781 (3.2863) ^b^	1.0701 (0.9852) ^b^
HyH + acetone 1:2	B3LYP/6-31+G(d) (gas phase)	0.9943 (0.9146) ^b^	0.1597 (2.284) ^b^	1.0978 (1.0299) ^b^
	B3LYP/6-31+G(d) (CPCM)	0.9982 (0.8673) ^b^	0.3288 (3.5511) ^b^	1.0788 (0.9885) ^b^
	TPSSh/TZVP (gas phase)	0.9973 (0.9238) ^b^	0.0763 (2.0393) ^b^	1.0049 (0.946) ^b^
	TPSSh/TZVP (CPCM)	0.9937 (0.877)	0.1753 (3.6063) ^b^	0.9755 (0.8904) ^b^
	CAM-B3LYP (CPCM)	0.9858 (0.8521) ^b^	0.3977 (3.9557) ^b^	1.0729 (0.9757) ^b^
Hy^-^	B3LYP/6-31+G(d) (gas phase)	0.9926	0.3324	0.9605
	B3LYP/6-31+G(d) (IEF-PCM in DMSO)	0.9981	0.0848	1.0561
	TPSSh/TZVP (gas phase)	0.998	0.0912	0.9079
	TPSSh/TZVP (IEF-PCM in DMSO)	0.9994	0.0271	0.9537
	CAM-B3LYP (IEF-PCM in DMSO)	0.9981	0.0833	1.0603
	X-ray crystal structure	0.9678	1.4428	0.7472

^a^ The case of HyH + DMSO (1:1) was not examined, since in DMSO solution, hypericin exists in the ionic form (see text). ^b^ The experimental value of δ = 8.2 ppm for the OH-3,4 protons used by Smirnov et al. [84] was taken into account (see text).

**Table 5:** Experimental ^1^H NMR (δ_exp_, ppm) and calculated (δ_calc_, ppm) at the B3LYP/6-311G+(2d,p) (CPCM DMSO) level, ^1^H NMR chemical shifts in conformers A, B, C, and D of methyl salicylate complexes with a discrete molecule of DMSO (Figure 19), with minimization of the structures at the ωB97XD/6-31G+d level. Reproduced with permission from [64]. Copyright 2018, Elsevier, Inc., Amsterdam, The Netherlands.

DFT		Energy ^a^	ΔG ^b^	H6	H5	H4	H3	CH_3_	OH
ωB97XD/631G+d	A	−1088.153109	0.0 (77.61%)	8.35	7.08	7.77	7.22	4.01	10.35
ωB97XD/631G+d	Β	−1088.151165	1.22 (9.90%)	8.26	7.14	7.84	7.35	3.89	10.91
ωB97XD/631G+d	C	−1088.150705	1.59 (5.30%)	8.15	7.09	7.73	7.22	3.96	9.72
ωB97XD/631G+d	D	−1088.150860	1.41 (7.18%)	8.00	7.14	7.70	7.15	3.89	10.04
Experimental				7.79	6.95	7.52	6.99	3.90	10.49

^a^ Atomic units (Hartree energy; the sum of electronic and thermal energy); ^b^ Gibbs free energy (kcal/mol) relative to conformer **A**; in parenthesis are the Boltzmann populations (%) of conformers **A** to **D**.

**Table 6:** Comparison of experimental and calculated ^1^H NMR shifts (ppm) for 1,4-diols, **1–6**, in benzene; shifts in CDCl_3_ and DMSO. Reproduced with permission from [88]. Copyright 2014, Wiley, Chichester, West Sussex, UK.

Diol	Proton	C_6_D_6_ (Experimental)	C_6_H_6_ (Calculated)	CDCl_3_ ^a^	DMSO ^b,c^
**1**	OH	1.07	1.20	—	4.35
	CH_2_	1.33	1.58	1.69	1.43
	CH_2_OH	3.29	3.69	3.71	3.39
**2**	OH1	1.25	1.14	—	4.35
	OH8	1.24	1.13	—	4.33
	CH	1.37	1.38	1.68	1.41
	CH	1.39	1.60	1.70	1.48
	CH	1.23	1.33	1.52	1.32
	CH	1.27	1.62	1.62	1.33
	CHOH	3.32	3.67	3.68	3.37
	CHOH	3.34	3.70	3.71	3.38
	CHOH	3.49	3.79	3.87	3.58
	Me	0.95	1.11	1.22	1.03
**3**	OH	1.36	1.35	—	4.33
	CH	1.30	1.51	1.61	1.40
	CH	1.32	1.40	1.55	1.29
	CHOH	3.51	3.79	3.85	3.55
	Me	0.97	1.10	1.22	1.03
**4**	OH	1.04	0.50	—	4.06
	CH_2_	1.40	1.50	1.58	1.37
	Me	1.05	1.11	1.24	1.06
**5**	OH	0.58	0.24	—	4.28
	CH-*trans*	1.28	1.56	1.66	1.41
	CH-*cis*	1.52	1.65	1.74	1.55
	CHOH	3.36	3.69	3.80	3.52
**6**	OH	0.59	0.24	—	4.42
	CH-*ax*	1.06	1.21	1.36	1.15
	CH-*eq*	1.65	1.89	1.98	1.74
	CHOH	3.25	3.56	3.68	3.36

^a^ Aromatic solvent-induced low frequency shift = 0.32 ± 0.08 ppm. ^b^ DMSO solvent-induced low frequency shift = 0.25 ± 0.05 ppm. ^c^ DMSO solvent-induced high frequency shift of OH proton shifts, relative to benzene = 3.28 ± 0.34 ppm.

**Table 7:** Proton NMR shifts (ppm) in alcohols in acetonitrile and acetone. Experimental values were obtained at 298 K, and computational results were obtained at the PBE0/cc-pVTZ//PBE0/6-311+G(d,p) level. Reproduced with permission from [89]. Copyright 2016, Wiley, Chichester, West Sussex, UK.

Alcohol	Proton	Acetonitrile			Acetone		
		δ_expt_	δ_comp_ ^a^	δ_comp_ ^b^	δ_expt_	δ_comp_ ^a^	δ_comp_ ^b^
Methanol	OH	2.13	1.62	2.69	3.08	3.61	4.61
	CH_3_	3.27	3.26	3.29	3.31	3.49	3.50
Ethanol	OH	2.43	2.28	3.27	3.34	3.99	4.85
	CH_2_	3.54	3.54	3.58	3.57	3.70	3.69
	CH_3_	1.12	0.99	0.99	1.12	1.13	1.20
Tert-butyl-alcohol	OH	2.36	2.09	3.00	3.18	3.95	4.80
	CH_3_	1.17	1.05	1.09	1.19	1.18	1.21

^a^ Alcohol/solvent complex in the gas phase. ^b^ Alcohol/solvent complex in solvent continuum.

**Table 8:** B3LYP/6-31G(d,p)-PCM-DMSO ^1^H NMR chemical shifts (in ppm) regression analysis for the rutin molecule [93].

Strs.	Regression Slope ^a^	Regression Intercept ^a^	Adjusted *R*^2^ (adj-R^2^) ^b^
**27**	0.952 (±0.041)	0.515 (±0.198)	0.973 (±1.342)
**28**	1.033 (±0.058)	−0.096 (±0.281)	0.954 (±2.698)
**30**	0.959 (±0.040)	0.408 (±0.195)	0.974 (±1.295)
**32**	0.960 (±0.025)	0.427 (±0.122)	0.990 (±0.506)
**34**	0.948 (±0.044)	0.499 (±0.214)	0.968 (±1.565)

^a^ Standard error values are given in parentheses; ^b^ residual sum of squares values are given in parentheses.

**Table 9:** The results of δ/σ correlation analysis of the data for *E* and *Z* isomers of *N*-methylformamide (NMF) and formamide (F) in CDCl_3_ solutions, as represented by RMSD [ppm] values, and deviations in the experimental ^1^H and ^13^C NMR chemical shifts in amide from the calculated values (δ_obs_–δ_calc_), assuming various solvation modes ^a^. Reproduced with permission from [107]. Copyright 2017, The American Chemical Society, Washington, DC, USA.

Solute	Solvation Mode ^b^	RMSD	HCO	${CH}_{3}^{c}$	N−H ^c^	RMSD	C=O	CH_3_
*E*-NMF	0S	0.175	0.13	0.14	0.78	1.37	4.62	1.61
*E*-NMF	2S	0.094	0.18	0.11	0.33	1.14	3.05	1.01
*E*-NMF	0.90(2S) + 0.10(D)	0.051	0.09	0.11	−0.03	1.08	2.49	0.99
*Z*-NMF	0S	0.198	0.08	0.13	0.91	1.24	3.91	0.83
*Z*-NMF	2S	0.124	0.11	0.10	0.53	0.99	1.64	−0.06
*Z*-NMF	0.74(2S) + 0.26(D′)	0.058	0.15	0.13	0.00	0.98	1.59	0.06
F	0S	0.321	0.02	0.79 *^E^*	1.26 *^Z^*	1.26	3.97	
F	2S	0.210	0.08	0.48 *^E^*	0.82 *^Z^*	1.01	1.75	
F	D	0.564	−0.42	0.67 *^E^*	−2.37 *^Z^*	1.35	−4.57	
F	D′	0.313	0.23	−1.04 *^E^*	1.08 *^Z^*	1.02	1.82	
F	0.41(2S) + 0.25(D) + 0.34(D′)	0.044	−0.08	0.00 *^E^*	0.03 *^Z^*	0.94	0.18	

^a^ The data for the amides have been augmented by the appropriate data for the reference compounds. ^b^ *n*S: solute molecule solvated by *n* solvent molecules; D: doubly solvated cyclic dimer of the solute; D’: doubly solvated chain dimer of solute; coefficients denote population parameters; the population parameters were adjusted during analyses of ^1^H NMR data, and then used as fixed parameters in appropriate analyses of ^13^ C NMR data. ^c^ ^*E*^, ^*Z*^ deviations concern ^*E*^ and ^*Z*^ N−H hydrogens.

**Table 10:** The results of δ/σ correlation analysis of the data for *E* and *Z* isomers of N-methylformamide (NMF) and formamide (F) in DMSO-d_6_ solutions, as represented by RMSD [ppm] values, and deviations of the experimental ^1^H and ^13^C NMR chemical shifts in the amide from the calculated values (δ_obs_−δ_calc_), assuming various solvation modes ^a^. Reproduced with permission from [107]. Copyright 2017, The American Chemical Society, Washington, DC, USA.

Solute	Solvation Mode ^b^	RMSD	HCO	${CH}_{3}^{d}$	N−H ^d^	RMSD ^c^	C=O	CH_3_
*E*-NMF	0S	0.500	−0.16	−0.14	2.38	1.22	2.48	0.66
*E*-NMF	NH·S	0.053	−0.02	0.01	−0.09	1.21	2.44	0.65
*E*-NMF	0.04(0S) + 0.96(NH·S)	0.050	−0.03	0.01	0.00	1.22	2.44	0.88
*Z*-NMF	0S	0.523	−0.26	−0.14	2.49	1.13	1.34	0.00
*Z*-NMF	NH·S	0.082	−0.09	0.06	−0.27	1.13	1.24	0.44
*Z*-NMF	0.11(0S) + 0.89(NH·S)	0.057	−0.12	0.05	0.00	1.13	1.25	0.39
F	0S	0.623	−0.37	1.81 *^E^*	2.28 *^Z^*	1.26	2.78	
F	N(H·S)_2_	0.213	−0.04	−0.19 *^E^*	−0.90 *^Z^*	1.16	1.41	
F	0.25(0S) + 0.75(N(H·S)_2_)	0.090	−0.18	0.26 *^E^*	−0.17 *^Z^*	1.18	1.75	
F	0.31(NH *^E^*·S) + 0.19(NH *^Z^*·S) + 0.50(N(H·S)_2_)	0.062	−0.17	0.00 *^E^*	0.00 *^Z^*	1.17	1.58	

^a^ The data for the amides have been augmented by the appropriate data for the reference compounds. ^b^ S stands for DMSO molecule; 0S denotes the free amide molecule; coefficients denote population parameters. ^c^ the population parameters were adjusted during analyses of ^1^H NMR data, and then used as fixed parameters in appropriate analyses of ^13^ C NMR data. ^d^ *^E^*, *^Z^* deviations concern *^E^* and *^Z^* N−H hydrogens.

**Table 11:** The hydrogen bond lengths (in Å) in uracil derivatives solvated specifically at N-H (and O-H) hydrogens by DMSO molecules, as calculated using the B3LYP/6-311++G(2d,p), PCM(DMSO, UAKS) method. Reproduced with permission from [109]. Copyright 2017, The American Chemical Society, Washington, DC, USA.

Solvatomer	1-NH···O	3-NH···O
U·2DMSO	1.765	1.835
DHU·2DMSO	1.879	1.890
T·2DMSO	1.777	1.845
*ax*-DHT·2DMSO	1.874	1.883
*eq*-DHT·2DMSO	1.879	1.892
HMU·3DMSO ^a^	1.832	1.801

^a^ OH···O distance 1.785 Å. Molecular geometry optimization assuming the frozen geometry of DMSO and using the STO-3G basis set for the methyl group atoms.

**Table 12:** Populations of free solute molecules (*p*_0_), monosolvates 1-NH···DMSO (*p*_1_), 3-NH···DMSO (*p*_3_), and disolvates (*p*_13_) in DMSO solutions and solvation degrees of particular NH hydrogens (*p*_s1_ = *p*_1_ + *p*_13_, *p*_s3_ = *p*_3_ + *p*_13_) as determined on the basis of ^1^H NMR chemical shifts and calculated magnetic shielding constants of NH and CH hydrogens ^a^. Reproduced with permission from [109]. Copyright 2017, The American Chemical Society, Washington, DC, USA.

Solute	*Po*	*P*_1_	*P*_3_	*P*_13_	*P*_s1_	*P*_s3_
U	0.06 ± 0.01	0.17 ± 0.02	0.20	0.57	0.74	0.77
T	0.06 ± 0.01	0.22 ± 0.02	0.15	0.57	0.79	0.72
HMU	0.06 ± 0.01	0.21 ± 0.02	0.07	0.70	0.91	0.77
DHU	0.06 ± 0.01	0.13 ± 0.02	0.26	0.55	0.68	0.81
DHT	0.06 ± 0.01	0.13 ± 0.02	0.26	0.54	0.67	0.80

^a^ In the case of HMU, all species were solvated at the OH group by an additional DMSO molecule.

**Table 13:** Calculated δ(COOH) chemical shifts in caproleic acid (CA), oleic acid (OA), α-linoleic acid (ALA), EPA, and DHA, with a discrete solvation molecule of DMSO (in parentheses are the experimental chemical shifts in DMSO-d_6_ solution at 298 K). Reproduced with permission from [113]. Copyright 2023, Elsevier, Inc., Amsterdam, The Netherlands.

FFA	Intermolecular Interaction	δ(COOH) (ppm)	Complexation Energy (kcal/Mole–Gas Phase)
CA	COO-H···DMSO	13.4 (11.94)	−18.0 ^a^
CA dimer parallel	COO-H···DMSO	14.4, 13.9 ^b^	−15.7 ^c,d^
CA dimer antiparallel	COO-H··· DMSO	14.1, 14.2 ^b^	15.9 ^c,d^
OA	COO-H···DMSO	13.4 (11.94)	
ALA	COO-H··· DMSO	13.4 (11.95)	
EPA	COO-H···DMSO	13.1 (12.01)	
DHA	COO-H···DMSO	13.5 (12.08)	

^a^ ωB97X-D/aug-cc-pVDZ; ^b^ ωB97X-D /6-31+G(d,p); ^c^ ωB97X-D /6-311++G(2d,2p); ^d^ the complexation energy was calculated between the two DMSO-CA aggregates and not between a molecule of CA and a DMSO molecule.

**Table 14:** Experimental ^1^H chemical shift δ_H_ values (in ppm) for 1(FHF)^−^, 2(H_5_O_2_)^+^, and 3(PyHPy)^+^, and computational results using various approximations. Reproduced with permission from [51]. Copyright 2025, The American Chemical Society, Washington, DC, USA.

	1 (FHF)^−^	2 (H_5_O_2_)^+^	3 (PyHPy)^+^
	**Experimental values**		
	16.6	21.3	21.73
	**Computed values** **I. Stationary QC** **I.1. Solvation effects**		
Vacuum	18.4	21.3	21.6
Implicit solvent	18.3	21.2	20.0
Explicit solvent	18.3	19.7	20.8
Cluster–continuum	18.2	21.0	20.1
	**I.2. Nuclear delocalization** ***1D SE***		
Vacuum	17.2	19.7	23.0
Implicit solvent	16.9	19.4	21.9
		*2D SE*	
Vacuum	17.6	20.2	23.4
Implicit solvent	17.3	19.9	22.1
		*3D SE*	
Vacuum	17.6	20.1	22.7
Implicit solvent	17.3	19.9	20.8
	**I.3. Relativistic effects**		
Levy−Leblond	17.9	21.0	21.7
Four-component (4c)	17.7	21.0	21.7
Vacuum	18.4	II. Thermal motion 20.9	24.2
Explicit solvent	17.7	20.2	22.0

**Table 15:** ^15^N NMR chemical shifts in pyridine and pyridazine, calculated at the GIAO-KT3/pcS-3//pc-2 level, taking into account solvent effects ^a^. Reproduced with permission from [146]. Copyright 2014, Wiley, Chichester, West Sussex, UK.

			^15^N NMR Chemical Shift (δ, ppm) ^b^		
Compound	Solvent	Gas Phase	IEF-PCM	Supermolecule (1:1)	Experiment
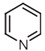	C_6_H_6_	−56.1 (5.0)	−61.6 (0.5)	−64.6 (3.5)	−61.1
	CHCl_3_	−56.1 (12.6)	−65.5 (3.2)	−75.0 (6.3)	−68.7
	CH_2_Cl_2_	−56.1 (9.2)	−67.4 (2.1)	−65.2 (0.1)	−65.3
	(CH_3_)_2_CO	−56.1 (5.7)	−68.8 (7.0)	−68.3 (6.5)	−61.8
	CH_3_OH	−56.1 (25.0)	−69.3 (11.8)	−87.7 (6.6)	−81.1
	C_2_H_5_OH	−56.1 (24.2)	−69.0 (11.3)	−82.1 (1.8)	−80.3
	H_2_O	−56.1 (28.2)	−69.7 (14.6)	−82.2 (2.1)	−84.3
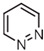	*c*-C_6_H_12_	45.6 (10.3)	36.8 (1.5)	38.8 (3.5)	35.3
	CCl_4_	45.6 (16.2)	35.8 (6.4)	35.3 (5.9)	29.4
	C_6_H_6_	45.6 (17.8)	35.7 (7.9)	29.5 (1.7)	27.8
	CHCl_3_	45.6 (26.6)	29.0 (10.0)	21.1 (2.1)	19.0
	CH_2_Cl_2_	45.6 (25.4)	25.8 (5.6)	24.8 (4.6)	20.2
	(CH_3_)_2_CO	45.6 (19.7)	23.3 (2.6)	23.5 (2.4)	25.9
	CH_3_OH	45.6 (39.3)	22.5 (16.2)	6.3 (0.0)	6.3
	C_2_H_5_OH	45.6 (35.1)	22.9 (12.4)	10.2 (0.3)	10.5
	H_2_O	45.6 (51.8)	21.7 (27.9)	9.3 (15.5)	−6.2

^a^ Geometry optimizations were performed at the MP2/6-311++G(d.p) level with solvent effects taken into account within the IEF-PCM. ^b 15^N NMR chemical shifts are reported in ppm relative to nitro–methane (in parentheses are the absolute errors (ppm) of calculated chemical shifts vs. experimental values).

**Table 16:** Protonation ^15^N NMR shifts in compounds **1–4** (Figure 45) calculated at the GIAO-T3/pcS-3 level, using different solvation models ^a^. Reproduced with permission from [151]. Copyright 2015, Wiley, Chichester, West Sussex, UK.

Compound	Name	Protonation Shift				
		Gas Phase	Polarizable Continuum Model	Supermolecule in Polarizable Continuum	Counter Ion in Polarizable Continuum	Experiment
**1**	Pyridine	−139.3	−119.4	−99.9	−87.7	−107.4
**2**	*N*-methylimidazole	−114.9	−92.3	−84.5	−64.1	−85.6
**3**	Acetone oxime	−153.9	−134.1	−129.3	−108.8	−117.5
**4**	Triethylamine	+23.7	+18.0	+9.8	+4.3	+9.9

^a^ All protonation shifts are in ppm.

**Table 17:** ^17^O isotropic shieldings (ppm) for acetone in water, computed in clusters with an increasing number of solvent molecules, averaged over all the selected MD snapshots. In parentheses are the computations with the 6-311+G(d,p) basis set. Reference values: basis set 6-31G(d) = −323.1 ppm, and basis set 6-311+G(d,p) = −356.8 ppm [171].

No. of Solvent Molecules	Without PCM	With PCM
0	0.0 (0.0)	−50.2 (−64.6)
2	−59.2 (−54.2)	−92.6 (−94.2)
5	−75.3 (−67.7)	−96.0 (−92.5)
10	−81.4	
20	−87.2	
40	−97.3	

**Table 18:** Τhe mPW1PW91/aug-pcS-2 magnetic shielding σ (ppm) constants of uracil (**a**) and 5-fluorouracil (**b**) in water solution. In parentheses are the results obtained using B3LYP [183].

Compound		Gas	PCM ^a^	ASEC(PCM) ^b^	ASEC(Iter) ^c^	Exp. ^d^
Uracil						
O_2_	σ	−7.8 (−11.6)	35.5 (33.0)	50.6 (47.3)	57.2 (54.1)	55.5
O_4_	σ	−113.5 (−117.1)	−41.1 (−43.1)	−17.2 (−20.4)	−13.5 (−16.4)	−13.5
5-Flurouracil						
O_2_	σ	3.6 (−0.2)	43.7 (41.0)	49.4 (45.9)	56.5 (53.3)	57.5
O_4_	σ	−93.5 (−96.4)	−25.5 (−27.0)	−13.6 (−16.4)	−12.6 (−15.1)	−6.5

^a^ For this calculation, e = 55.6 was used, which is the dielectric constant of water at 100 °C; ^b^ the electronic polarization of the solute was included by using PCM; ^c^ the solute electronic polarization effect was obtained by using an iterative procedure; ^d^ experimental values from Ref. [184].

**Table 19:** Activation enthalpy ($\Delta H_{exp}^{‡}$), activation entropy (− TΔ$S_{exp}^{‡}$), and Gibbs activation energy ($\Delta G_{exp}^{‡}$) for H/D exchange reaction of the H-8 and H-6 protons in taxifolin (2.5 mM) at various pD values and phosphate buffer concentrations [203,204]. Reproduced with permission from [204]. Copyright 2025, The Royal Society, London, UK.

Compound	Buffer Concentration	pD	H-8			H-6		
			$\Delta H_{exp}^{‡}$(kcal mol^−1^)	$-\;T\Delta S_{exp}^{‡}$ (kcal mol^−1^)	$\Delta G_{exp}^{‡}$(kcal mol^−1^)	$\Delta H_{exp}^{‡}$(kcal mol^−1^)	$-\;T\Delta S_{exp}^{‡}$ (kcal mol^−1^)	$\Delta G_{exp}^{‡}$(kcal mol^−1^)
Taxifolin	25 mM	6.0	20.31 ± 1.57	4.70 ± 0.79	25.01	18.91 ± 1.18	5.62 ± 0.59	24.53
	25 mM	7.6	18.79 ± 1.59	5.80 ± 0.80	24.59	14.01 ± 1.47	9.17 ± 0.82	23.18
	50 mM	7.6	19.34 ± 1.18	5.05 ± 0.65	24.39	20.63 ± 1.26	2.24 ± 0.49	22.87
	25 mM	9.6	18.43 ± 1.79	6.24 ± 0.72	24.67	16.67 ± 0.55	6.15 ± 0.55	22.81
	50 mM	9.6	19.62 ± 0.77	4.07 ± 0.65	23.69	16.96 ± 0.54	5.51 ± 0.58	22.46
	1 M	9.6	15.96 ± 1.22	7.98 ± 0.78	23.94	11.76 ± 0.75	10.38 ± 0.98	22.14
Phloroglucinol			H-2,4,6					
	25 mM	6.9	17.46 ± 0.30	3.50 ± 0.09	20.96			
	25 mM	7.9	16.05 ± 0.78	3.69 ± 0.26	19.74			
	25 mM	8.9	16.55 ± 1.15	2.86 ± 0.29	19.41			

**Table 20:** Selected computed Gibbs activation energy ($\Delta G_{calc}^{‡}$, kcal mol^−1^) for neutral and ionic forms of phloroglucinol and taxifolin for various molecular H_2_O solvation species and computational methods. In parentheses are the Boltzmann populations. Reproduced with permission from [204]. Copyright 2025, Royal Society, London, UK.

Neutral Phloroglucinol	+2H_2_O	+3H_2_O			Exp.	
		**“in-out”**	**“in-in”** **“in-in A”**		20.96	
APFD/6-31+G(d)	20.59	26.28 (10.94%)	20.13 (58.52%) 19.75 (30.51%)			
B3LYP/6-31+G(d)/GD3BJ	23.15	27.52 (14.19%)	21.66 (46.80%) 21.54 (38.19%)			
PBE0/6-31+G(d)/GD3BJ	23.16	27.76 (9.65%)	21.65 (51.47%) 21.48 (38.75%)			
ωB97XD/6-31+G(d)	26.19	30.86 (17.12%)	25.08 (52.04%) 24.76 (30.36%)			
CAM-B3LYP/6-31+G(d)/GD3BJ	24.68	29.71 (16.10%)	23.99 (46.39%) 23.62 (37.37%)			
**Neutral taxifolin**	**+2H_2_O**		**+3H_2_O**		**Exp.**	
	C-6	C-8	C-6	C-8	C-6	C-8
APFD/6-31+G(d)	20.79	20.77	22.98	23.55	24.53	25.01
B3LYP/6-31+G(d)/GD3BJ	20.81	21.93	25.63	24.72		
PBE0/6-31+G(d)/GD3BJ	20.80	21.28	25.18	24.67		
ωB97XD/6-31+G(d)	25.08	24.19	27.38	27.37		
CAM-B3LYP/6-31+G(d)	24.78	25.67	29.12	28.81		
CAM-B3LYP/6-31+G(d)/GD3BJ	22.69	23.59	27.26/27.05	27.66		
**Ionic phloroglucinol**	**+2H_2_O**			**+3H_2_O**	**Exp.**	
	on OH	C=O (A)	C=O (B)		19.74	
APFD/6-31+G(d)	11.83	19.15	^a^	11.26		
B3LYP/6-31+G(d)/GD3BJ	13.01 (0.26%)	19.91 (33.85%)	19.86 (65.89%)	11.63		
PBE0/6-31+G(d)/GD3BJ	13.05	19.71	^a^	11.48		
ωB97XD/6-31+G(d)	15.21 (0.37%)	22.16 (41.71%)	21.37 (57.92%)	14.07		
CAM-B3LYP/6-31+G(d)	14.39 (0.32%)	21.69 (60.95%)	20.58 (38.73%)	13.36		
CAM-B3LYP/6-31+G(d)/GD3BJ	14.08 (0.30%)	21,32 (39.49%)	20.47 (60.20%)	12.95		
**Ionic taxifolin**	**+2H_2_O**	**+3H_2_O**		**Exp.**		
	^a^	C-6	C-8	C-6	C-8	
B3LYP/6-31+G(d)/GD3BJ	^a^	23.83	24.27	23.18	24.59	
PBE0/6-31+G(d)/GD3BJ	^a^	24.76	26.00			
ωB97XD/6-31+G(d)	^a^	24.51	26.18			
CAM-B3LYP-D/6-31+G(d)	^a^	23.44	22.80			

^a^ A transition state could not be determined.

## Data Availability

Not applicable.
